# OCaR1 endows exocytic vesicles with autoregulatory competence by preventing uncontrolled Ca^2+^ release, exocytosis, and pancreatic tissue damage

**DOI:** 10.1172/JCI169428

**Published:** 2024-04-01

**Authors:** Volodymyr Tsvilovskyy, Roger Ottenheijm, Ulrich Kriebs, Aline Schütz, Kalliope Nina Diakopoulos, Archana Jha, Wolfgang Bildl, Angela Wirth, Julia Böck, Dawid Jaślan, Irene Ferro, Francisco J. Taberner, Olga Kalinina, Staffan Hildebrand, Ulrich Wissenbach, Petra Weissgerber, Dominik Vogt, Carola Eberhagen, Stefanie Mannebach, Michael Berlin, Vladimir Kuryshev, Dagmar Schumacher, Koenraad Philippaert, Juan E. Camacho-Londoño, Ilka Mathar, Christoph Dieterich, Norbert Klugbauer, Martin Biel, Christian Wahl-Schott, Peter Lipp, Veit Flockerzi, Hans Zischka, Hana Algül, Stefan G. Lechner, Marina Lesina, Christian Grimm, Bernd Fakler, Uwe Schulte, Shmuel Muallem, Marc Freichel

**Affiliations:** 1 Institute of Pharmacology, Heidelberg University, Heidelberg, Germany.; 2 DZHK (German Centre for Cardiovascular Research), partner site Heidelberg/Mannheim, Heidelberg, Germany.; 3 Comprehensive Cancer Center München, Chair for Tumor Metabolism, Klinikum rechts der Isar, Technical University of Munich, School of Medicine and Health, Munich, Bavaria, Germany.; 4 Epithelial Signaling and Transport Section, National Institute of Dental and Craniofacial Research, National Institutes of Health, Bethesda, USA.; 5 Institute for Physiology, University of Freiburg, Freiburg, Germany.; 6 Walther-Straub-Institut für Pharmakologie und Toxikologie, Ludwig-Maximilians-Universität München, Munich, Germany.; 7 Instituto de Neurociencias de Alicante, Universidad Miguel Hernández–Consejo Superior de Investigaciones Científicas, Sant Joan d’Alacant, Spain.; 8 Helmholtz Institute for Pharmaceutical Research Saarland (HIPS), Helmholtz Centre for Infection Research (HZI), Saarbrücken, Germany.; 9 Institut für Pharmakologie und Toxikologie, Universität Bonn, Bonn, Germany.; 10 Experimental and Clinical Pharmacology and Toxicology, Center for Molecular Signaling (PZMS), Saarland University, Homburg, Germany.; 11 Institute of Molecular Toxicology and Pharmacology, Helmholtz Center Munich, German Research Center for Environmental Health, Neuherberg, Germany.; 12 University Hospital Heidelberg, Department of Medicine III: Cardiology, Angiology and Pneumology, Heidelberg, Germany.; 13 Institut für Experimentelle und Klinische Pharmakologie und Toxikologie, Fakultät für Medizin, Albert-Ludwigs-Universität Freiburg, Freiburg, Germany.; 14 Center for Integrated Protein Science Munich (CIPS-M) and Center for Drug Research, Department of Pharmacy, Ludwig-Maximilians-Universität München, and DZHK (German Centre for Cardiovascular Research), partner site Munich Heart Alliance, Munich, Germany.; 15 Walter Brendel Centre of Experimental Medicine, Biomedical Center, Institute of Cardiovascular Physiology and Pathophysiology, Medical Faculty, Ludwig-Maximilians-Universität München, Planegg-Martinsried, Germany.; 16 Institute for Molecular Cell Biology, Center for Molecular Signaling (PZMS), Universität des Saarlandes, Homburg, Germany.; 17 Institute of Toxicology and Environmental Hygiene, Technical University Munich, School of Medicine, Munich, Germany.; 18 Immunology, Infection and Pandemic Research (IIP), Fraunhofer Institute for Translational Medicine and Pharmacology (ITMP), Munich, Germany.

**Keywords:** Cell biology, Calcium signaling, Ion channels, Lysosomes

## Abstract

Regulated exocytosis is initiated by increased Ca^2+^ concentrations in close spatial proximity to secretory granules, which is effectively prevented when the cell is at rest. Here we showed that exocytosis of zymogen granules in acinar cells was driven by Ca^2+^ directly released from acidic Ca^2+^ stores including secretory granules through NAADP-activated two-pore channels (TPCs). We identified OCaR1 (encoded by *Tmem63a*) as an organellar Ca^2+^ regulator protein integral to the membrane of secretory granules that controlled Ca^2+^ release via inhibition of TPC1 and TPC2 currents. Deletion of OCaR1 led to extensive Ca^2+^ release from NAADP-responsive granules under basal conditions as well as upon stimulation of GPCR receptors. Moreover, OCaR1 deletion exacerbated the disease phenotype in murine models of severe and chronic pancreatitis. Our findings showed OCaR1 as a gatekeeper of Ca^2+^ release that endows NAADP-sensitive secretory granules with an autoregulatory mechanism preventing uncontrolled exocytosis and pancreatic tissue damage.

## Abstract 2

OCaR1 endows secretory granules an autoregulatory competence to prevent uncontrolled exocytosis and pancreatic tissue damage in mice.

## Graphical abstract

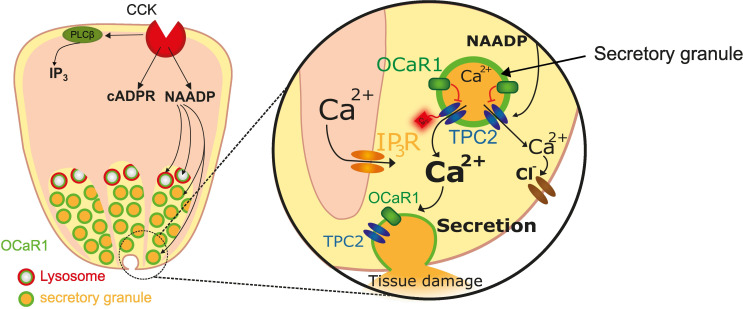

## Introduction

In the pancreas, secretion of digestive enzymes in response to neurohumoral stimulation mediated by agonists such as cholecystokinin (CCK) or by the neurotransmitter acetylcholine derived from cholinergic nerve terminals occurs in a Ca^2+^-dependent way from acinar cells. This process requires an increase in intracellular Ca^2+^ concentration ([Ca^2+^]_i_) in close spatial proximity to secretory vesicles, which is mediated by Ca^2+^ release from intracellular organelles at the apical cell pole (1, 2). Pancreatic acinar cells have a remarkably large amount of Ca^2+^ stored in both the endoplasmic reticulum (ER) and acidic Ca^2+^ stores (3), which mainly comprise endo-lysosomes and secretory granules (4). ER Ca^2+^ stores are located in the basal part of the acinar cells with extensions protruding into the apical area, while acidic stores are located in the secretory granular area of the cell (5). Ca^2+^ release from both intracellular compartments can be triggered by stimulation of CCK receptors and is mediated by inositol trisphosphate (IP_3_), cyclic ADP-ribose (cADPR) and nicotinic acid adenine dinucleotide phosphate (NAADP), respectively (6–8). Furthermore, local NAADP-induced Ca^2+^ release is considered to initiate Ca^2+^-induced Ca^2+^ release from the ER through IP_3_ and ryanodine receptors, giving rise to more global Ca^2+^ signals (9). Although a number of NAADP-responsive Ca^2+^-releasing channels have been identified in acidic stores, their role in acinar CCK-evoked secretion under physiological conditions and in disease is poorly understood. Thus, NAADP-elicited Ca^2+^ release could be reduced by inactivation of two-pore channels (TPCs) (10), but was unaltered in acinar cells lacking transient receptor potential mucolipin 1 (TRPML1) (11). The response to CCK stimulation is additionally modulated by channels in the plasma membrane, e.g., by TRPC3, which contributes to refilling of the intracellular Ca^2+^ stores (12), and by calcium release–activated channels (CRACs) composed of ORAI proteins maintaining the long-lasting cytosolic Ca^2+^ plateau evoked by high CCK concentrations (13).

The release of Ca^2+^ must be tightly controlled, since aberrant Ca^2+^ signaling or Ca^2+^ homeostasis led to pancreatitis, the most prevalent exocrine pancreatic disease (14). In acute pancreatitis (AP), premature intracellular activation and release of pancreatic zymogens (15) trigger autodigestion, organ damage, inflammation, edema, and multiple local and systemic complications. Although typically a self-limiting disease, AP is associated with substantial mortality per se. Repetitive occurrence of AP may promote progression to chronic pancreatitis (CP) characterized by irreversible organ degeneration, fibrosis, and exocrine insufficiency (16, 17). Dissecting the mechanisms of Ca^2+^ regulation in acinar cells in vitro and in vivo can, therefore, provide insights into pancreatic disease and eventually offer new therapeutic options.

In search of further transmembrane proteins involved in Ca^2+^ release from acidic organelles in acinar cells, we identified TMEM63A, a member of the yet poorly characterized TMEM63 family. Using high-resolution organellar proteomics, fluorescence microscopy, and expression in transgenic mouse models through the native promoter, we show that TMEM63A is primarily present in endo-lysosomes and secretory granules of acinar cells. We found TMEM63A to function as an organellar Ca^2+^ regulator of organellar Ca^2+^ release channels, and thus termed the protein OCaR1. In acinar cells from *OCaR1^–/–^* mice, conversion of local Ca^2+^ oscillations to global Ca^2+^ signals was enhanced, leading to an increase in spontaneous and regulated exocytosis, followed by acinar cell destruction and aggravation of CP. We show that the exaggerated Ca^2+^ signals arise from acidic Ca^2+^ stores, and that OCaR1 counteracts the activity of the organellar Ca^2+^ channels TPC1 and TPC2, thus operating as a gatekeeper in Ca^2+^ release from secretory granules and protecting the pancreas from pancreatitis-associated tissue damage.

## Results

### Identification of OCaR1 and its subcellular localization to secretory granules.

**Figure 1:**
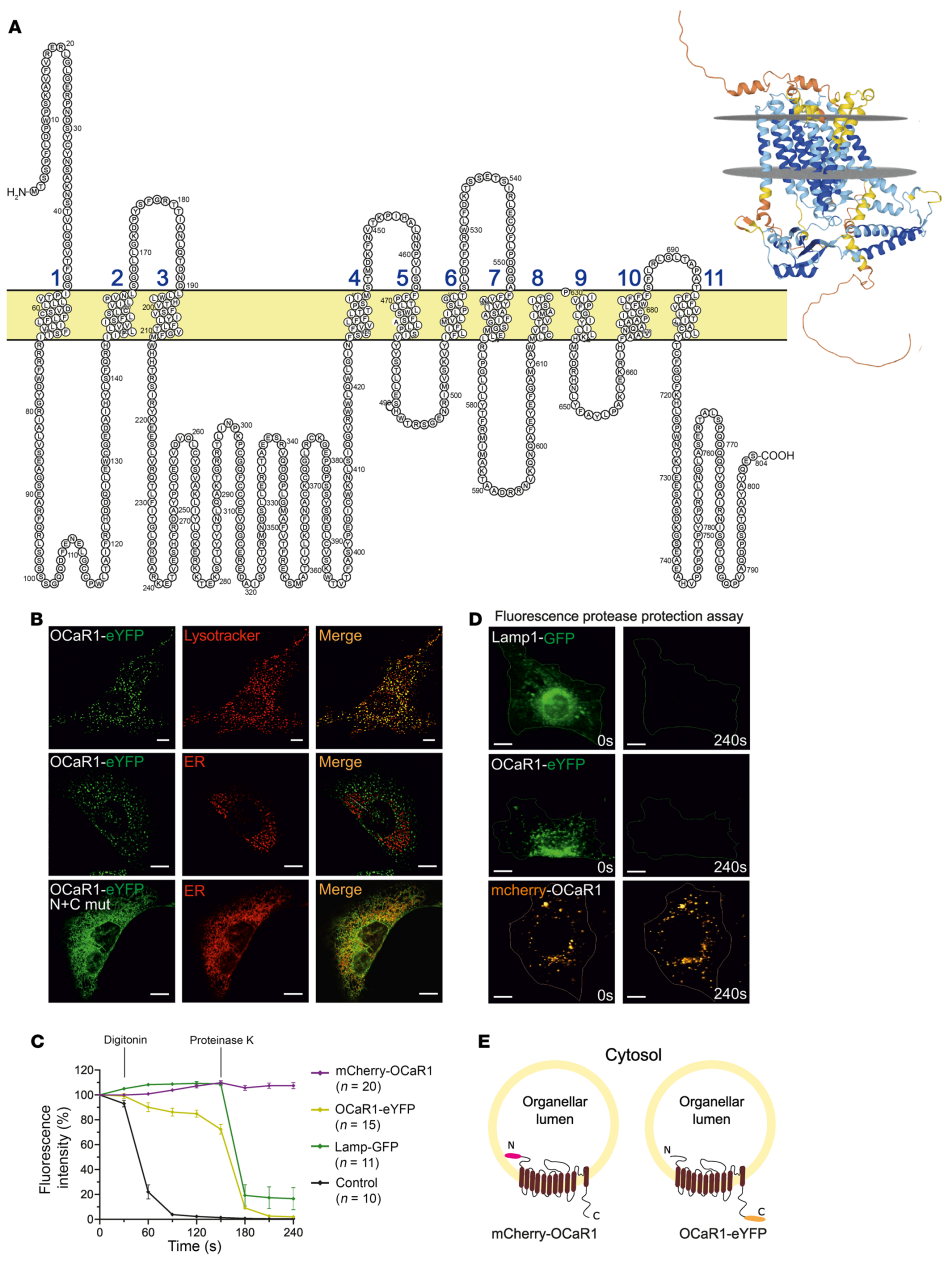
OCaR1 exhibits 11 transmembrane domains and is localized in acidic organelles with a cytosolic C- and a luminal N-terminus when expressed in MEF cells. (**A**) Predicted transmembrane topology of OCaR1. Right: Predicted structure of OCaR1. The protein is colored according to the pLDDT (predicted local distance difference test) score that reflects the reliability of prediction, with more reliably predicted regions in blue. The position of membrane is indicated with gray mesh. The intraluminal side is oriented toward the top side of the image. (**B**) Colocalization analysis of OCaR1-eYFP expressed by adenoviral transfer with acidic organelles (LysoTracker Red, top, *n* = 5) or ER (middle, *n* = 8) in MEF cells. Colocalization analysis of N- and C-terminal double di-leucine mutants (N+C mut) of OCaR1-eYFP with ER (bottom, *n* = 5) in MEFs. Scale bars: 10 μm. (**C** and **D**) Fluorescence protease protection assay to analyze the orientation of the fluorescent protein fused to OCaR1 relative to the lysosomal membrane. After permeabilization with digitonin (10 μM), all freely diffusing proteins are washed out of the cell (Control, GFP) whereas proteins that are protected by the membrane of an organelle keep their fluorescence. Proteinase K (50 μg/mL) disrupts the fluorescence pattern of fusion proteins that expose their fluorophore into the cytosol (Lamp-eYFP, OCaR1-eYFP) but not when in the organellar lumen (mCherry-OCaR1). Scale bars: 10 μm. (**E**) Schematic model of the OCaR1 topology relative to the membrane of acidic organelles.

In a similarity search using sequence motifs conserved among the TRP channel proteins, we identified the genes *Tmem63a*, *Tmem63b*, and *Tmem63c* (Supplemental Figure 1A; supplemental material available online with this article; https://doi.org/10.1172/JCI169428DS1) encoding members of a family of proteins well conserved in eukaryotes. BLAST (https://blast.ncbi.nlm.nih.gov/) searches against the full protein sequences of TMEM63A did not identify considerable homology with any other protein in mammals, while modest sequence similarity with the plant OSCA (18) proteins was detected (24.3% over 550 positions in comparison with OSCA1.2 from *Oryza sativa*). According to recently determined structures of human TMEM63A (19, 20), the protein comprises 11 hydrophobic stretches compatible with membrane-spanning helices (Figure 1A and Supplemental Figure 1B). Based on subcellular localization and function (shown below), we termed the protein encoded by *Tmem63a* OCaR1 in the following, and the related proteins OCaR2 and OCaR3 (Supplemental Figure 1C). OCaR1 transcripts were abundant in most tissues analyzed (Supplemental Figure 2).

For investigation of its subcellular localization, we first fused OCaR1 C-terminally with eYFP and probed its expression in HEK293 cells, where it localized to a vesicular compartment inside the cell (Supplemental Figure 3A). After adenoviral transduction in mouse embryonic fibroblasts (MEFs), the OCaR1-eYFP fusion protein was found to colocalize best with the marker LysoTracker Red (Figure 1B, top) as indicated by correlation analysis (Pearson’s coefficient *r* = 0.76 ± 0.02). Furthermore, 74% of the Lamp1-positive signals colocalized with OCaR1 according to the Manders coefficient (M1 = 0.74 ± 0.06). Additionally, 73% of OCaR1 proteins were colocalizing with lysosomes in MEF cells (M2 = 0.73 ± 0.09, *n* = 5; Supplemental Figure 3B). In contrast, no overlap was detected with markers of the ER (Figure 1B, middle), Golgi apparatus, mitochondria, or peroxisomes (Supplemental Figure 3, B–E). Two short, putative sorting motifs, present in various endo-lysosomal proteins (21), can be found in the N-terminus [DXXLL] (D_188_NDLL) and C-terminus [DER]XXXL[LI] (R_573_LPGLI) of OCaR1. After exchanging leucine and isoleucine residues to alanine in both motifs (L191A/L192A and L577A/I578A, OCaR1^Lyso-mut^-eYFP), OCaR1 was retained in the ER, thus confirming the importance of these sorting motifs for localization of OCaR1 to acidic organelles.

The topology of OCaR1 was further analyzed by a fluorescence protease protection assay (22). To this end, OCaR1-eYFP was analyzed in parallel with the N-terminal fusion construct mCherry-OCaR1, GFP-fused Lamp1 (Lamp-GFP), and free/soluble GFP as control. Fluorescence of all fusion constructs was insensitive to permeabilization of the cell membrane by digitonin, in contrast to soluble GFP that was rapidly washed out (Figure 1C). Application of proteinase K, however, rapidly eliminated the cytosol-exposed fluorophores fused to both Lamp1 (Figure 1, C and D) and OCaR1 (Figure 1C and Figure 1D, middle), but failed to affect fluorescence of mCherry-OCaR1 (Figure 1C and Figure 1D, bottom). Together, these results indicated that the C-terminus of OCaR1 was located in the cytosol, while its N-terminus was facing the lumen of the acidic vesicles (Figure 1E), in line with the reported structure of OCaR1 (19, 20).

**Figure 2:**
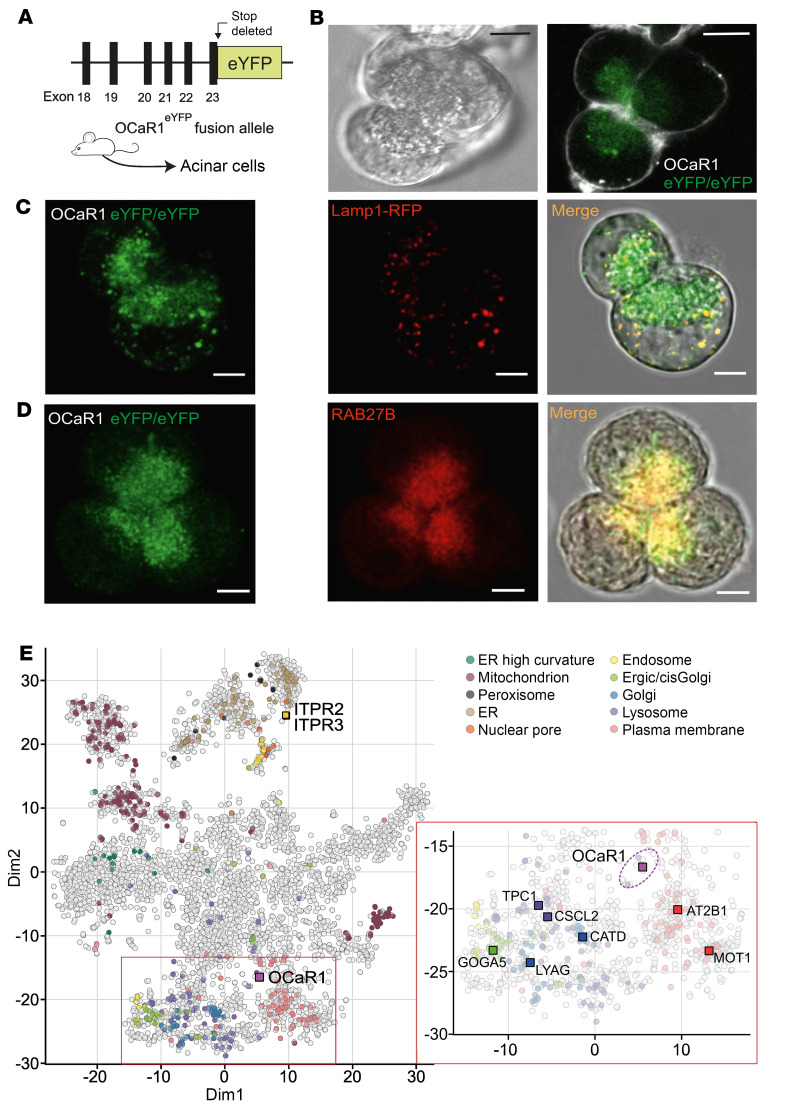
Endogenous OCaR1 is localized in acidic and secretory granules at the apical pole of acinar cells. (**A**) *OCaR1-eYFP* knock-add-on mice express an OCaR1-eYFP fusion protein generated by in-frame introduction of eYFP-encoding sequences in exon 23 of the OCaR1 (*Tmem63a*) gene and removal of the stop codon (for details see Supplemental Figure 5). (**B**) Subcellular localization of OCaR1-eYFP fusion proteins expressed under control of the endogenous OCaR1 promoter in acinar cells of homozygous *OCaR1-eYFP* knock-add-on mice (*OCaR1^eYFP/eYFP^*, 6 mice). Scale bars: 10 μm. (**C**) Endogenous OCaR1 proteins colocalize with Lamp1. Representative confocal microscopy images (from 7 mice) of OCaR1-eYFP (left) and Lamp1-RFP fluorescence (middle) in acinar cells isolated from *OCaR1^eYFP/eYFP^* mice derived from *OCaR1^eYFP/eYFP^* zygotes transduced with lentiviral vectors encoding Lamp1-RFP (6.8 integrants per genome). Right: Merge of both fluorescence channels with the corresponding DIC contrast bright-field image. Scale bars: 5 μm. (**D**) OCaR1 protein colocalizes with Rab27B. Representative confocal microscopy images of OCaR1-eYFP and Rab27B fluorescence in acinar cells from *OCaR1^eYFP/eYFP^* mice using anti-Rab27B and anti-GFP antibodies (*n* = 13 cells, 3 mice). Scale bars: 5 μm. (**E**) t-Distributed stochastic neighbor embedding (tSNE) plot visualizing the result of an organellar proteomic analysis of primary mouse acinar cells. Each circle represents a protein with its distribution profile over 42 subcellular fractions (see Methods); color-coded superimposition denotes verified markers for the indicated subcellular compartments (71), with prominent representatives for each highlighted by their UniProt short name. The inset (right) is a zoom into the proximity of OCaR1 (CSCL1) with the closest proteins (circled in purple) being implicated in secretory vesicle function (see main text).

Next, we aimed to analyze the subcellular localization of OCaR1 in native polarized acinar cells (23). To this end, we generated mice expressing OCaR1-eYFP by lentiviral transduction of mouse zygotes (Supplemental Figure 4A) and an OCaR1^eYFP^ knock-add-on mouse strain by gene targeting (Supplemental Figure 5 and Figure 2A). As illustrated for freshly isolated pancreatic acinar cells in Figure 2B, OCaR1 was detected in vesicular structures near the apical pole, but also appeared (to some extent) in the periapical area in either model (Figure 2B and Supplemental Figure 4, A–C).

Expression of Lamp1-RFP (via injection of lentiviral particles into OCaR1^eYFP^ zygotes; see Methods) to label endo-lysosomes for live-cell imaging revealed colocalization of OCaR1 in Lamp1-positive vesicles (Figure 2C; *r* = 0.29 ± 0.04). Although 66% of lysosomal signals colocalized with OCaR1 (M1 = 0.66 ± 0.09), approximately 93% of OCaR1-positive vesicles were Lamp1 negative (M2 = 0.07 ± 0.02; *n* = 7 offspring) (Figure 2C). Staining for the secretory granule marker Rab27B (24) (Figure 2D) in acinar cells of OCaR1^eYFP^ mice revealed prominent colocalization (*r* = 0.65 ± 0.02; M1 = 0.68 ± 0.08; M2 = 0.63 ± 0.07; *n* = 13 cells, *n* = 3 offspring), with 63% of the OCaR1 colocalizing with Rab27B.

As orthogonal and more unbiased assessment of the subcellular localization of endogenous OCaR1, we used organellar proteomics (25–27). Primary acinar cells isolated from WT mouse pancreas were subjected to stepwise homogenization and separation by ultracentrifugation into 42 fractions (see Methods). Quantitative mass spectrometry identified more than 4,800 proteins with abundance profiles, resulting in a well-structured organellar map of preferred protein localizations after dimensionality reduction (t-distributed stochastic neighbor embedding [t-SNE] plot; Figure 2E). Superimposition of verified subcellular markers confirmed the established localization of a selected set of membrane proteins (Figure 2E, inset; also see legend) and placed OCaR1 between plasma membrane and intracellular (mainly endosomal/secretory) vesicles, indicating its dynamic subcellular (re)distribution during endo-/exocytosis. Indeed, the nearest neighbors of OCaR1 in the t-SNE plot (Figure 2E, inset, circled in purple) included several proteins implicated in vesicle endo-/exocytosis, such as receptor-type tyrosine-protein phosphatase N2 (PTPR2), sorting nexins 3 and 4 (SNX3 and SNX4), the storage vesicle recruitment factor C2CD5, and the Ras-related protein Rab22A.

Together, these results indicated preferred localization of OCaR1 to acinar secretory granules and pointed to a functional role in acinar cell secretion.

### Deletion of OCaR1 leads to increased spontaneous Ca^2+^ release from acidic compartments and aberrant exocytosis in acinar cells.

**Figure 3:**
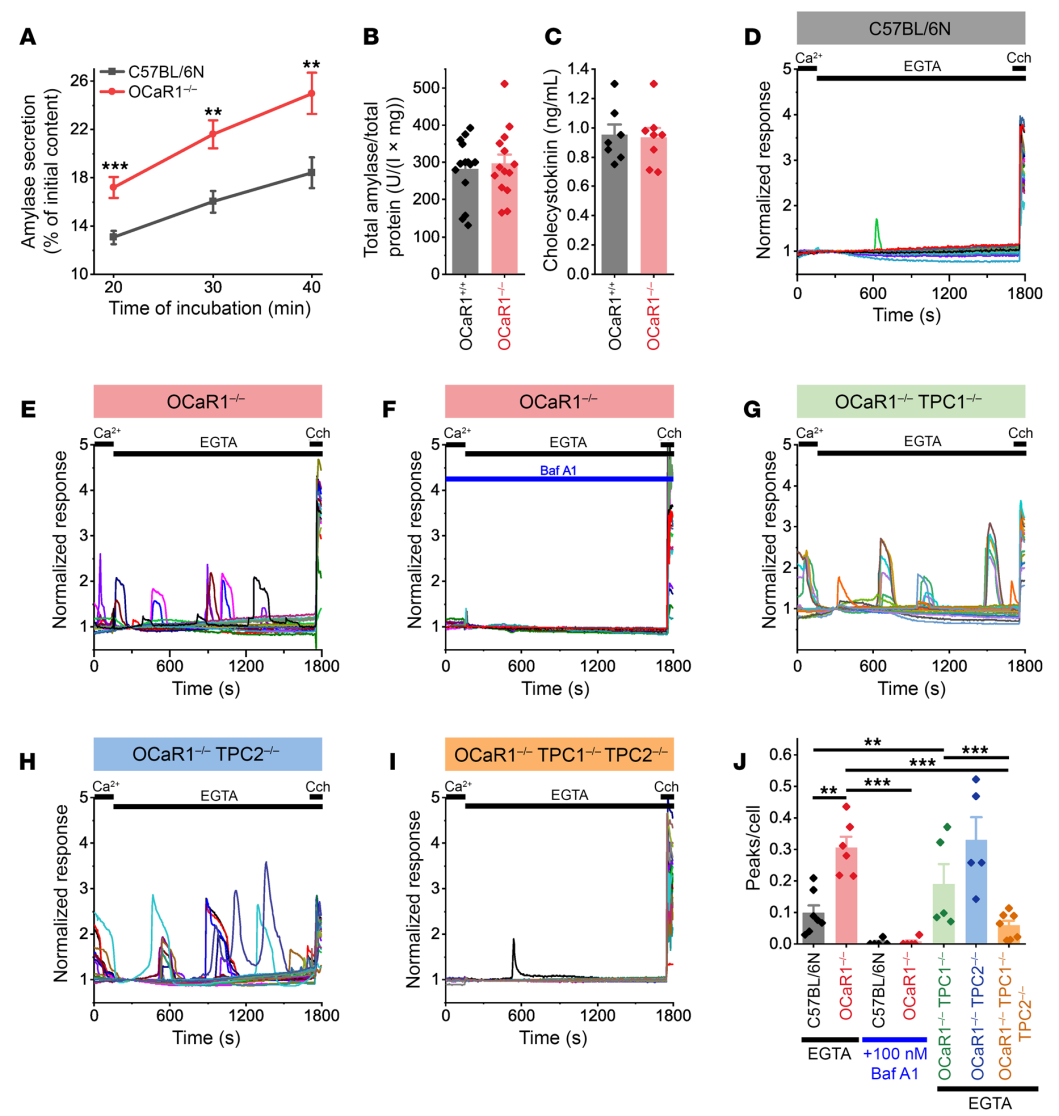
Acinar amylase hyperexocytosis leading to elevated plasma levels of digestive enzymes is associated with increased spontaneous Ca^2+^ transients in *OCaR1^–/–^* mice. (**A**) Analysis of spontaneous amylase exocytosis from acinar cells. Acini from C57BL/6N (*n* = 9, black) and *OCaR1^–/–^* (*n* = 8, red) mice were incubated for the indicated time periods and the fraction of released amylase measured. (**B**) The total amylase content was unaltered in acinar cells of *OCaR1^–/–^* compared with littermate *OCaR1^+/+^* mice (*n* = 14 preparations per genotype). (**C**) Plasma cholecystokinin levels are unchanged in *OCaR1^–/–^* mice (*OCaR1^+/+^*, *n* = 7; *OCaR1^–/–^*, *n* = 8). (**D** and **E**) Representative traces (25 cells per genotype, normalized to the value at 295 seconds) of Fura-2 fluorescence in C57BL/6N (**D**) and *OCaR1^–/–^* (**E**) acinar cells in Ca^2+^-free physiological solutions. Carbachol (CCh; 10 μM) was applied at the end of the experiment as a positive control. (**F**–**I**) Spontaneous Ca^2+^ transients in acinar cells of *OCaR1^–/–^* mice can be inhibited by 90-minute preincubation with bafilomycin A1 (Baf A1) (C57BL/6N, *n* = 5; *OCaR1^–/–^*, *n* = 5) (**F**) or by additional deletion of both TPC1 and TPC2 (*OCaR1^–/–^ TPC2^–/–^ TPC1^–/–^* triple knockout) (**G**–**I**). (**J**) Comparison of frequency of spontaneous Ca^2+^ oscillations between genotypes of the experiments in **D**–**I** (C57BL/6N, *n* = 7; *OCaR1^–/–^*, *n* = 6; C57BL/6N + Baf A1, *n* = 5; *OCaR1^–/–^* + Baf A1, *n* = 5; *OCaR1^–/–^ TPC1^–/–^*, *n* = 5; *OCaR1^–/–^ TPC2^–/–^*, *n* = 5; *OCaR1^–/–^ TPC1^–/–^ TPC2^–/–^*, *n* = 7). Statistical analysis was done by 2-tailed Student’s *t* test (**A**–**C**) or 1-way ANOVA with Bonferroni’s post hoc test (**J**) (**P < 0.01, ***P < 0.001).

Next, we investigated the in vivo function of OCaR1 by means of targeted deletion of the *Tmem63a* gene in mice (see Methods and Supplemental Figure 6). *OCaR1^–/–^* mice were viable, showed no obvious phenotypic abnormalities, and revealed no difference in body weight (Supplemental Figure 7A); moreover, the *OCaR1* (*Tmem63a*)–null allele segregated according to the expected Mendelian frequency (Supplemental Table 1). Blood analysis, however, showed that *OCaR1^–/–^* mice displayed significantly increased plasma amylase and lipase levels (amylase: WT 1,971 ± 98 U/L, *OCaR1^–/–^* 2,796 ± 139 U/L, *n* = 4, *P* = 0.0028, 2-tailed Student’s *t* test; lipase: WT 25 ± 1 U/L, *OCaR1^–/–^* 85 ± 17 U/L, *n* = 4, *P* = 0.0105, 2-tailed Student’s *t* test) together with normal glucose tolerance (Supplemental Figure 7B) suggesting involvement of OCaR1 in exocrine pancreatic function. In addition to aberrant elevation of amylase and lipase in the plasma of *OCaR1^–/–^* mice, we found that amylase activity was significantly increased in the media of isolated *OCaR1^–/–^* acini (Figure 3A), suggesting cell-autonomous deregulation of digestive enzyme exocytosis. This elevated spontaneous amylase release was not caused by increased acinar cell death (94.2% and 94.4% viability of *OCaR1^–/–^* and control mice, respectively, in 2 independent trypan blue stainings, not shown) or a change in the total amylase content of the cells (Figure 3B). Notably, the plasma levels of CCK were not different between *OCaR1^+/+^* and littermate *OCaR1^–/–^* mice (Figure 3C), excluding that elevated plasma amylase levels in *OCaR1^–/–^* mice were caused by enhanced levels of this major secretagogue and neurohumoral activity of the *OCaR1^–/–^* mice.

As exocytosis of pancreatic enzymes is strictly Ca^2+^ dependent, the aforementioned results raised the question of whether changes in [Ca^2+^]_i_ account for uncontrolled exocytosis in *OCaR1^–/–^* acinar cells. Spontaneous Ca^2+^ oscillations, which are rare events in unstimulated WT acinar cells, could be noted at significantly higher frequencies in *OCaR1^–/–^* cells both in the presence (Supplemental Figure 9A) and in the absence of external Ca^2+^ (Figure 3, D, E, and J, and Supplemental Figure 8, A–C).

After incubation with bafilomycin A1, which disrupts Ca^2+^ storage within acidic organelles, spontaneous Ca^2+^ events in *OCaR1^–/–^* cells were completely abolished (Figure 3, F and J), suggesting that the exaggerated Ca^2+^ oscillations originated from intracellular acidic Ca^2+^ stores such as endo-lysosomes and secretory granules. Furthermore, spontaneous Ca^2+^ transients caused by OCaR1 deletion were significantly reduced by deletion of the two-pore channels TPC1 and TPC2 (*OCaR1-Tpc1-Tpc2* triple-knockout mice), but not by knockout of either TPC1 or TPC2 (Figure 3, G–J, and littermate controls in Supplemental Figure 8, D–L). Likewise, deletion of either TPC1 or TPC2 alone did not affect Ca^2+^ release in WT acinar cells (Supplemental Figure 14, A–I). Furthermore, Ca^2+^ oscillations in *OCaR1^–/–^* cells were not affected by the deletion of TRPML1, another endo-lysosomal ion channel (Supplemental Figure 8, M–O). Moreover, we observed that the increase in Ca^2+^ oscillations in OCaR1-deficient acinar cells was not affected by incubation of the cells with GSK-7975A, an inhibitor of ORAI and CRAC channels (Supplemental Figure 10).

In sum, OCaR1 controls spontaneous Ca^2+^ oscillations mediated by TPC1 and TPC2 and exocytosis in acinar cells.

### OCaR1 inhibits TPC1 and TPC2 channel activity and critically modulates CCK-triggered Ca^2+^ release from secretory granules.

**Figure 4:**
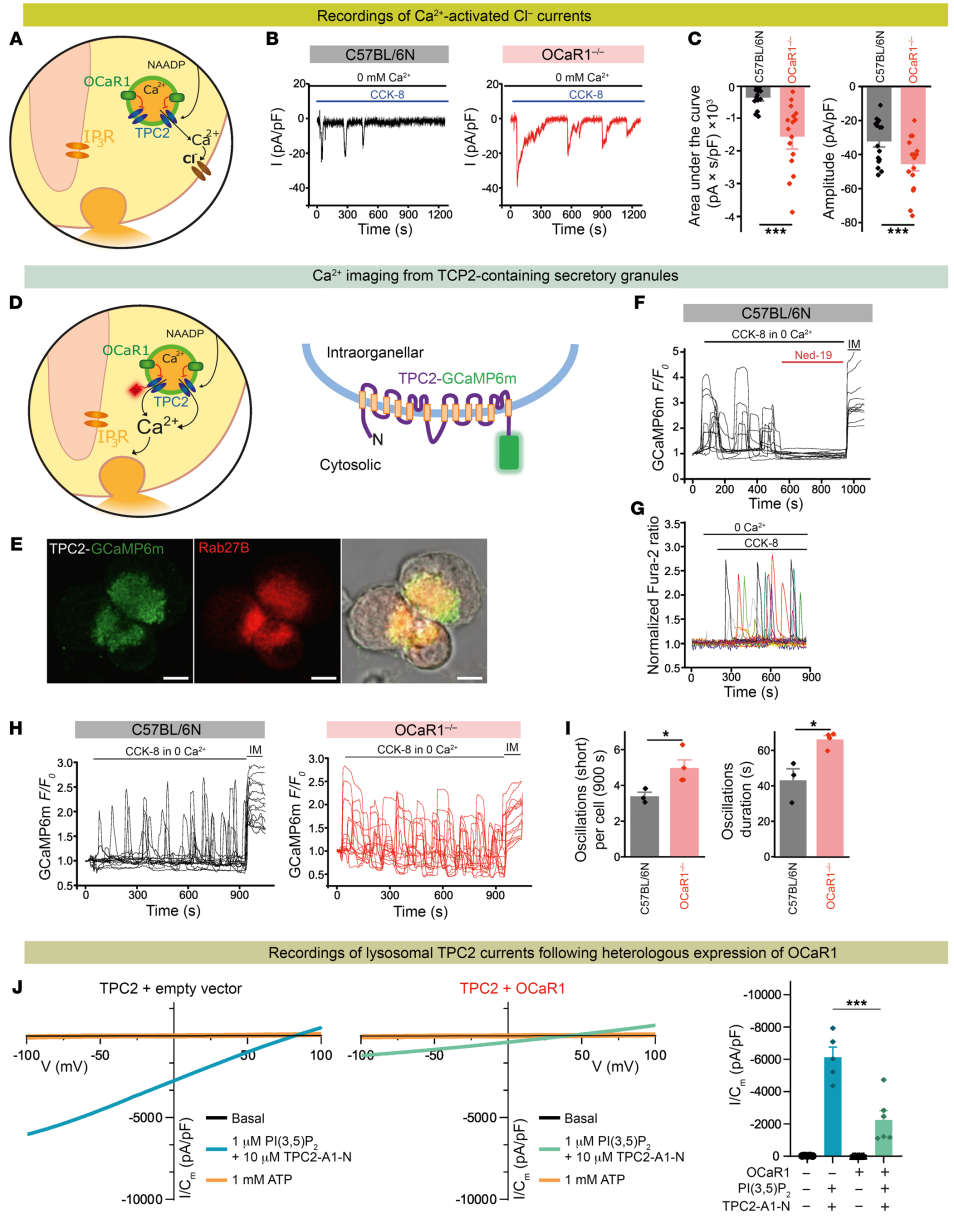
Enhanced cholecystokinin-evoked exocytosis is mediated by Ca^2+^ release events from TPC2-containing vesicles. (**A**) Model indicating localization of OCaR1 to secretory granules and lysosomes in acinar cells. NAADP-mediated Ca^2+^ release via TPC2 channels (blue) is monitored by the activity of adjacent Ca^2+^-activated chloride channels (brown). (**B**) CCK-8–induced (2 pM) Ca^2+^-activated Cl^–^ currents in C57BL/6N and *OCaR1^–/–^* acinar cells in the absence of extracellular Ca^2+^. (**C**) AUC of inward currents over 20 minutes and average amplitude (*n* = 16 cells, 5 mice per genotype). (**D**) Model of C-terminal fusion construct of TPC2 and GCaMP6m. (**E**) Representative (17 images, 3 mice) confocal microscopy images of TPC2-GCaMP6m–expressing acinar cells costained with anti-GFP and anti-Rab27B and merged image. Scale bars: 5 μm. (**F**) CCK-8 (2 pM)–induced oscillations in GCaMP6m fluorescence in acinar cells from mice expressing TPC2-GCaMP6m. Acute application of Ned-19 (50 μM) abolishes CCK-8–induced responses (*n* = 99). (**G**) Representative traces (*n* = 20, from 100 cells per mouse, 5 mice) from CCK-8–evoked global Ca^2+^ oscillations in WT acinar cells. (**H**) Representative traces (*n* = 20) of CCK-8 (2 pM)–evoked oscillations in GCaMP6m fluorescence in TPC2-GCaMP6m–expressing acinar cells. (**I**) Frequency and duration of Ca^2+^ oscillations detected by TPC2-GCaMP6m during stimulation with 2 pM CCK-8 of acinar cells from C57BL/6N (*n* = 77 cells, 3 mice) and *OCaR1^–/–^* mice (*n* = 135 cells, 4 mice). (**J**) Current densities elicited by coapplication of PI(3,5)P_2_ and TPC2-A1-N in vacuolin-enlarged endo-lysosomal vesicles isolated from HEK293 cells overexpressing *mTPC2-RFP* with or without *OCaR1-YFP*. Activated currents were blocked with 1 mM ATP (overlapping the basal current) (*n* = 4–6). Right: Average current densities at –100 mV. Comparison was done by 2-sample *t* test (**C** and **I**) or 1-way ANOVA and Tukey’s post hoc test (**J**) (*P < 0.05, ***P < 0.001). IM, 10 μM ionomycin.

Next, we investigated the role of the Ca^2+^ pool in secretory granules for their own regulated exocytosis evoked by the secretagogue CCK. At application of low concentration of the bioactive CCK fragment CCK-8 (300 fM), *OCaR1^–/–^* acinar cells exhibited enhanced amplitude and duration of intracellular Ca^2+^ oscillations measured in the absence of Ca^2+^ (Supplemental Figure 9, B–E), which was not affected by the ORAI1/CRAC channel blocker GSK-7975A. Similarly, in the presence of extracellular Ca^2+^, GSK-7975A had no effect on the enhanced amplitude and duration of the observed intracellular Ca^2+^ oscillations in *OCaR1^–/–^* acinar cells (Supplemental Figure 11). Application of a high CCK-8 concentration (1 nM) evoked the expected large Ca^2+^ transients with a long-lasting plateau in WT cells that was reduced by GSK-7975A (Supplemental Figure 11, L–R) and mediated by ORAI1/CRAC channels as published (13). Under these conditions *OCaR1^–/–^* acinar cells exhibited Ca^2+^ oscillations that were superimposed on the Ca^2+^ transients and were GSK-7975A insensitive (Supplemental Figure 11, N, O, and R). Notably, the occurrence of CCK-8–evoked intracellular Ca^2+^ oscillations in *OCaR1^–/–^* acinar cells could be significantly reduced by preincubation with bafilomycin A1 (Supplemental Figure 9, F–H), indicating again an origin in acidic Ca^2+^ stores. Ca^2+^-activated Cl^–^ currents represent a faithful reporter of local Ca^2+^ spikes derived from secretory granules next to the apical plasma membrane in acinar cells that are engaged by local and short-lasting Ca^2+^ oscillations (28) (Figure 4A). We recorded such Ca^2+^-activated Cl^–^ currents for 20 minutes upon CCK receptor stimulation with physiological agonist concentration (2 pM CCK-8) in WT and *OCaR1^–/–^* acinar cells (Figure 4B). Amplitude and AUC of these currents (Figure 4, B and C) were profoundly increased in *OCaR1^–/–^* compared with WT acinar cells, indicating an extended CCK-evoked Ca^2+^ release in the absence of OCaR1 (3).

As CCK, besides activating the IP_3_- and cADPR-mediated Ca^2+^ release from the ER, is thought to also trigger an increase in intracellular Ca^2+^ via NAADP acting on TPC channels in endo-lysosomes (11), we next turned to direct measurement of spatially restricted Ca^2+^ release events from TPC2-containing granules at the apical pole of acinar cells. For this purpose, we transduced murine zygotes with a lentivirus expressing the genetically encoded Ca^2+^ sensor GCaMP6m fused to the cytoplasmic C-terminus of the TPC2 protein (TPC2-GCaMP6m; Figure 4D), which localized to secretory granules as shown by colocalization with Rab27B (Figure 4E). In fact, CCK-8 evoked robust Ca^2+^ oscillations at the cytosolic face of the granules in the absence of extracellular Ca^2+^ (Figure 4F). These oscillations were completely abolished by Ned-19 (Figure 4F), an established antagonist of NAADP (29). In contrast, Ca^2+^ release from the ER following application of either carbachol (100 nM, causing IP_3_-mediated Ca^2+^ release) or the SERCA inhibitor thapsigargin (Tg; 500 nM) did not trigger fluorescence of TPC2-GCaMP6m at the surface of the secretory granules (Supplemental Figure 12A). Notably, though, all stimulants (CCK-8, carbachol, and Tg) caused a measurable increase in average cytosolic Ca^2+^, with Tg increasing cytosolic Ca^2+^ to an even higher level than CCK-8 (Figure 4G and Supplemental Figure 12B). Comparative analysis of WT and *OCaR1^–/–^* acinar cells expressing the TPC2-GCaMP6m sensor unveiled a significant increase in both frequency and duration of granular Ca^2+^ release in *OCaR1^–/–^* acinar cells (Figure 4, H and I). These results indicated that OCaR1 effectively regulates NAADP-mediated Ca^2+^ release from endo-lysosomes and secretory granules and suggested direct action on TPC channels as the underlying mechanism.

We therefore tested the effect of OCaR1 on both TPC1 and TPC2 channel activity by recording of TPC1- or TPC2-mediated currents in enlarged endo-lysosomes from COS-7 cells or HEK293 cells expressing TPC1 or TPC2 alone or together with either OCaR1 or VAMP7 (Supplemental Figure 13, A and B). In both preparations, OCaR1 significantly reduced the amplitudes of TPC1 and TPC2 currents activated by the endogenous ligand PI(3,5)P_2_ (Supplemental Figure 13A and Supplemental Figure 14, J–O), while VAMP7, another endo-lysosomal membrane protein used as negative control, failed to do so (Supplemental Figure 13B). A similar result was obtained when TPC2 currents were activated by TPC2-A1-N, a synthetic TPC2 agonist mimicking NAADP, in combination with PI(3,5)P_2_, or by the agonist TPC2-A1-P (Figure 4J and Supplemental Figure 14, M–O). In contrast, OCaR1 did not inhibit TRPML1-dependent lysosomal currents activated by PI(3,5)P_2_ (Supplemental Figure 13A).

Notably, the observed changes in Ca^2+^ signaling in OCaR1-deficient cells were not caused by changes in the expression levels of channel or transporter genes involved in regulation of cytoplasmic Ca^2+^ homeostasis. Thus, transcriptome analysis of the pancreas of WT and *OCaR1^–/–^* mice showed that genes of IP_3_ receptors type 1, 2, and 3, ryanodine receptors Ryr1, Ryr2, and Ryr3 (Supplemental Figure 15), or TPC1, TPC2, and TRPML1 were unchanged in *OCaR1^–/–^* mice.

In summary, these results indicate that OCaR1 effectively inhibits TPC1 and TPC2 channel activity and controls the CCK-8–stimulated release of Ca^2+^ from TPC2-containing secretory granules in acinar cells. Moreover, they unveil the importance of granular Ca^2+^ release in pancreatic acinar cells for Ca^2+^ homeostasis and regulated exocytosis.

### OCaR1 prevents uncontrolled exocytosis and pancreatic tissue damage.

**Figure 5:**
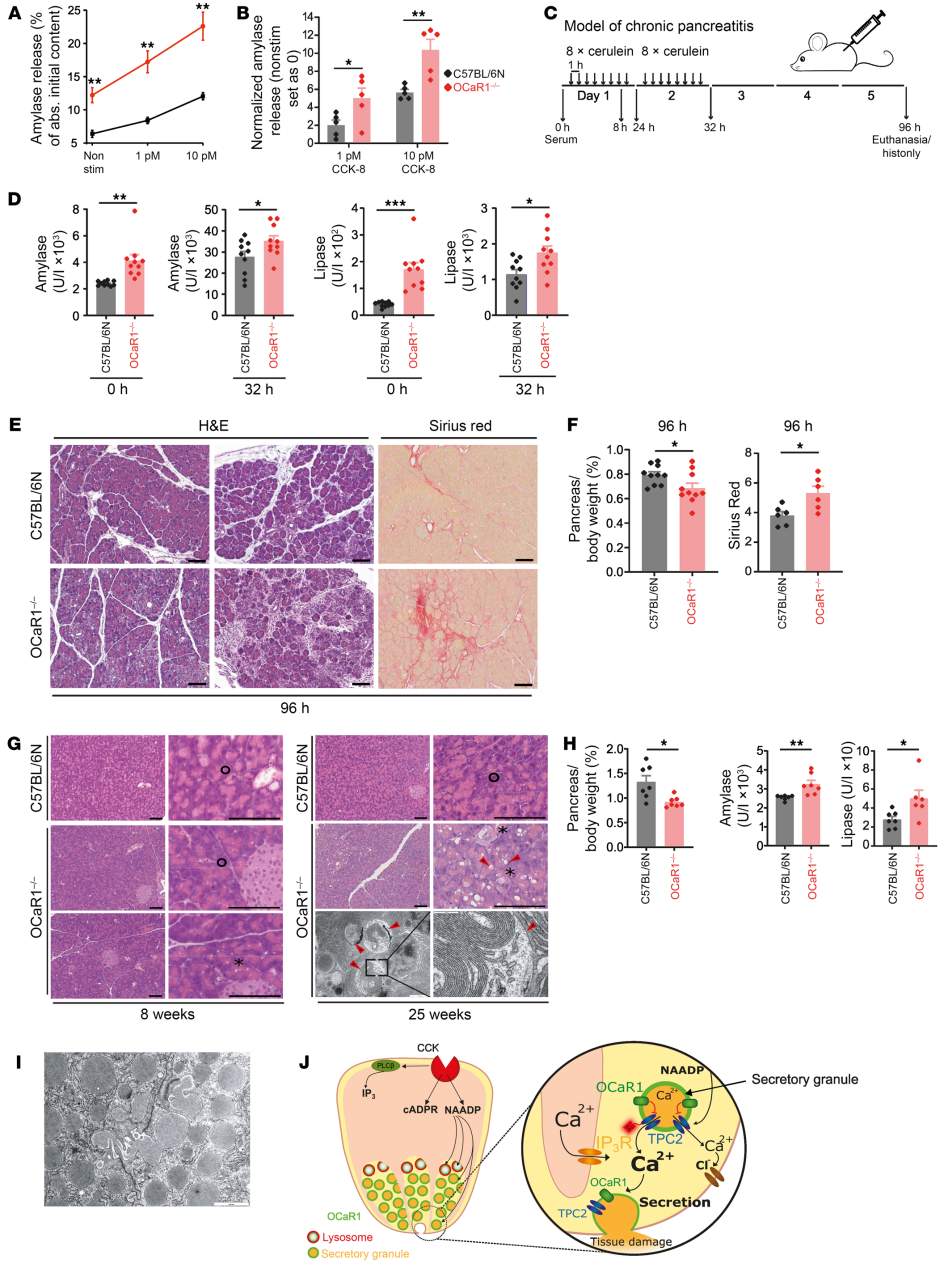
Deletion of *OCaR1* exacerbates severe pancreatitis and disrupts acinar cell homeostasis in older mice. (**A** and **B**) Acini from 5 C57BL/6N and 5 *OCaR1^–/–^* mice were incubated with CCK-8 (0, 1 pM, 10 pM), and absolute amylase release (**A**) as well as its CCK-8–evoked increase (**B**) was determined. (**C**) Scheme of experimental chronic pancreatitis (CP) induction. (**D**) Serum amylase and lipase at 0 and 32 hours of CP induction (*n* = 10 per genotype). (**E**) H&E and sirius red staining at 96 hours of experimental CP (2 slices per mouse, 6 mice per genotype). Scale bars: 50 μm. (**F**) Relative pancreatic weight (percent) (*n* = 10) and fibrosis quantification (sirius red–positive area, *n* = 6) at 96 hours of experimental CP. (**G**) H&E and transmission electron microscopy (TEM) pictures of 8- and 25-week-old *OCaR1^–/–^* and C57BL/6N controls (2 slices per mouse for H&E and >5 slices per mouse for TEM, 3 mice per genotype and time point). Circles, asterisks, and red arrows denote normal acinar cells, acinar cells with vacuoles, and double-membrane autophagosomes, respectively. Scale bars: H&E, 50 μm; TEM, 2,000 nm (left) and 500 nm (right). (**H**) Relative pancreatic weight and serum amylase and lipase of 25-week-old *OCaR1^–/–^* and C57BL/6N mice (*n* = 6, 7). (**I**) Representative TEM picture of zymogen granule fusion observed in 25-week-old *OCaR1^–/–^* mice (>5 slices per mouse, 3 mice). Scale bar: 1,000 nm. (**J**) Model summarizing the action of OCaR1 in acinar cells. Statistical analysis was done by Student’s *t* test (*P < 0.05, **P < 0.01, ***P < 0.001).

In line with the previously observed increase in CCK-8–evoked Ca^2+^ oscillations and amylase secretion under basal conditions, physiological stimulation of CCK receptors also evoked a significantly enhanced amylase release in *OCaR1^–/–^* cells (Figure 5, A and B). Importantly, the concentration-dependent increase in CCK-8–induced exocytosis was preserved in *OCaR1^–/–^* cells, despite the fact that basal exocytosis was markedly higher in *OCaR1^–/–^* cells (WT, 6.4%; *OCaR1^–/–^*, 12.2%), essentially reaching levels similar to those obtained with 10 pM CCK-8 in WT cells (Figure 5, A and B).

Given the enhanced spontaneous and regulated exocytosis in *OCaR1^–/–^* cells, we wanted to investigate the effects of OCaR1 on acinar cell homeostasis and on pancreatic tissue morphology in vivo. To this end, we used models of acute pancreatitis (AP) (Supplemental Figure 16A) and chronic pancreatitis (CP) (Figure 5C). Application of high doses of the CCK analog cerulein in mouse models faithfully recapitulates all stages of pancreatitis (16, 30). To assess the long-term effect of OCaR1 loss on the pancreas and the consequential enhanced spontaneous exocytosis that would occur, we examined aged *OCaR1^–/–^* mice. In line with the increased amylase secretion in *OCaR1^–/–^* acinar cells in vitro (Figure 3A), plasma amylase and lipase levels were significantly elevated in *OCaR1^–/–^* mice under basal conditions (Figure 5D and Supplemental Figure 16, B and C, left) as well as in models of severe AP leading to CP (Figure 5, C and D). In contrast, a similar increase in serum amylase and lipase levels was observed for mild AP in both *OCaR1^+/+^* and littermate *OCaR1^–/–^* mice (Supplemental Figure 16, B and C, right).

Importantly, the increased levels of digestive enzymes noted above aggravated CP pathology in *OCaR1^–/–^* mice compared with WT controls, as evidenced by pancreatic degeneration including acinar cell destruction (H&E staining and pancreas/body weight ratio) that finally resulted in pancreatic weight loss and fibrosis (Figure 5, E and F). Pancreatic tissue damage and indices developed over time in freely living *OCaR1^–/–^* mice during the natural aging process (Figure 5, G–I). While WT mice displayed normal pancreatic morphology at 8 and 25 weeks of age, *OCaR1^–/–^* mice started to exhibit sporadic acinar cell vacuolization at 8 weeks of age, which was more frequent at 25 weeks of age (Figure 5G, asterisks). The observed vacuolization could be attributed to accumulation of double-membrane autophagosomes (Figure 5G, bottom right, red arrowheads). In parallel, the relative pancreatic weight decreased, while serum amylase/lipase levels increased (Figure 5H). Notably, zymogen granule fusion events, an indicator of disturbed cellular homeostasis and cell damage, were detectable in *OCaR1^–/–^* mice (Figure 5I).

In summary, OCaR1 (Figure 5J, green) localizes to secretory granules and lysosomes in acinar cells. Cholecystokinin (CCK) evokes NAADP generation and subsequent Ca^2+^ rise in close vicinity of secretory granules. OCaR1 functionally antagonizes TPC1 and TPC2 channels, thereby limiting Ca^2+^ release (from acidic stores) triggered by activation of the CCK/NAADP signaling pathway, leading to reduced Ca^2+^-dependent exocytosis from secretory granules and, ultimately, preventing pancreatic tissue damage. Proper control of this signaling pathway is crucial in maintaining acinar cell homeostasis during experimental murine models of severe AP, CP, and aging, highlighting its important role in pancreatic physiology and disease.

## Discussion

The central finding of this study is that exocytosis of zymogen granules and hence secretion of digestive enzymes in acinar cells is driven by Ca^2+^ directly released from acidic secretory granules. We show that exocytosis of these vesicles is controlled by OCaR1 operating as a regulator of Ca^2+^ release by inhibiting TPC1 and TPC2 channel function. Although it is not clear how OCaR1 regulates TPC channel function in pancreatic acinar cells, we believe these findings constitute a novel and fundamental mechanism underlying intracellular Ca^2+^ signaling and exocytosis, in which secretory granules have an autoregulatory role in limiting unrestrained exocytosis rather than acting solely as a carrier organelle. Furthermore, OCaR1 in acinar cells critically impacts the disease phenotype in murine models of severe acute and chronic pancreatitis.

### Localization and topology of OCaR1 in endo-lysosomes and secretory vesicles.

We used several complementary strategies to localize native as well as heterologously expressed OCaR1 in acidic intracellular organelles. Imaging experiments in pancreatic acinar cells revealed localization in 2 distinct entities of acidic organelles, in the membrane of secretory granules and endo-lysosomes. Organellar proteomic analysis identified OCaR1 predominantly in secretory/intracellular vesicles as indicated by close cosegregation with several proteins implicated in vesicle endo-/exocytosis. This suggests a dynamic subcellular (re)distribution of OCaR1 during endo-/exocytosis, compatible with its role as regulator of digestive enzyme secretion in acinar cells. Consistent with these findings, OCaR1 (TMEM63A) was previously identified as a component of the lysosomal proteome with unknown function (31, 32), and a liquid chromatography–tandem mass spectrometry (LC-MS/MS) analysis of rat zymogen granules identified OCaR1 as a putative zymogen granule membrane-associated protein (33). OCaR1’s topology revealed by fluorescence protease protection assay, with the N-terminus of OCaR1 inside the organelle lumen, corroborated OCaR1’s location in intracellular vesicles and was congruent with the cryo–electron microscopic structure of OCaR1 (19, 20).

### Molecular function of OCaR1 in secretory granules of acinar cells.

The enhanced spontaneous and regulated secretion of amylase prompted us to investigate Ca^2+^ homeostasis in *OCaR1^–/–^* acinar cells. Although Ca^2+^ release from intracellular organelles and [Ca^2+^]_i_ are tightly controlled in WT cells, spontaneous Ca^2+^ oscillations (in the absence of external Ca^2+^) as well as exaggerated Ca^2+^ oscillations under physiological receptor stimulation were noted upon deletion of OCaR1. Spontaneous Ca^2+^ oscillations were entirely eliminated after disruption of acidic organellar ion homeostasis and Ca^2+^ storage by the V-ATPase blocker bafilomycin A1 or by deletion of TPC1 and TPC2, but not by TPC1 or TPC2 alone. These results indicated that OCaR1 controls Ca^2+^ release from acidic Ca^2+^ stores mediated by TPC channels in acinar cells (and a functional redundancy of TPC1 and TPC2 regarding Ca^2+^ release in OCaR1-containing secretory granules). To elucidate how OCaR1 may counteract TPC1 and TPC2 channel activity, we performed a detailed analysis of current recordings from enlarged endo-lysosomes in COS-7 cells transfected with OCaR1-eYFP alone. No changes in monovalent (Na^+^, K^+^) or divalent (Ca^2+^) cation or Cl^–^ currents were recorded in the presence or absence of PI(3,5)P_2_ (data not shown). In contrast, coexpression of TPC1 or TPC2 (but not TRPML1) showed strong reduction of respective currents by OCaR1.

However, native affinity purifications from solubilized acinar vesicles did not indicate stable biochemical interactions between any of the TPC/OCaR1 subtypes (Supplemental Figure 20). The underlying mechanism must therefore remain open. OCaR1 was previously shown to function as a mechanosensitive channel in the plasma membrane when expressed in HEK293 cells (18, 19, 34), and mechanosensation has a considerable role in the pancreas (35). However, we failed to detect any significant differences in pressure-activated plasma membrane currents between WT and *OCaR1^–/–^* primary acinar cells (i.e., upon positive and negative pressure) in WT (8.3% and 5.9%) as well as in *OCaR1^–/–^* acinar cells (8.6% and 12.5%; Supplemental Figure 19). The unitary conductance of these currents was identical in WT and *OCaR1^–/–^* acinar cells (Supplemental Figure 19, C–E). Thus it must remain open whether OCaR1 could be activated by mechanostimulation within endo-lysosomes and secretory granules of acinar cells, since attempts to measure currents in endo-lysosomes or secretory granules in pancreatic acinar cells or the larger *Drosophila* salivary gland acinar cells have failed so far.

CCK is released into the bloodstream, and its concentration increases from 1 pmol/L to 6–8 pmol/L after ingestion of a typical meal (36). Upon CCK-8 stimulation, global Ca^2+^ oscillations were increased in amplitude and duration in *OCaR1^–/–^* cells and led to enhanced activation of downstream processes. We showed that OCaR1-dependent modulation of CCK-8–evoked Ca^2+^ oscillations and amylase secretion was critical when acinar cells were stimulated with CCK-8 in (sub)picomolar range. Such low agonist-evoked Ca^2+^ oscillations were not affected by the ORAI1/CRAC channel inhibitor GSK-7975A. During Ca^2+^ oscillations evoked by low agonist stimulation, the Ca^2+^ release from the ER is minimal and is replenished by reuptake of the released Ca^2+^ (37) and does not require ORAI1 channels to replenish the ER. However, the ORAI1/CRAC channels had a substantial impact on Ca^2+^ transients evoked by higher CCK-8 concentrations (1 nM) in our study and a previous study (13). Processes downstream of OCaR1-dependent Ca^2+^ oscillations included activation of Ca^2+^-activated Cl^–^ channels in the apical cell pole (38), indicating that OCaR1 controls Ca^2+^ release from stores in the vicinity of the luminal plasma membrane. CCK-evoked global Ca^2+^ signals in pancreatic acinar cells can be triggered by engagement of IP_3_-, cADPR-, and NAADP-sensitive Ca^2+^ pools (39). Whereas cADPR-evoked Ca^2+^ release is mediated by ryanodine receptors (RyRs) in the ER, the NAADP-dependent Ca^2+^ release from acidic stores in acinar cells was recently reported to depend, in part, on TPC2 (10). Indeed, using recordings with Ca^2+^ sensors targeted directly to secretory granules, we showed that enhanced CCK-8 and NAADP-mediated Ca^2+^ release events in *OCaR1^–/–^* acinar cells arise from TPC2-containing compartments most likely through TPC channels (although this does not exclude contributions of other Ca^2+^ release channels). Such Ca^2+^ oscillations were entirely suppressed by the NAADP antagonist Ned-19 and were not “contaminated” by Ca^2+^ release evoked from passive depletion of the ER using thapsigargin or from IP_3_-sensitive Ca^2+^ stores engaged by muscarinic stimulation. When stimulated with carbachol (3, 40), the frequency and amplitude of resulting Ca^2+^ oscillations were rather unchanged in *OCaR1^–/–^* acinar cells compared with WT controls. Only the width of carbachol-induced Ca^2+^ oscillations was increased (Supplemental Figure 17). A likely explanation for this observation is that NAADP-mediated Ca^2+^ release from acidic TPC1- and TPC2-containing granules may sensitize IP_3_ receptors (41). This hypothesis is supported by previous studies in acinar cells showing that local NAADP-induced Ca^2+^ release initiates Ca^2+^-induced Ca^2+^ release through IP_3_ and RyRs, giving rise to global Ca^2+^ signals (9). Also, more recent studies suggest that acidic organelles are in close contact with the ER and that even small Ca^2+^ release events in the nanodomain sensitize IP_3_ receptors to release ER Ca^2+^ (42, 43). To test whether ER Ca^2+^ responses are altered in *OCaR1^–/–^* cells, we depleted the thapsigargin-sensitive stores and observed a significant increase in AUC of *OCaR1^–/–^* cells (Supplemental Figure 18). However, depleting the acidic Ca^2+^ stores using bafilomycin A1 beforehand readjusted ER Ca^2+^ responses to WT levels, suggesting that altered ER Ca^2+^ responses are a consequence of a crosstalk of acidic Ca^2+^ stores with the ER as described previously (44).

### Functional role of OCaR1 in pancreatic physiology and disease.

The release of Ca^2+^ from single isolated pancreatic zymogen granules triggered by IP_3_ and cADPR was shown previously (45). However, to our knowledge our study provides the first direct evidence that the secretory granules have an active role in receptor-evoked and NAADP-mediated Ca^2+^ signaling and may control their own exocytosis. The NAADP pathway and TPC1/2 are present in other secretory cells, including neurons, where they are required for Ca^2+^ signaling and cell function by glutamate receptors (46, 47). It needs further investigation whether OCaR1 has a similar role for Ca^2+^ oscillations and exocytosis in other types of secretory cells.

Elevated basal plasma levels of digestive enzymes in *OCaR1^–/–^* mice could be attributed to a cell-autonomous defect in acinar cells leading to exaggerated zymogen granule release. Aberrant zymogen release is tightly associated with pancreatitis (48). Consequentially, we investigated the development of mild and severe AP in *OCaR1^–/–^* mice (49). Whereas no difference was observed in plasma amylase and lipase concentrations in a model of mild AP, enzyme levels were elevated in *OCaR1^–/–^* mice in a severe AP model. The cause of severe AP and CP is often multifactorial or unknown and usually differs from the straightforward, identifiable mechanism of mild AP initiation, although recurrent mild and severe AP often leads to CP (50–52). CP arises from cumulative injuries to the pancreas over an extended period of time. A diverse array of metabolic, fibrotic, inflammatory, genetic, and environmental risk factors shapes the final pathological picture of CP. Acinar cell resilience to injuries and the interaction of all these risk factors with the cellular homeostasis machinery are crucial during disease development.

Our results pinpoint OCaR1 as a gatekeeper of granular Ca^2+^ release influencing intracellular signaling processes, including exocytosis. Thus OCaR1 should be an important constitutive housekeeper of pancreatic physiology. Notably, this hypothesis is corroborated in older *OCaR1^–/–^* mice, which present with extensive acinar cell vacuolization attributable to an accumulation of autophagosomes and possibly to alterations in autophagy. Autophagy is one of the most important cellular homeostasis mechanisms and has been directly associated with severe AP and CP development and progression (15, 53). Moreover, autophagy regulates zymogen granule and intracellular organelle recycling. Zymogen granule fusion is an indicator of cellular stress–altered recycling mechanisms (54, 55), and can be detected in *OCaR1^–/–^* mice. Consequently, due to the continuous cellular stress, aged *OCaR1^–/–^* mice accumulate tissue injuries that result in elevated plasma digestive enzymes and pancreatic degeneration. This phenotype can be recapitulated in younger mice when severe AP and CP are experimentally induced. Notably, baseline levels of serum amylase and lipase are elevated already before experimental pancreatitis, highlighting the importance of OCaR1 in continuous fine-tuning of acinar cell quiescence. Thus, OCaR1 controls basal homeostatic functions and, consequently, the capacity of acinar cells to counteract stressors over an extended period of time.

## Methods

### Sex as a biological variable.

In this study, we mainly used male mice, as was stipulated in the ethical approval we obtained for this study. The use of only male mice allowed us to identify and understand the intricacies of the studied pathway with minimal confounding sources of variability introduced by hormonal fluctuations and reproductive cycles. When observing male and female mice (Supplemental Figure 7A), we did not detect an influence of sex in the *OCaR1^–/–^* phenotype on the herein measured parameters.

### Generation of OCaR1-knockout and OCaR1–IRES GFP reporter mice.

Detailed cloning strategy and description of the targeting of the genomic DNA for these mouse lines are available in the Supplemental Methods.

### Generation of OCaR1^eYFP/eYFP^ knock-add-on mice.

For construction of the targeting vector (pMWu2_eYFP_final), genomic DNA of R1 embryonic stem (ES) cells was used as a template for PCR amplification of 5′ and 3′ homology arms (together ~5 kb) using *Pfu* polymerase. The 5′ homology contained exons 19–23 of the *OCaR1* gene. The stop codon in exon 23 was replaced by the sequence of the enhanced yellow fluorescence protein (eYFP) followed by *loxP* site–flanked ACN cassette containing an angiotensin-converting enzyme promoter–driven Cre recombinase and a Pol II promoter–driven neomycin resistance gene (NEO). The tACE-Cre contained in the ACE cassette is active in the male germ line and thus enables the self-excision of NEO (56). 3′ of the neomycin resistance cassette, 923 bp of the 3′-untranslated region of the rabbit β-globin gene were inserted to stabilize the transcript of the fusion construct (57, 58). For negative selection, an enhanced GFP cassette and the herpes simplex virus thymidine kinase (tk) cassette were introduced. Gene targeting in hybrid ES cells was performed by inGenious Targeting Laboratory. Correct homologous recombination at the *OCaR1* locus was confirmed by Southern blot hybridization with a 5′ and 3′ probe external to the targeting vector and a neo probe. Germline chimeras were obtained by injection of ES cell clones 2.3B3 and 2.3C3 into C57BL/6N blastocysts. They were crossed with C57BL/6N mice to get mice heterozygous for the *OCaR1^eYFP^* allele (*OCaR1^eYFP/+^*) and were then bred to homozygosity. Mice were routinely genotyped by PCR.

### Generation of transgenic mice expressing Lamp1-RFP or TPC2-GCaMP6m following lentivirus-based transduction.

Lentiviral particles were generated according to previously described protocols (59) following transfection of HEK293 cells (ATTC) with 4 different plasmids using the calcium phosphate method. Lentiviruses were obtained from the supernatant and concentrated. Concentrated lentiviral particles encoding Lamp1-RFP or TPC2-GCaMP6m under control of the PGK promoter were injected into the perivitelline space of zygotes as previously described (60). The copy number of integrants in genomic DNA from pups of zygote transduction was estimated by quantitative PCR. Acinar cells of the adult offspring were analyzed.

### Structural modeling.

Transmembrane topology of OCaR1 (TMEM63A) was predicted with DeepTMHMM (40) and visualized with Protter (61). For prediction of the protein 3-dimensional structure, we used the deep learning–based method AlphaFold (62). The protein is colored according to the predicted local distance difference test (pLDDT) score, which reflects the reliability of prediction, with more reliably predicted regions in blue. The position of the membrane was predicted and visualized using MembraneFold (63).

### Heterologous expression and live-cell imaging in mouse fibroblasts and human embryonic kidney cells.

C-terminal fusions of OCaR1 to YFP or mCherry were generated containing a Kozak sequence upstream of the start codon of the *OCaR1* cDNA sequence (GenBank AAH14795, accession number NM_144794; stop codon was removed) followed by an IIG linker and the eYFP or mCherry sequence. For generation of the *OCaR1-eYFP* construct with N- and C-terminal di-leucine mutants, the following amino acid residues were mutated using the QuikChange Mutagenesis Kit (Stratagene): L191A, L192A, L577A, and I578A. HEK293 cells and mouse embryonic fibroblasts (MEFs) were grown in DMEM (GIBCO) containing 10% FBS. Cells were grown in a 5% CO_2_ humidified incubator at 37°C. Transfection of HEK293 cells with the *OCaR1-eYFP* or *OCaR1-mCherry* constructs (64) was performed using Lipofectamine (Invitrogen). To achieve costaining of acidic organelles, we used LysoTracker Red (Invitrogen; 1 μM, 35 minutes, 37°C). For stable transduction of mouse fibroblasts with calreticulin-dsRed-KDEL, β-1,4-galactosyltransferase–mRFP, or cytochrome *c* oxidase subunit 8A–mRFP, we used lentiviral vectors and lentiviral or adenoviral vectors for *OCaR1-eYFP*. Peroxisomes were labeled using an anti-PMP70 antibody (ab3421, Abcam; 1:1,000). Imaging was performed with a Nikon A1R or Leica SP5 confocal microscope using a Plan Apo λ 60× oil-immersion objective (NA 1.4) or an HCX PL APO CS 63× oil-immersion objective (NA 1.4), respectively. Cells were imaged with a confocal FV1000 Olympus microscope equipped with a UplanSApo 60× oil-immersion objective (NA 1.35; Olympus), and images were processed with Photoshop CS3 (Adobe). Colocalization analysis was performed as described below.

### Colocalization of OCaR1.

Laser scanning microscopy was performed using a spinning disk confocal microscope (ERS-6, Perkin Elmer) or point scanning confocal microscopes (SP5, Leica) equipped with a Plan Apo λ 60× oil-immersion objective (NA 1.4), HCX PL APO CS 63× water-immersion objective (NA 1.2), or HCX PL APO CS 63× oil-immersion objective (NA 1.4), respectively. For colocalization analysis, OCaR1-eYFP was excited at 514 nm and Lamp1-RFP at 561 nm. Image analysis was performed using Fiji or ImageJ (NIH) with the plugin JACoP (65). Pearson’s correlation coefficient (*r*) and Manders coefficients M1 and M2 were used for analyzing colocalization. The background intensity was defined as the average of mean fluorescence intensities of all *z*-planes of a region of interest located outside of the cell and subtracted before further image analysis.

### Fluorescent protease protection assay.

MEFs were transduced with either lentiviral (*PGK-GFP*, *PGK-OCaR1-eYFP*, and *PGK-mCherry-OCaR1*) or adenoviral (*CMCV-Lamp1-GFP*) constructs. Forty-eight hours after transduction, cells were imaged using conventional fluorescent microscopy. To permeabilize cell membrane, 10 μM digitonin was applied after 45 seconds following 50 μg/mL proteinase K after 3 minutes to break down cytosolic fluorescent proteins. Fluorescent intensities of transduced constructs were normalized to intensity before digitonin application.

### Organellar proteomics.

Acinus cells were prepared from freshly isolated mouse pancreas tissue as previously described (66). The cell preparation was homogenized and fractionized by centrifugation, and protein fractions were digested with trypsin and analyzed with mass spectrometry (see Supplemental Methods).

LC-MS/MS data were extracted using msconvert (67). Peak lists were searched against the UniProtKB/Swiss-Prot database (rat, mouse, and human entries, release 2020_05) using Mascot 2.6 (Matrix Science), initially with high peptide mass tolerance (±50 ppm) to enable linear mass shift recalibration (software developed in-house) and then with Δ*m*/*z* set to ±5 ppm in the final search. Fragment mass (MS/MS) tolerance was ±20 mmu; 1 missed trypsin cleavage and the following variable modifications were allowed: acetyl (protein N-terminal), carbamidomethyl (C), Gln→pyro-Glu (N-terminal Q), Glu→pyro-Glu (N-terminal E), oxidation (M), phospho (ST), phospho (Y), propionamide (C). Peptide significance threshold was set to *P* < 0.05. Proteins identified by only 1 specific MS/MS spectrum or representing exogenous contaminations such as keratins or immunoglobulins were eliminated.

Label-free quantification of proteins was based on protein-specific peptide precursor (LC-MS) intensities (peptide-assigned *m*/*z* signal intensities integrated over time) (68) as determined by MaxQuant (69) with integrated effective mass calibration. Features were then aligned between the different LC-MS/MS runs and assigned to peptides with retention time tolerance ± 1 minute and mass tolerance ± 2 ppm using software developed in-house. Relative protein abundance profiles (across the 42 subfractions) were calculated as described by Brechet et al. (70) and visualized using t-distributed stochastic neighbor embedding (t-SNE) with a perplexity of 60. Verified markers for plasma membrane, ER- and Golgi-related compartments, endosomes, lysosomes, peroxisomes, nuclear membrane, and mitochondria (71) were indicated by distinct colors (Figure 2E).

### Determination of basal serum amylase, lipase, and cholecystokinin activity.

Serum amylase and lipase activities were determined using the Amylase and Lipase Liquicolor kits, respectively (Human GmbH). Briefly, approximately 200 μL blood was collected from 10- to 11-week-old male littermate mice by puncture of the buccal vein plexus into heparinized tubes (Sarstedt). Plasma was obtained by centrifugation at 8,000*g* for 15 minutes and used for the determination of amylase and lipase activity, following the manufacturer’s instructions. Plasma cholecystokinin (CCK) levels were determined using an Immunoassay (DRG Instruments GmbH).

### Measurement of amylase release in isolated acini.

Amylase activity was measured with the Amylase kit (Phadebas) following the manufacturer’s instructions. Briefly, acini from 9- to 11-week-old male littermate mice were isolated and aliquoted in tubes and incubated in a shaking water bath at 37°C for 7 minutes without agonist stimulation or in the presence of CCK-8 (1 pM, 10 pM; Sigma-Aldrich). Amylase released to the media was normalized to the total cellular amylase content (determined in the cell pellet after cell permeabilization using 1% Triton X-100). Total protein levels were determined using the Pierce BCA Protein Assay. Vitality of acinar cells was quantified by trypan blue dye exclusion assay.

### Analysis of Ca^2+^ transients.

Single acinar cells and acinar clusters consisting of 2–5 cells were measured with Fura-2 calcium microfluorimetry (see Supplemental Methods). 0.25 mM EGTA and no added Ca^2+^ were used for Ca^2+^-free extracellular conditions. To block Ca^2+^ oscillations, either 50 μM Ned-19 was applied acutely or cells were preincubated with either 100 nM bafilomycin A1 (90 minutes) or 50 μM Ned-19 (30 minutes).

### Measurement of Ca^2+^-activated Cl^–^ currents in acinar cells.

Currents were recorded in classical whole-cell configuration. Patch-clamp pipettes were pulled from glass capillaries (GB 150F-8P, Science Products) using a vertical puller (model PC-10, Narishige) and had a resistance of 3–5 MΩ when filled with the pipette solution. The tip of the pipette was heat-polished. An EPC-10 USB patch-clamp amplifier (HEKA Elektronik), controlled by Patchmaster software (HEKA Elektronik), was used to measure the whole-cell currents. For current recordings in pancreatic acinar cells, single pancreatic acinar cells were freshly dissociated as described above. Whole-cell Ca^2+^-activated Cl^−^ currents were recorded at a holding potential of −60 mV and sampled at 1 kHz. The currents were normalized to the cell capacitance. The bath solution contained 135 mM NaCl, 6 mM KCl, 10 mM HEPES (pH 7.4 with NaOH), 12 mM glucose, 1.2 mM MgCl_2_, and 0.25 mM EGTA. Pipette solution contained 140 mM KCl, 1 mM MgCl_2_, 10 mM HEPES, 5 mM Na_2_ATP, and 0.5 mM EGTA. All experiments were performed at room temperature.

### Lysosomal current recordings.

HEK293 cells (ATTC) for whole–endo-lysosome manual patch-clamp recordings were maintained in DMEM supplemented with 10% FBS, 100 U penicillin/mL, and 100 μg streptomycin/mL at 37°C and 5% CO_2_. Cells were plated on 0.1% poly-L-lysine–coated glass coverslips 60–96 hours before experimentation, and transiently cotransfected using Turbofect (Thermo Fisher Scientific) the next day with plasmids for *mTPC2-RFP* + *OCaR1-YFP* or *mTPC2-RFP* + *VAMP7-YFP*, or *mTPC2-RFP* + an empty *YFP*-vector. The transfected cells were treated with vacuolin (1 μM) overnight. Whole–endo-lysosome manual patch-clamp recordings were conducted using an EPC-10 patch-clamp amplifier (HEKA Elektronik) and PatchMaster acquisition software (HEKA Elektronik). Data were digitized at 40 kHz and filtered at 2.8 kHz. Fast and slow capacitive transients were canceled by the compensation circuit of the EPC-10 amplifier. Recording glass pipettes were polished and had a resistance of 4–8 MΩ. Cytoplasmic solution contained 140 mM K-methanesulfonate (K-MSA), 5 mM KOH, 4 mM NaCl, 0.39 mM CaCl_2_, 1 mM EGTA, and 10 mM HEPES (pH was adjusted with KOH to 7.2). Luminal solution contained 140 mM Na-MSA, 5 mM K-MSA, 2 mM Ca-MSA, 1 mM CaCl_2_, 10 mM HEPES, and 10 mM 2-morpholinoethanesulphonic acid (MES) (pH was adjusted with NaOH to 4.6). In all experiments, 500-millisecond voltage ramps from –100 to +100 mV were applied every 5 seconds, holding potential at –60 mV. The current amplitudes at –100 mV were extracted from individual ramp current recordings. The statistical analyses were performed using Origin 9 software (OriginLab). TPC2-A1-N and TPC2-A1-P were purchased from MedChemExpress.

### Measurement of Ca^2+^ release from TPC2 channel–containing intracellular granules.

The coding part of the pGCaMP6m-N3-TPC2 plasmid (Addgene 80147) (72) was amplified using Q5 High Fidelity DNA Polymerase (New England Biolabs) with primers 5′-ctagtctagaCTCAGATCTCGAGCTCAAGCTTAT-3′ and 5′-tactgatatCGCTCACTTCGCTGTCATC-3′ and inserted in the lentiviral backbone (under the control of the PGK promoter) using restriction sites XbaI and EcorV. Sequencing confirmed the correct amplification. Lentiviral particles were generated according to a previously described methodology (73). Virus physical titer was 1.32 × 10^11^ VP/mL (detected with ELISA). Concentrated lentiviral particles were injected into the perivitelline space of zygotes from *OCaR1^+/+^* and *OCaR1^–/–^* mice as previously described (60). The copy number of integrants in genomic DNA from pups of zygote transduction was estimated by quantitative PCR. Tail biopsies (5–8 mm long) were lysed, and then genomic DNA was purified using QIAGEN QIAamp DNA Micro Kit (order 56304) and eluted in 30 μL of AE Buffer (QIAGEN). Typically, mice with 1.5–4 integrants per genomic DNA copy were used for the experiments.

For the measurements of subcellular local Ca^2+^ rise at the cytoplasmic side of TPC2 channel–containing intracellular granules, we used a setup with an inverted microscope Axio Observer A.1 (Zeiss) equipped with an Objective Fluar 40×/1.3 Oil (Zeiss) and an EGFP Filter (AHF Analysentechnik). Cells expressing the TPC2-GCaMP6m Ca^2+^ sensor were excited using Polychrom V (TILL Photonics) with 490 nm light. The exposure time was 100 milliseconds, and the images were acquired every 5 seconds using a CMOS Prime 95B camera (Photometrics). The experiments were recorded using Zen 2.6 (Zeiss) software. The cells were imaged in an RC41/LP imaging chamber (Warner Instruments) at room temperature. The data are reported as (*F*/*F*_0_), i.e., a change in GCaMP6m fluorescence (*F*) over basal fluorescence (*F*_0_) under low external Ca^2+^ (nominally free Ca^2+^ plus 0.25 mM EGTA).

### Induction of chronic pancreatitis.

Chronic pancreatitis was induced in 10 male, 8- to 11-week-old *OCaR1^–/–^* mice and 10 age-, sex-, and background-matched C57BL/6N controls according to the protocol of Jensen et al. using the CCK analog cerulein (74); at day 5, organ samples were taken for histological analysis (see Supplemental Methods).

### Statistics.

All values are given as mean ± SEM. Statistical significance was tested using the 2-tailed Student’s *t* test paired and unpaired, the χ^2^ test, and 1-way or 2-way ANOVA test with Bonferroni’s or Tukey’s post hoc test (*P ≤ 0.05; **P < 0.01; ***P < 0.001; NS, not significant: *P* > 0.05) using Microsoft Excel, GraphPad Prism 8, or OriginPro (OriginLab).

### Study approval.

All animal procedures were conducted in accordance with the local regulations and approved by the relevant institutional ethical review committees of Saarland University, Heidelberg University, and Technische Universität München (Kreispolizeibehörde Saarpfalz-Kreis, Regierungspräsidium Karlsruhe, Regierung von Oberbayern, Veterinärwesen).

### Data availability.

The RNA sequencing data that support the findings of this study were deposited in the NCBI’s Sequence Read Archive (SRA BioProject PRJNA795174). All other data supporting the findings of this study are available from the corresponding author upon reasonable request. The values underlying mean values in all graphs and behind any reported means where individual data points are not represented are reported in the Supporting Data Values file. Supplemental Table 3 indicates the source, clone number, and description reference of the antibodies used, as well as the sources of mice and cell lines.

## Author contributions

MF, UK, AS, VT, and S Muallem conceptualized and designed the study. UK, AS, VT, S Mannebach, AJ, AW, CG, UW, KND, ML, PW, DV, DS, JECL, IM, CD, M Biel, CWS, PL, VF, S Muallem, WB, US, and MF devised methodology. UK, AS, VT, AJ, AW, KND, ML, PW, DV, OK, CE, JB, DJ, IF, FJT, KP, VK, HZ, DS, JECL, IM, CD, M Berlin, CWS, PL, VF, US, S Muallem, and MF performed investigation and analysis. CG, UW, SH, M Biel, HA, CWS, VF, and NK provided resources. MF, UK, RO, VT, S Muallem, and US wrote the original draft of the manuscript. All authors edited the manuscript. S Mannebach, CG, SGL, CWS, S Muallem, JECL, and MF supervised the study. CG, PW, M Biel, CWS, PL, VF, US, BF, S Muallem, MF, and NK acquired funding. VT, RO, UK, AS, and KND share first authorship in the given sequence for their specific contributions based on relative workload and significance to the project.

## Supplementary Material

Supplemental data

Unedited blot and gel images

Supporting data values

## Version 1 04/01/2024

Electronic publication

## Version 2 04/18/2024

Corrected affiliation no. 3
