# Supplementary material for: OCaR1 endows exocytic vesicles with autoregulatory competence by preventing uncontrolled Ca2+ release, exocytosis, and pancreatic tissue damage
Source: J Clin Invest. 2024 Apr 1;134(7):e169428. doi: 10.1172/JCI169428 (PMC10977991; doi:10.1172/JCI169428)
Supplement: Supplemental data [file jci-134-169428-s006.pdf]

Supplemental Material for:

OCaR1 endows exocytic vesicles autoregulatory competence preventing uncontrolled Ca^{2+} release, exocytosis, and pancreatic tissue damage

Volodymyr Tsvilovskyy[1,2]*, Roger Ottenheijm[1,2]*, Ulrich Kriebs[1]*, Aline Schütz[1]*, Kalliope Nina Diakopoulos[3]*, Archana Jha[4], Wolfgang Bildl[5], Angela Wirth[1,2], Julia Böck[6], Dawid Jaślan[6], Irene Ferro[6], Francisco J. Taberner[1,7], Olga Kalinina[8], Staffan Hildebrand[9], Ulrich Wissenbach[10], Petra Weissgerber[10], Dominik Vogt[1], Carola Eberhagen[11], Stefanie Mannebach[10], Michael Berlin[1,2], Vladimir Kuryshev[1], Dagmar Schumacher[1], Koenraad Philippaert[1,2], Juan E. Camacho-Londoño[1], Ilka Mathar[1], Christoph Dieterich[12], Norbert Klugbauer[13], Martin Biel[14], Christian Wahl-Schott[15], Peter Lipp[16], Veit Flockerzi[10], Hans Zischka[11,17], Hana Algül[3], Stefan G. Lechner[1], Marina Lesina[3], Christian Grimm[6,18], Bernd Fakler[5], Uwe Schulte[5], Shmuel Muallem[4], Marc Freichel[1]

* shared authorship and should be considered first co-authors

[1] Institute of Pharmacology, Heidelberg University, Heidelberg, Germany
[2] DZHK (German Centre for Cardiovascular Research), partner site Heidelberg/Mannheim, Heidelberg, Germany
[3] Comprehensive Cancer Center München, Klinikum rechts der Isar, Technische Universität München, Ismaninger Str. 22, 81675 München, Germany
[4] Epithelial Signaling and Transport Section, National Institute of Dental and Craniofacial Research, National Institute of Health, Bethesda, USA
[5] Institute for Physiology, University of Freiburg, Hermann-Herder-Str. 7, 79104 Freiburg
[6] Walther-Straub-Institut für Pharmakologie und Toxikologie, Ludwig-Maximilians-Universität (LMU) München, 80336 München, Germany
[7] Instituto de Neurociencias de Alicante, Universidad Miguel Hernández – CSIC, 03550 Sant Joan d'Alacant, Spain
[8] Helmholtz Institute for Pharmaceutical Research Saarland (HIPS), Helmholtz Centre for Infection Research (HZI), Saarbrücken, Germany
[9] Institut für Pharmakologie und Toxikologie, Universität Bonn, 53127 Bonn, Germany
[10] Experimental and Clinical Pharmacology and Toxicology, Center for Molecular Signaling (PZMS), Saarland University, Homburg, Germany
[11] Institute of Molecular Toxicology and Pharmacology, Helmholtz Center Munich, German Research Center for Environmental Health, 85764 Neuherberg, Germany.
[12] University Hospital Heidelberg, Department of Medicine III: Cardiology, Angiology and Pneumology, Heidelberg, Germany
[13] Institut für Experimentelle und Klinische Pharmakologie und Toxikologie, Fakultät für Medizin, Albert-Ludwigs-Universität Freiburg, Albertstr. 25, 79104 Freiburg, Germany
[14] Center for Integrated Protein Science (CIPS-M) and Center for Drug Research, Department of Pharmacy, Ludwig-Maximilians-Universität München, and DZHK (German Center for Cardiovascular Research), partner site Munich Heart Alliance, Munich, Germany
[15] Walter Brendel Centre of Experimental Medicine, Biomedical Center, Institute of Cardiovascular Physiology and Pathophysiology, Medical Faculty, Ludwig-Maximilians-Universität München, Planegg-Martinsried, Germany
[16] Institute for Molecular Cell Biology, Centre for Molecular Signalling (PZMS), Universität des Saarlandes, Homburg, Germany
[17] Institute of Toxicology and Environmental Hygiene, Technical University Munich, School of Medicine, 80802 Munich, Germany.
[18] Immunology, Infection and Pandemic Research IIP, Fraunhofer Institute for Translational Medicine and Pharmacology ITMP, 80799 München, Germany

Supplemental Methods

Cloning of *OCaR1* cDNA

Expressed Sequence Tags (EST) databases were screened for EST clones encoding proteins that showed amino acid sequence homology with proteins of the TRP channel family. EST clones containing cDNA sequences derived from the mouse or human *Tmem63a*, *Tmem63b,* and *Tmem63c* genes were identified. *Tmem63a* cDNA was cloned (accession number NM_144794), and the encoded protein was named OCaR1 based on its functional role (see Results). Accordingly, structural homologues TMEM63b (accession number NM_198167) and TMEM63c (accession number NM_172583) were named OCaR2 and OCaR3, respectively.

Western blot analysis

Dishes with a confluent layer of transfected HEK293 cells were washed twice with phosphate-buffered saline (PBS) (pH 7.4) were collected from the dishes without trypsinization after adding 150 µl of 2x Laemmli buffer, sheared through a 0.9 mm diameter cannula, and incubated at 37°C for 30 min before being resolved via SDS polyacrylamide gel electrophoresis (SDS-PAGE) (7% separating gel, 4% stacking gel). Twenty or forty microliters of these samples were loaded. Gels were blotted and proteins probed with anti-GFP primary antibodies (Roche) and detected with horseradish peroxidase-coupled secondary antibodies and the Western Lightning® Chemiluminescence Reagent Plus (Perkin Elmer). Original scans were saved as TIFF files from a LAS 4000 mini (GE Healthcare) and further processed in Corel Draw.

Expression analysis of OCaR1-encoding transcripts using Northern blotting

Poly-(A)$^+$-RNA (10 µg) from the brain and heart of WT and *OCaR1*$^{-/-}$ mice were used for Northern blot analysis as described (1). Blots were hybridized with randomly labeled cDNA probes comprising the full length OCaR1 cDNA (full length probes) and exons 15, 16, and 17 of the mOCaR1 cDNA (delta exon 15, 16, and 17 probes). Filters were exposed to x-ray film for 18–24 h.

To analyze expression of OCaR1 in freshly isolated pancreatic acinar cells, we prepared RNA using the RNeasy Mini kit (Qiagen) and performed one-step reverse transcription-PCR (RT-PCR; Invitrogen) using 20 ng of total RNA/reaction. The intron-spanning primers UK79 and UK80 (see Supplemental Table 2) were used for amplification of

OCaR1 fragments comprising 397 bp. Total RNA isolated from brain (20 ng) was used as a positive control.

Generation of OCaR1 Knock-out and OCaR1-IRES GFP reporter mice

For the construction of the targeting vector (pUK_17), genomic DNA was isolated from R1 ES cells and used as a template for polymerase chain reaction (PCR) amplification of 5′ and 3′ homology arms with Phusion proof reading polymerase (NEB Biolabs). The 5' homology arm was cloned 5' of a loxP site followed by the genomic sequence containing exons 15, 16, and 17 and another loxP site, an IRES-GFP-cassette and an FRT sequence-flanked PGK promotor-driven neomycin resistance gene cassette (neo), and a third loxP site. The herpes simplex virus thymidine kinase cassette (HSVtk) was introduced 5' of the 5' homology arm for negative selection. The targeting construct was sequenced on both strands and no differences were observed compared to the corresponding genomic sequences of the ES cells. Gene targeting in R1 ES cells was performed as described (2). 29 of 454 double resistant colonies showed homologous recombination at the *OCaR1* locus as confirmed by Southern blot with a 5' probe external to the 5′ homology of the targeting vector. Four of those clones were expanded and genotyped for $OCaR1^{+/L3F2}$ using probes for the 3' homology and for the neomycin resistance gene. Injection of the correctly targeted ES cell clone 12E4 into C57BL/6N blastocysts yielded germ line chimeras. Mice carrying the $OCaR1^{L3F1}$ allele were obtained following in vivo deletion of the neomycin selection cassette from $OCaR1^{L3F2/+}$ mice by mating with FlpeR (3) (shown in Supplemental Figure S4E). This targeted allele is transcribed into a bicistronic messenger RNA. In alternative approach, $OCaR1^{+/L3F2}$ mice were mated with Cre deleter (4) mice resulting in effective Cre-mediated excision of exons 15, 16, and 17 (Supplemental Figure S6, C and F) was detected by Southern blotting in $OCaR1^{+/–}$ and $OCaR1^{-/-}$ mice. The exons encode the putative transmembrane segment 5, and deletion generates a premature stop codon in exon 18. Specific 3.3-kb *OCaR1* transcripts expressed in brain and heart from WT mice (Supplemental Figure S6G) were not detectable in brain and heart from $OCaR1^{-/-}$ mice confirming effective deletion of the OCaR1 gene. $OCaR1^{-/-}$ mice on C57BL/6N background (backcrossed for 7 generations) were compared with littermates (indicated as $OCaR1^{+/+}$) or C57BL/6N mice (Charles River). $OCaR1^{-/-}$ mice were crossed with mice lacking TRPML1 (see supplemental methods) or TPC1 (5, 6) and/or TPC2 (7), respectively, to generate $OCaR1^{-/-}$, $TRPML1^{-/-}$ double KO mice,

OCaR1$^{-/-,}$ *TPC1*$^{-/-}$ double KO mice, *OCaR1*$^{-/-}$ *TPC2*$^{-/-}$ double KO mice and *OCaR1*$^{-/-}$ *TPC1*$^{-/-}$ *TPC2*$^{-/-}$ triple KO mice. Mice were genotyped by PCR. Mice were housed in an animal facility with 12-h light/dark cycle (light on at 7:00 A.M.) and food and water was supplied ad libidum.

Generation of *Trpml1* Knock out mice

Mice with a *Trpml1* null allele were generated by flanking exons 2-5 of the *Trpml1* gene with *loxP* sites using gene targeting in FLP C57BL/6 (BF1) ES cells (at inGenious Targeting Laboratory, iTL) to obtain five heterozygous (*Trpml1*$^{+/flox}$) ES cell clones, of which one was used to generate heterozygous mice. Correct targeting, deletion of FRT-flanked NEO cassette as well as of exons 2 to 5 was verified by PCR based genotyping startegies. *Trpml1*$^{+/flox}$ mice were crossed with Cre deleter mice (8) to obtain *Trpml1*$^{+/-}$ and eventually *Trpml1*$^{-/-}$ mice.

Generation of TPC2-GCaMP Knock Add ON mice

Tpc2-GCaMP Knock Add ON mice were generated at inGenious Targeting Laboratory (iTL, New York). To this end *GCaMP6* cDNA was inserted in frame within the last exon 25 of the Tpcn2 gene. The expression of *Tpcn2-GCaMP6* fusion is terminated by the endogenous stop signal. A FRT-flanked Neo selection cassette was inserted downstream of 3'UTR. Targeted iTL HF4 (129/SvEv x C57BL/6 FLP) hybrid embryonic stem cells were microinjected into CD-1 blastocysts. Resulting chimeras with a high percentage agouti coat color were mated to C57BL/6N WT mice to generate germline Neo-deleted mice (*Tpc2-GCaMP*$^{+/T}$). *Tpc2-GCaMP*$^{+/T}$ mice were backcrossed with C57BL/6N WT mice for one more generation and then used for AP-MS analysis.

Confirmation of OCaR1-eYFP fusion transcript expression from the *OCaR1eYFP* allele

MEFs were isolated as described (2), for cloning of cDNA fragments from mice exhibiting the OCaR1^{eYFP} allele. Primers used for amplification of *OCaR1* to *eYFP* transition were as follows: AB87 (5' GGT TGT TCT GCT CAC CAT CC-3'; exon 21, AB90: (5'- GTC CTC CTT GAA GTC GAT GC-3'; eYFP).

Organellar proteomics

Acinus cells were prepared from freshly isolated mouse pancreas tissue as described (9) (with a modification that the cells after the collagenase digestion were resuspended in Ca^{2+}-free solution A supplemented with 0.25 mM EGTA), collected by centrifugation and snap-frozen. 600 mg cell pellets were later homogenized in 8 ml 320 mM mannitol / 10 mM Tris HCl / 0,1 mM $MgCl_2$ pH 7.1 (supplemented with protease inhibitors), first using a glass potter (15 strokes, tight pestle) and mild bath sonication, and then a Cell Cracker homogenizer (HGM/EMBL Heidelberg; 10 passages with a 8,006 mm ball). The homogenate was centrifuged at 1000 x g for 5 min and the supernatant then separated on a 22 ml 0-30% linear Optiprep gradient in homogenization buffer for 1 h at 30,000 rpm (ultracentrifugation in a Beckmann SW 32 Ti rotor). 26 fractions of 1 ml were harvested with a peristaltic pump, each diluted in 3.8 ml lysis buffer (10 mM mannitol, 10 mM Tris HCl pH 7.4, 1 mM EGTA/EDTA, supplemented with protease inhibitors) and incubated for 15 min followed by brief bath sonication. Lysates were then subjected to fractional sedimentation by ultracentrifugation at increasing velocity and run time. Visible membrane pellets, as well as TCA-DOC protein precipitates of three fractions above the top of the gradient were collected, dissolved in Laemmli buffer and shortly run on SDS-PAGE gels (42 samples total). After visualization by silver staining, upper and lower halves of each lane were excised and in-gel digested with trypsin.

Mass spectrometric measurements of tryptic peptide mixtures were carried out on a QExactive mass spectrometer coupled to an UltiMate 3000 RSLCnano HPLC system with autosampler and precolumn (C18 PepMap 100 (300 µm x 5 mm, 5 µm beads); all Thermo Fisher). Peptide samples were dissolved in 50 µl 0.5% trifluoroacetic acid (TFA) and 0.75 µl injected (with 10 µl 0.05% TFA / 4 min). Nano-liquid chromatography (LC) separation: aqueous organic gradient (eluent ‘A’: 0.5% acetic acid; eluent ‘B’ 0.5% acetic acid in 80% acetonitrile; gradient: 1 min 3% B, 120 min from 3% B to 30% B, 20 min from 30% B to 40% B, 10 min from 40% B to 50% B, 5 min from 50% B to 99% B), 5 min 99% B, 5 min from 99% B to 3% B, 10 min 3% B) via a self-packed emitter (i.d. 75 µm; tip = 8 µm, C18/3 µm column material, 23 cm); injection: electrospray at 2.3 kV (positive ion mode), transfer capillary heated to 300 °C. MS instrument settings were: full MS (precursor spectra) resolution 240,000, AGC target 3,000,000, maximum injection time 200 ms, scan range 370 to 1,700 m/z; dd-MS2 (fragment spectra) resolution 15,000, AGC target 100,000, maximum injection time 100 ms, loop count 25 (Top25), isolation window 1.0 m/z, fixed first mass 100 m/z, normalized collision energy

28, minimum AGC target 4,000 (intensity threshold 40,000), charge exclusion unassigned, 1, and >4, peptide match preferred, isotope exclusion on, dynamic exclusion 60 s.

Glucose tolerance test

For glucose tolerance tests (GTTs), mice were fasted overnight (16 h) before they received an intraperitoneal (i.p.) injection of 2 g/kg body weight glucose (B. Braun).The blood glucose concentrations were determined 0, 15, 30, 60, and 120 min after glucose administration.

Laser-capture microdissection

A PALM laser microdissection system with autocatapult and Robocut Software (Zeiss, Oberkochen, Germany) was used for microdissection as described (10). Eight- to nine-µm serial sections from mouse pancreas were transferred to PALM membrane slides (Zeiss), fixed with ethanol and stained with cresyl violet. Stained slices were stored in ethanol at 4°C until microdissection (30 minutes to 4 hours). Fifteen minutes before microdissection, individual slides were air-dried at 21°C. Pancreatic acinar cell clusters consisting of 3–10 cells were microdissected and catapulted into the sterile adhesive caps of 0.5 ml tubes (Zeiss). About 40 chippings of ~1,500 to 7,500 μm^2 were collected per preparation. For RNA isolation, chippings were dissolved in Buffer RLT (Qiagen, Hilden, Germany) after microdissection and snap-frozen on dry ice. Total RNA was extracted from individual caps using the Qiagen RNeasy micro kit with deoxyribonuclease (DNase) on-column digestion. Fifty percent of the eluted RNA was used for one-step reverse transcription-PCR (RT-PCR; Thermo Fisher Scientific). The intron-spanning primers UK79 and UK81 were used for amplification of a specific OCaR1 fragment comprising 397 bp. All oligodeoxynucleotide primers used in this study are listed in Suppl. Table 2. Total RNA isolated from brain (20 ng) was used as a positive control, and H_2O was used as the negative control.

RT-PCR expression analysis

For RT-PCR analysis, RNA isolation and expression analysis in mouse cardiomyocytes, mouse cardiac fibroblasts, and mouse platelets were performed as already described (11, 12). Mouse peritoneal mast cells and bone marrow-derived

mast cells were prepared as described previously (13, 14). RT-PCR was performed as with RNA samples obtained from laser capture microdissection.

Induction of chronic pancreatitis

For the induction of chronic pancreatitis, mice received for two consecutive days (d1, d2) eight hourly, intraperitoneal (i.p.) injections of 0.1 µg cerulein/g body weight (4030451; Bachem Holding AG, Bubendorf, Switzerland) diluted in phosphate buffered saline according to the scheme in Figure 5C. Blood was taken at 0h, 8h, 24h, 32h, and at sacrifice 96h (d5) after the first injection to quantify damage-induced pancreatic enzyme release. Serum was diluted 1/10 with 0.9% NaCl. Amylase activity (AMYL2 Cobas, Roche in Units/l) was quantified by a colorimetric assay according to the IFCC method. Lipase activity (LIPC Cobas, Roche in Units/l) was quantified by DGGR substrate-based assay. After sacrifice, pancreatic tissue was weighted for relative pancreatic weight determination (pancreas weight/body weight depicted in %), fixed with paraformaldehyde, and embedded in paraffin for histological analyses. 2 µm-thick tissue sections were prepared and stained with Hematoxylin & Eosin (H&E) for morphological examination. For detection of fibrosis, deparaffinized and rehydrated slides were counterstained with hematoxylin and incubated for 1 h at room temperature with 0.1% Picro-Sirius red solution (0.1% direct red 80 and 1.3% picric acid in water; Sigma, Munich, Germany). After washing with 0.5% glacial acetic acid in water, slides were dehydrated, cleared in Xylene, and mounted. Pictures (200x magnification, n = 10-15 per mouse) were taken using Axiostar Plus (Carl Zeiss, Göttingen, Germany) and % of Sirius red stained area/picture was quantified with ImageJ. Averages for each mouse were made and used in GraphPad Prism for statistical analysis. Comparisons were made between four non-treated *OCaR1*$^{-/-}$ and four C57BL/6N controls.

Induction of acute pancreatitis (AP)

Blood was collected from overnight starved OCaR1$^{+/+}$ and *OCaR1*$^{-/-}$ mice to analyze basal amylase levels. After a recovery time of 3 days, mice were starved overnight and then administered seven injections (i.p., hourly over 7 hours) of cerulein (50 ng/g body weight) (15). One and 16 hours after the last cerulein injection, blood was collected for the measurement of serum amylase. After the final blood collection, mice were sacrificed and the pancreas was excised in order to determine pancreas dry/wet weight as a measure for edema formation.

Analysis of Ca^{2+} transients

Acinar cells were loaded with Fura-2 by incubation with 4 µM Fura-2 AM (Thermo Fisher Scientific, Darmstadt, Germany) for 30 minutes at room temperature and were perfused (room temperature) with physiological solution (PS, i.e. PSA without BSA and soy bean trypsin inhibitor). $[Ca^{2+}]_i$ was measured following excitation at 340 and 380 nm, and the emitted light was collected by a digital camera with a cutoff filter at 510 nm and analyzed with the Axiovision Software (Zeiss) and Origin Software (OriginLab). $[Ca^{2+}]_i$ is given as the 340/380 ratio and was normalized by dividing the values by the initial basal values at time point 295 s. The frequency of spontaneous and agonist-evoked (e.g 300 fM cholecystokinin or 100 nM carbachol) Ca^{2+} transients were determined by the number of oscillations per cell within 1450 s. The duration and amplitude of these oscillations were analyzed as follows: the exact amplitude and width of each individual Ca^{2+} transient was determined and plotted in a frequency histogram with a binning of 0,1 normalized F340/F380 ratio or 20 seconds for amplitude and width, respectively. The averages of these values were calculated for each individual cell preparation.

The data is represented as normalized (divided by fluorescence ratio at t = 295 s) example traces and summary statistics. Each datapoint on the summary statistics represents one cell isolation, which is one mouse. Statistical testing was performed on mice. 10 µM carbachol (Cch) was applied at the end of the experiment as a positive control for the assay and cell viability, nonresponding cells were excluded from analysis.

Lysosomal current recordings in COS-7 cells

The RFP-TPC1/RFP-TPC2 and mCherry-TRPML1 clones were described elsewhere (16). For recording lysosomal currents in COS-7 cells (ATTC), the pipette (luminal) solution contained (mM) 140 NaCl, 5 KCl, 10 HEPES (pH 7.4 with NaOH), and the bath (cytoplasmic) solution contained 140 NaCl, 5 KCl, 10 HEPES (pH 7.4 with NaOH). Phosphatidylinositol 3,5-bisphosphate ($PI(3,5)P_2$, from Cayman chemicals) was added (1µM) to the bath solution to evoke TPC- and TRPML1-mediated currents. Vacuolin was from Calbiochem, USA. When Na^+, K^+ and Cl^- currents were measured, bath ions were replaced with $NMDG^+$ or $gluconate^-$, as required. COS-7 cells were cultivated in DMEM and 10% fetal bovine serum supplemented with penicillin (100

units/ml)/streptomycin (100 µg/ml), and grown in a water-saturated 5% CO_2, 95% air atmosphere. Cells were transiently transfected with *RFP-TPC1*, *RFP-TPC2* or *mCherry-TRPML1* with and without *YFP*-vector or *YFP-OCaR1* using Lipofectamine 2000 (Thermo Fisher Scientific, Darmstadt, Germany) and were used 24h post-transfection. Lysosomes were liberated mechanically and patched within 1–2 min of release.

Whole lysosomal electrophysiology was performed in isolated endolysosomes using a modified patch-clamp method (17). Transfected COS-7 cells were released and replated on Petri dishes and incubated with culture media for 1 h to allow attachment. Thereafter, the cells were treated with 1 µM vacuolin-1 (a lipid-soluble polycyclic triazine) that selectively increases the size of endosomes and lysosomes. After 1 h treatment with vacuolin-1, enlarged lysosomes were released by quickly pressing patch pipettes against the cell and pulling back. Patch pipettes had a resistance of 6–8 MΩ when filled with the pipette solution. The enlarged lysosomes were identified by monitoring RFP and/or mCherry fluorescence. After formation of a giga-ohm seal, capacitance transients were compensated by Axopatch 200B patch clamp amplifier (Molecular Devices, Sunnyvale, USA). For breaking into the lysosomes, quick voltage steps of +350 mV were applied. The whole lysosomal configuration was confirmed by reappearance of the capacitance transient. The current was recorded by 400 ms rapid alterations of membrane potential (RAMP) from −100 to +100 mV from a holding potential of 0 mV at 4 s intervals. The whole-lysosomal currents were filtered at 1 kHz with an internal four-pole Bessel filter, sampled at 5 kHz, and stored directly to a hard drive by Digidata 1322. Currents were measured with an Axopatch 200B patch clamp amplifier that was controlled by pCLAMP9 software (Molecular Devices, Sunnyvale, USA). The methodology of lysosomal current recordings in HEK293 cells is described in the main methods section.

Single channel recordings of mechanosensitive currents

To examine stretch-activated PIEZO1 currents, PIEZO1 was transfected into N2a-PIEZO1-KO cells (gift from Gary R. Lewin) (18) using the calcium phosphate method. N2a-P1KO cells were grown in a 1:1 mixture of Opti-MEM and DMEM (Thermo Fisher) supplemented with 10% FBS (Thermo Fisher), 2 mM L-glutamine (Thermo Fisher), and penicillin-streptomycin (Thermo Fisher, 100 U/mL) at 37ºC and 5% CO2. On the day before transfection, cells were seeded on poly-L-lysine treated glass coverslips. For transfection, growth medium was replaced with transfection medium consisting of

DMEM, 10% calf serum (Thermo Fisher), and 4 mM L-glutamine. Each coverslip had 0.6 µg DNA diluted in 100 µl water and mixed with 10 µl 2.5 M $CaCl_2$. Subsequently, 100 µl 2x BBS (50 mM HEPES, 280 mM NaCl, 1.5 mM Na_2HPO_4, pH 7.0) was added and vortexed. The resulting DNA mix was added to the transfection medium. After 3–4 hours at 37ºC (5% CO_2), the transfection medium/DNA mix was replaced with regular growth medium. PIEZO1 function was assessed 48 h after transfection.

Stretch-activated currents were recorded as described (19) at a holding potential of -80 mV in the cell-attached patch-clamp configuration (sampled at 20 kHz and filtered at 1 kHz) using an external solution consisting of 140 mM KCl, 1 mM $MgCl_2$, 10 mM glucose, and 10 mM HEPES adjusted to a pH of 7.3 with KOH. The recording pipettes were coated with Sylgard, had a resistance of 6–8 MΩ and were filled with a solution containing 130 mM NaCl, 5 mM KCl, 1 mM $CaCl_2$, 1 mM $MgCl_2$, 10 mM TEA-Cl, and 10 mM HEPES adjusted to pH 7.3 with NaOH. Pressure stimuli were applied with a 1-ml syringe, and the pressure was measured with a custom-made pressure sensor. Single channel amplitudes were determined as the difference between the peaks of Gaussian fits of the trace histograms of 5 consecutive stimuli using Fitmaster software (HEKA Elektronik GmbH).

Transcriptome analysis

RNA was isolated from the pancreas of three 3-week-old male WT (C57BL6/N) and *OCaR1*$^{-/-}$ mice, using TRIZOL reagent (Thermo Fisher Scientific, Germany). The integrity of the RNA samples was verified using a 2100 Bioanalyzer system (Agilent Technologies, USA), and the RNA integrity number (RIN) was between 8.20 and 9.10. A cDNA library was generated using the NEB Next RNA Master Mix Library Prep Kit for Illumina with the NEB Next Oligodeoxynucleotides for Illumina (New England Biolabs, Germany). First-strand synthesis was conducted employing 2.5 µg RNA that was polyA-RNA selected. The read length of the RNA-seq (Hiseq2000 machine using the standard Illumina sequencing by synthesis chemistry) was single-read, 51-base pair reads, SE.

We obtained 30.0–33.9 million reads per sample. All reads were alignesd against the mouse genome sequence (mm10, genome-build GRCm38.p5) using the splice-aware STAR read aligner (version 2.6.1d) (20) with a high-mapping rate (>95%). Among them, 56.6–62.0% were uniquely mapped, and we used them in subsequent analysis. To assess the differential gene expression, only aligned reads covering exons in positive-strand orientation were counted with featureCounts (version 1.6.3) (21).

Differential gene expression analysis was performed with DESeq2 R-package (22). Genes showing counts less than 100 were removed across all samples. A gene was considered differentially expressed if the FDR adjusted p-value was < 0.05.

AP-MS analysis

Acinus cells from mouse pancreas (C57BL/6N, TPC2-GCaMP, OCaR1eYFP/eYFP, 4 mice per genotype) were homogenized with a glass potter (tight pestle) in isotonic homogenization buffer (2 ml/100 mg cells; 320 mM mannitol, 10 mM Tris-HCl pH 7.5, 0.1 mM $MgCl_2$ + 10x protease inhibitors), mildly sonicated (water bath), and passaged 10 times in a Cell Cracker homogenizer (HGM/EMBL Heidelberg; 8.010 mm ball). After centrifugation for 10 min at 5 000 x g the supernatant was collected, the pellet re-homogenized as before but using a hypotonic buffer (10 mM mannitol, 10 mM Tris-HCl pH 7.5, 0.1 mM EGTA/EDTA), and the combined microsomal fractions loaded on a sucrose step gradient (0.5 / 1.4 M in 10 mM Tris-HCl pH 7.5). After centrifugation for 60 min at 180 000 x g (Beckmann SW 32 Ti), vesicles at the sucrose interface were collected, diluted threefold with 20 mM Tris-HCl pH 7.4, pelleted (200 000 x g / 30 min) and resuspended in 20 mM Tris-HCl pH 7.4 (200 µl total).

For each affinity purification (AP), 1.5 mg of membrane protein suspension were solubilized in 1.5 ml CL-47 ComplexioLyte buffer (Logopharm; supplemented with 2 mM EDTA/EGTA, 2 mM iodoacetamide, 1 mM PMSF and 10x protease inhibitor mix (aprotinin, pepstatin, leupeptin; Sigma)). Solubilisates were then incubated for two hours at 4°C with 10 µg magnetic-bead-coupled (Protein A/G, Invitrogen; crosslinked according to the manufacturer's instructions) antibodies: control IgG (rabbit; Upstate Biotechnology), anti-GFP (AF4240, R&D Systems), anti-GFP (A11122, Invitrogen) and anti-TPC1 (#836 + #838, (23)). After two rounds of washing with CL-47 dilution buffer (Logopharm), bound proteins were eluted twice with 6 µl Laemmli buffer without DTT (10 min / 37°C). 100 mM DTT was added to the eluates thereafter and the samples shortly run on an SDS-PAGE prior to tryptic digestion for MS analysis.

MS analysis was carried out on a QExactive MS setup and processed essentially as described for organellar proteomic analysis (in the methods section of the main manuscript and the supplement) with the following modifications: peptide samples were each dissolved in 13 µl 0.5% trifluoroacetic acid each, 5 µl thereof injected; aqueous organic gradient: 6 min 3% B, 60 min from 3% B to 30% B, 10 min from 30% B to 40% B, 5 min from 40% B to 50% B, 5 min from 50% B to 99% B, 5 min 99% B, 5

min from 99% B to 3% B, 10 min 3% B; C18 column: 24.5 cm packed in an equally dimensioned emitter (CoAnn Technology); full MS (precursor spectra): maximum injection time 100 ms; dd-MS2 (fragment spectra): maximum injection time 200 ms, loop count 10 (Top10), minimum AGC target 8,000 (intensity threshold 40,000), dynamic exclusion 30 s. Extracted peak lists were searched against the in-house modified UniProt reference proteome database (UP000000589) + cRAP from The Global Proteome Machine (GPM; http://ftp.thegpm.org/fasta/cRAP/crap.fasta). Label-free quantification of proteins was carried out as described in (24).

Transmission Electron Microscopy

For transmission electron microscopy (TEM) analyses, male, 22-26 week old *OCaR1*$^{-/-}$ (n = 3) and C57BL/6N (n = 3) mice were sacrificed and 1 mm x 1 mm x 1 mm pancreatic tissue pieces were fixed at 4°C with 0.1 M sodium cacodylate buffer (pH 7.4) containing 2.5% glutaraldehyde (16537; Science Services GmbH, Munich, Germany). Subsequently, pancreatic tissue pieces were post-fixed in 1% osmium tetroxide, dehydrated with acetone, and embedded in epoxy resin. Ultrathin sections (50-60 nm) were cut using Ultracut E (Reichert-Jung) and negative-stained with UranyLess (DM22409-20; Science Services GmbH, Munich, Germany) and 3% lead citrate (Leica Biosystems, Nussloch, Germany). Images were acquired using a Jeol 1200 EXII electron microscope (Akishima, Tokyo, Japan) at 60 kV, equipped with a KeenViewII digital camera (Olympus, Hamburg, Germany) and processed with the iTEM software package (anlySISFive; Olympus).

REFERENCES for supplemental material

1. Freichel M, Wissenbach U, Philipp S, and Flockerzi V. Alternative splicing and tissue specific expression of the 5' truncated bCCE 1 variant bCCE 1 D 514 *FEBS Lett.* 1998;422(3):354-8.
2. Freichel M, Suh SH, Pfeifer A, Schweig U, Trost C, Weissgerber P, et al. Lack of an endothelial store-operated Ca2+ current impairs agonist-dependent vasorelaxation in TRP4-/- mice. *Nat Cell Biol.* 2001;3(2):121-7.
3. Farley FW, Soriano P, Steffen LS, and Dymecki SM. Widespread recombinase expression using FLPeR (flipper) mice. *Genesis.* 2000;28(3-4):106-10.
4. Schwenk F, Baron U, and Rajewsky K. A cre-transgenic mouse strain for the ubiquitous deletion of loxP-flanked gene segments including deletion in germ cells. *NucleicAcidsRes.* 1995;23(24):5080-1.
5. Arndt L, Castonguay J, Arlt E, Meyer D, Hassan S, Borth H, et al. NAADP and the two-pore channel protein 1 participate in the acrosome reaction in mammalian spermatozoa. *Mol Biol Cell.* 2014;25(6):948-64.
6. Sakurai Y, Kolokoltsov AA, Chen CC, Tidwell MW, Bauta WE, Klugbauer N, et al. Ebola virus. Two-pore channels control Ebola virus host cell entry and are drug targets for disease treatment. *Science.* 2015;347(6225):995-8.
7. Grimm C, Holdt LM, Chen CC, Hassan S, Muller C, Jors S, et al. High susceptibility to fatty liver disease in two-pore channel 2-deficient mice. *Nat Commun.* 2014;5:4699.
8. Schwenk F, Baron U, and Rajewsky K. A cre-transgenic mouse strain for the ubiquitous deletion of loxP-flanked gene segments including deletion in germ cells. *Nucleic Acids Res.* 1995;23(24):5080-1.
9. Ahuja M, Schwartz DM, Tandon M, Son A, Zeng M, Swaim W, et al. Orai1-Mediated Antimicrobial Secretion from Pancreatic Acini Shapes the Gut Microbiome and Regulates Gut Innate Immunity. *Cell Metab.* 2017;25(3):635-46.
10. Weissgerber P, Kriebs U, Tsvilovskyy V, Olausson J, Kretz O, Stoerger C, et al. Male fertility depends on Ca(2)+ absorption by TRPV6 in epididymal epithelia. *Sci Signal.* 2011;4(171):ra27.
11. Meissner M, Weissgerber P, Londono JE, Prenen J, Link S, Ruppenthal S, et al. Moderate calcium channel dysfunction in adult mice with inducible cardiomyocyte-specific excision of the cacnb2 gene. *J Biol Chem.* 2011;286(18):15875-82.
12. Harper MT, Londono JE, Quick K, Londono JC, Flockerzi V, Philipp SE, et al. Transient receptor potential channels function as a coincidence signal detector mediating phosphatidylserine exposure. *Sci Signal.* 2013;6(281):ra50.
13. Solis-Lopez A, Kriebs U, Marx A, Mannebach S, Liedtke WB, Caterina MJ, et al. Analysis of TRPV channel activation by stimulation of FCepsilonRI and MRGPR receptors in mouse peritoneal mast cells. *PloS one.* 2017;12(2):e0171366.
14. Vennekens R, Olausson J, Meissner M, Bloch W, Mathar I, Philipp SE, et al. Increased IgE-dependent mast cell activation and anaphylactic responses in mice lacking the calcium-activated nonselective cation channel TRPM4. *Nat Immunol.* 2007;8(3):312-20.
15. Lerch MM, and Gorelick FS. Models of acute and chronic pancreatitis. *Gastroenterology.* 2013;144(6):1180-93.
16. Yamaguchi S, Jha A, Li Q, Soyombo AA, Dickinson GD, Churamani D, et al. Transient receptor potential mucolipin 1 (TRPML1) and two-pore channels are functionally independent organellar ion channels. *J Biol Chem.* 2011;286(26):22934-42.
17. Jha A, Ahuja M, Patel S, Brailoiu E, and Muallem S. Convergent regulation of the lysosomal two-pore channel-2 by Mg(2)(+), NAADP, PI(3,5)P(2) and multiple protein kinases. *EMBO J.* 2014;33(5):501-11.
18. Moroni M, Servin-Vences MR, Fleischer R, Sanchez-Carranza O, and Lewin GR. Voltage gating of mechanosensitive PIEZO channels. *Nat Commun.* 2018;9(1):1096.
19. Taberner FJ, Prato V, Schaefer I, Schrenk-Siemens K, Heppenstall PA, and Lechner SG. Structure-guided examination of the mechanogating mechanism of PIEZO2. *Proc Natl Acad Sci U S A.* 2019;116(28):14260-9.

20. Dobin A, Davis CA, Schlesinger F, Drenkow J, Zaleski C, Jha S, et al. STAR: ultrafast universal RNA-seq aligner. *Bioinformatics.* 2013;29(1):15-21.
21. Liao Y, Smyth GK, and Shi W. featureCounts: an efficient general purpose program for assigning sequence reads to genomic features. *Bioinformatics.* 2014;30(7):923-30.
22. Love MI, Huber W, and Anders S. Moderated estimation of fold change and dispersion for RNA-seq data with DESeq2. *Genome Biol.* 2014;15(12):550.
23. Castonguay J, Orth JHC, Muller T, Sleman F, Grimm C, Wahl-Schott C, et al. The two-pore channel TPC1 is required for efficient protein processing through early and recycling endosomes. *Sci Rep.* 2017;7(1):10038.
24. Brechet A, Buchert R, Schwenk J, Boudkkazi S, Zolles G, Siquier-Pernet K, et al. AMPA-receptor specific biogenesis complexes control synaptic transmission and intellectual ability. *Nat Commun.* 2017;8:15910.

Supplemental Figure 1

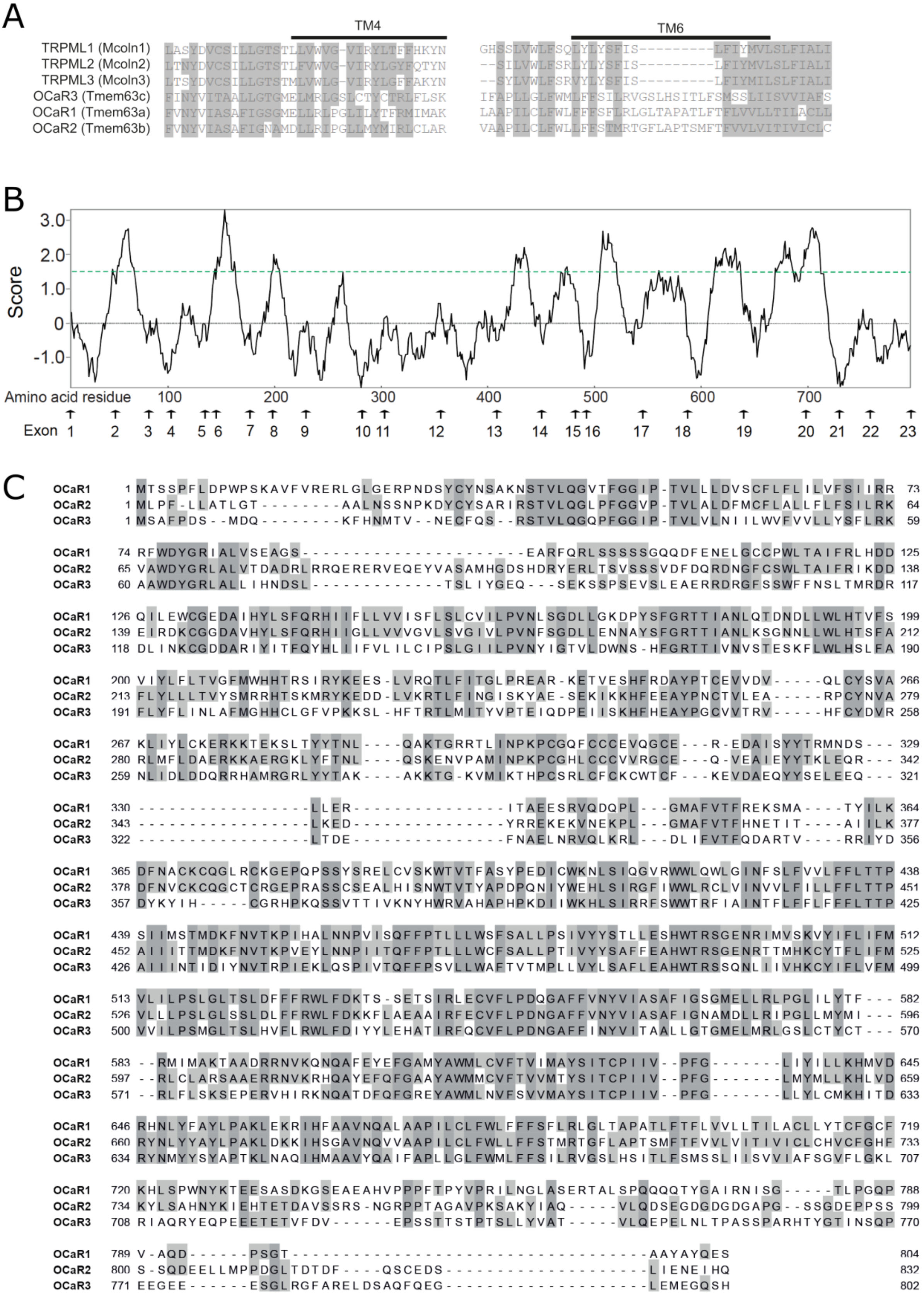

Figure S1 (related to Figure 1): Primary structure, hydrophobicity analysis and sequence alignment of OCaR1

(A) Alignment of the amino acid sequences of putative transmembrane domains 4 and 6 (TM4, TM6) of mouse TRPML1 (also named MCLN1, Mucolipin-1, UniProt Q99J21), TRPML2 (also named MCLN2, Mucolipin-2, UniProt Q8K595), and TRPML3 (also named MCLN3, Mucolipin-3, UniProt Q8R4F0) with the OCaR1 (also named CSC1-like protein 1 (CSCL1) or TMEM63A, UniProt Q91YT8), OCaR2 (also named CSC1-like protein 2 (CSCL2) or TMEM63B, UniProt Q3TWI9), and OCaR3 (also named CSC1 or TMEM63C, UniProt Q8CBX0) proteins. **(B)** Kyte-Doolittle Hydropathy analysis of the OCaR1 gene product. The amino acid sequence was plotted using Accelerys software. On the x-axis, the coding exons and corresponding amino acid residues are shown. Seven to ten peaks reach or pass the dotted line at score 1.5 and extend over >18 amino acid residues indicating hydrophobic stretches possibly representing transmembrane domains. **(C)** Alignment of the amino acid sequences of mouse OCaR1, OCaR2, and OCaR3 proteins. Amino acids that are identical in two or all three OCaR proteins are highlighted.

Supplemental Figure 2

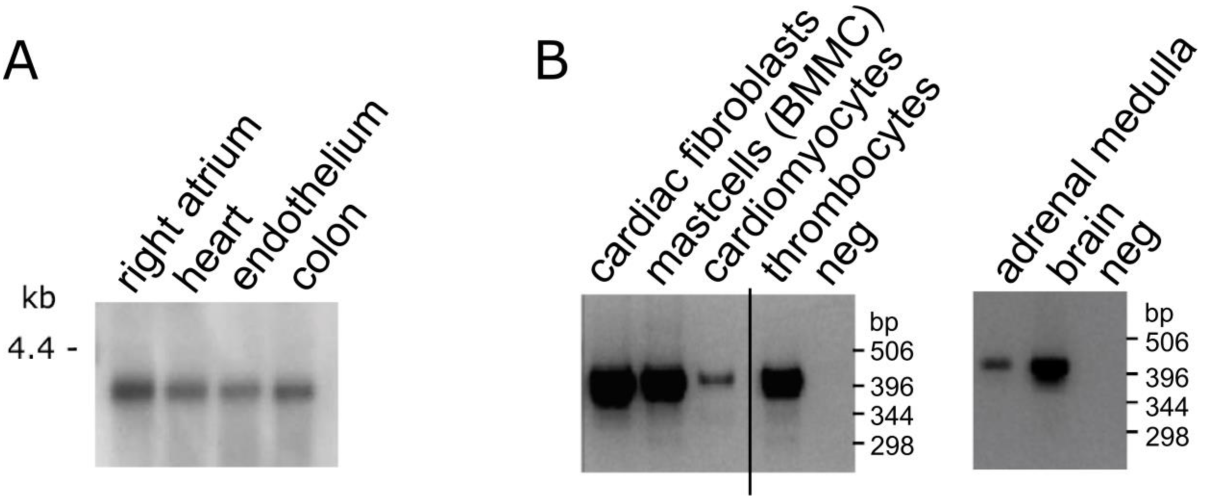

Figure S2 (related to Figure 1): Expression analysis of OCaR1 transcripts in *OCaR1*$^{-/-}$ mice.

(A) Northern blot analysis identifies OCaR1 transcripts (3.3 kb) in poly(A)+ RNA of mouse right atrium, heart, aortic endothelial cells (endothelium), and colon. **(B)** RT-PCR Analysis identifies OCaR1 transcripts (397 bp, primer UK 79, UK 81) in total RNA from cardiac fibroblasts, BMMC (mast cells), cardiomyocytes, thrombocytes, adrenal medulla, and brain. The left part contains noncontiguous lines from the same gel, separated by a black line.

Supplemental Figure 3

A

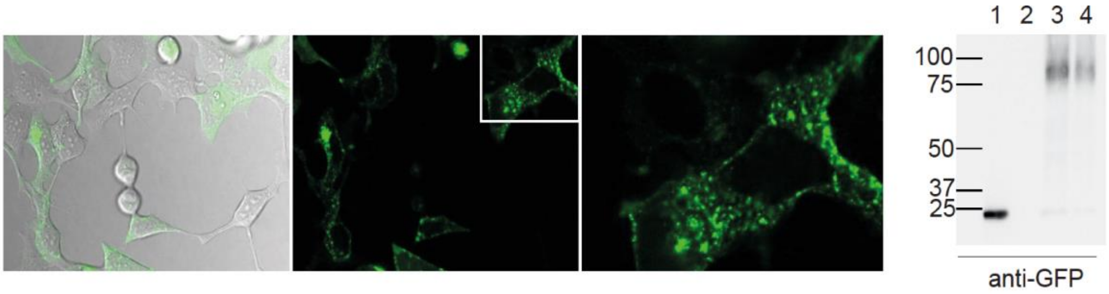

B

Organelle marker	R	M1	M2
Lysotracker	0.765±0.024	0.740±0.060	0.732±0.090
ER	0.189±0.021	0.079±0.014	0.106±0.015
Golgi	0.161±0.017	0.148±0.014	0.09±0.007
Mitochondria	0.085±0.022	0.062±0.015	0.069±0.017
Peroxisomes	0.084±0.041	0.09±0.026	0.094±0.041

C

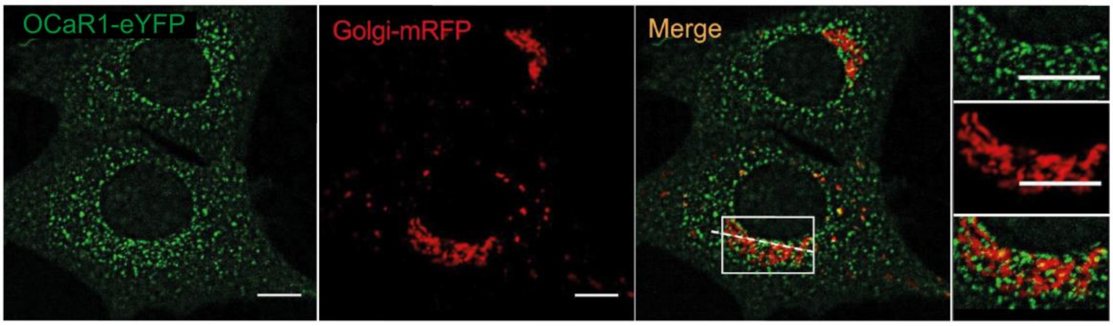

D

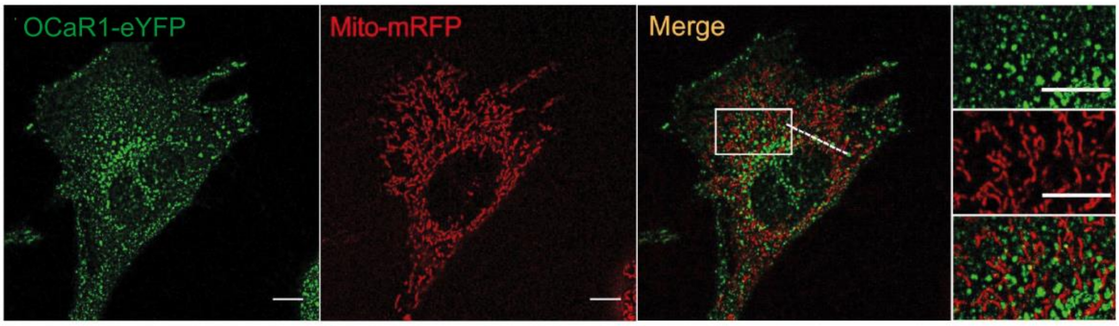

E

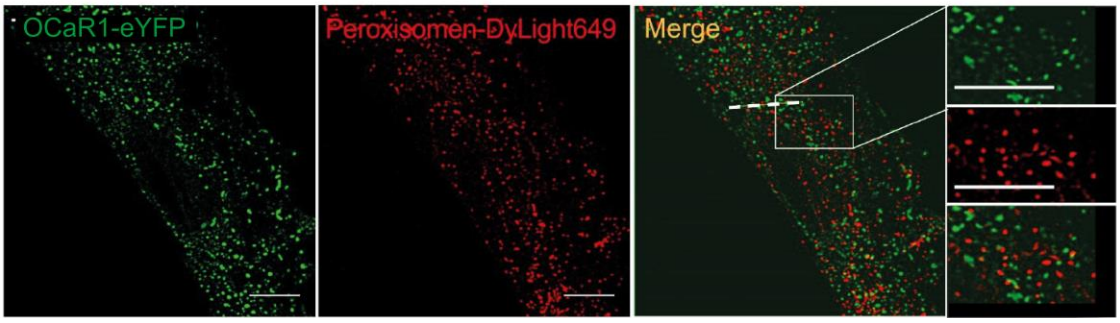

Figure S3 (related to Figure 1): OCaR1 proteins are localized in acidic organelles but not in ER, Golgi, mitochondria, or peroxisomes.

(A) Expression of OCaR1-eYFP transfected into HEK293 cells. Microscopy: merged image (left panel) of DIC image with OCaR1-eYFP fluorescence (middle panel) and a magnification (right panel) of the inset shown in the middle panel. Western Blot: expression analysis using an anti-GFP antibody to detect OCaR1-eYFP fusion proteins expressed in HEK293 cells (lane 3, 40 µl; lane 4, 20 µl of total lysate). eYFP transfection (as control in lane 1), mock transfection (lane 2), and protein size markers are indicated. **(B)** Summary of co-localisation analysis of OCaR1-eYFP with the corresponding organelle markers. Pearson and Manders coefficients were determined from n = 4 (Lysotracker), n = 8 (ER), n = 7 (Golgi apparatus), n = 7 (Mitochondria), and n = 4 (Peroxisomes) cells. **(C-E)** Mouse embryonic fibroblasts (MEFs) expressing OCaR1-eYFP after viral transduction and coexpressing the organelle marker β-1,4-galactosyl-transferase-mRFP (**C,** Golgi apparatus, n = 7), Cyt-C oxidase subunit-8A-mRFP (**D,** mitochondria, n = 7) or stained with anti-PMP70 antibodies (**E**, peroxisomes, n = 4). Scale bars are 10 µm.

Supplemental Figure 4

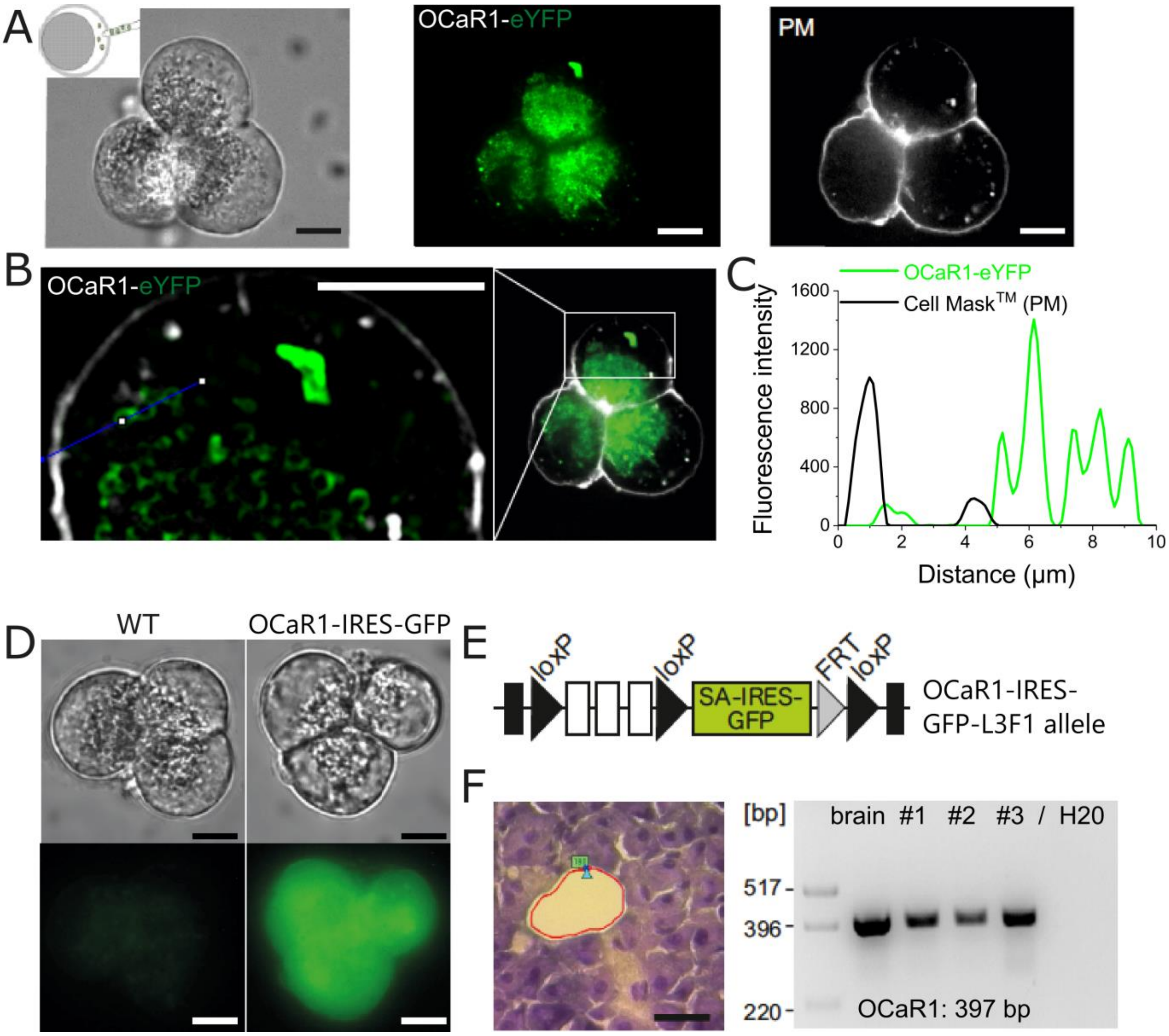

Figure S4 (related to Figure 2): OCaR1 expression in acinar cells and localization in lysosomes and secretory granules at the apical pole of acinar cells

(A) Analysis of subcellular localization of OCaR1-eYFP fusion proteins in pancreatic acinar cells from offspring derived from zygotes transduced via perivitelline injection with a lentiviral vector (inset) expressing OCaR1-eYFP under control of the pgk promoter. OCaR1-eYFP fluorescence is localized at the apical pole of acinar cells (middle panel). Plasma membrane (PM) is stained using Cell Mask^{TM} dye (right panel). No comparable fluorescence was seen in acinar cells derived from WT mice (not shown). Scale bar 10 µm. **(B)** Magnification (left panel) of the inset in the merged image (middle panel) indicates membraneous and vesicular localization of OCaR1-eYFP in acinar cells. Scale bar 10 µm. **(C)** Line-profile of fluorescence intensity values along the dotted line in the merged image shown in B. Representative image from 7 experiments. **(D)** DIC images (upper panels) and GFP fluorescence (lower panels) in acinar cell clusters of WT mice (left) and mice homozygous for the *OCaR1-IRES-GFP* allele (*OCaR1*^{L3F1} allele, right). Scale bar 10 µm. **(E)** In the *OCaR1-IRES-GFP* allele, which originates after removal of the NEO cassette from the *OCaR1*^{L3F2} allele (see Figure S6B), exons 15, 16 and 17 are flanked by loxP sites (filled triangles), followed by a Splice Acceptor (SA)-internal ribosomal entry site (IRES)-GFP cassette to render GFP as expressional reporter under control of the endogenous OCaR1 promoter. **(F)** RT-PCR-analysis of OCaR1 expression in microdissected mouse pancreatic acinar cells. Cresyl violet stain of pancreatic cryosections after laser capture microdissection (LCM; scale bar, 25 µm, left). Amplification of OCaR1-specific transcripts (397 bp) from RNA of microdissected acinar cells (3 independent preparations #1, #2, #3) or brain (positive control) using One step RT-PCR (right).

Supplemental Figure 5

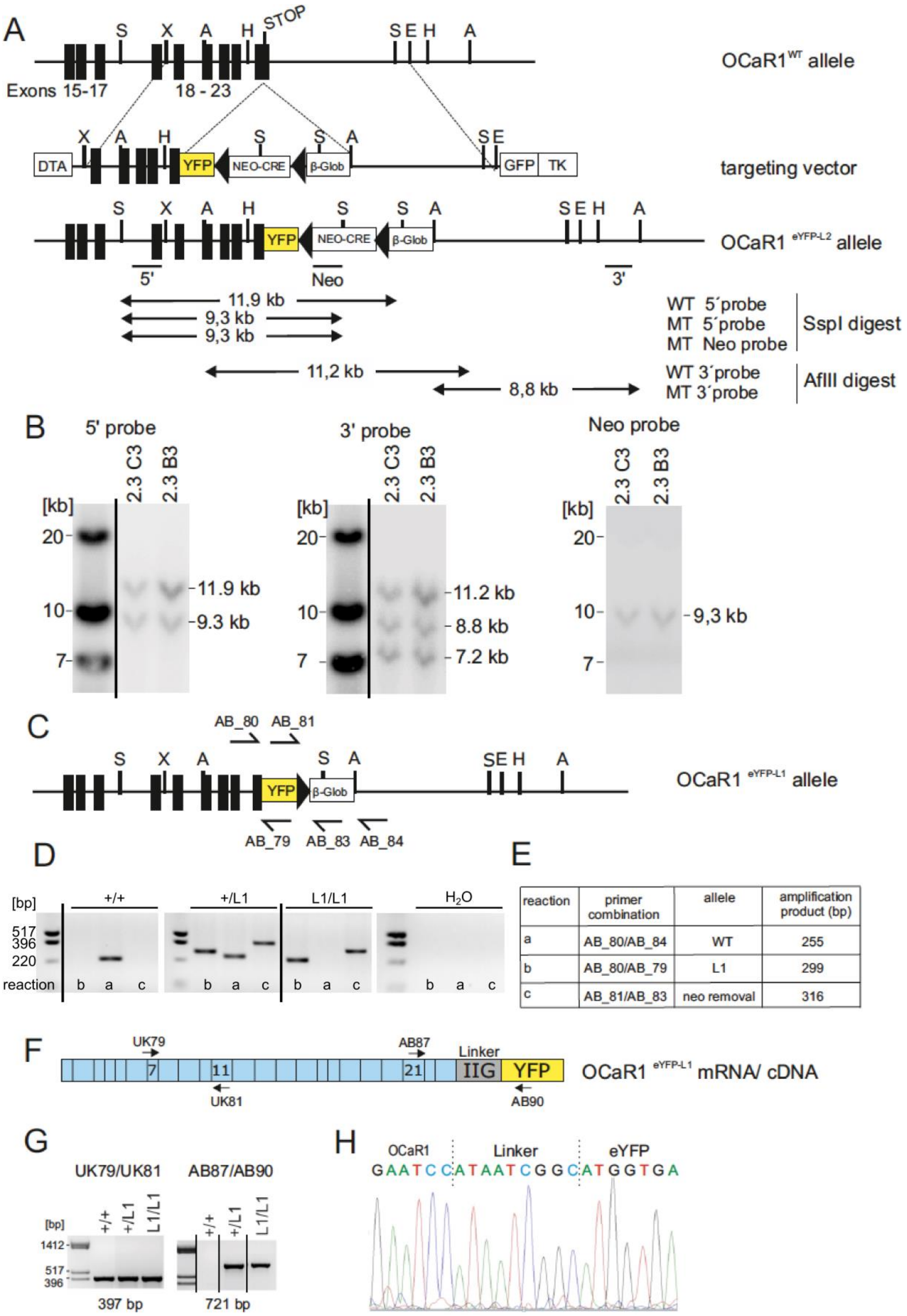

reaction	primer combination	allele	amplification product (bp)
a	AB_80/AB_84	WT	255
b	AB_80/AB_79	L1	299
c	AB_81/AB_83	neo removal	316

Figure S5 (related to Figure 2): Generation of mice carrying an OCaR1-eYFP-Knock-Add-On allele

(A) Conversion of the OCaR1 WT allele (OCaR1^{WT}) into the OCaR1$^{eYFP\text{-}L2}$ allele by gene targeting. In the OCaR1$^{eYFP\text{-}L2}$ allele, the stop codon is replaced by the sequence of eYFP leading to the expression of the OCaR1-eYFP fusion protein under the control of the endogenous OCaR1 promoter. WT OCaR1 allele, targeting construct, and recombinant OCaR1$^{eYFP\text{-}L2}$ allele are shown. In the OCaR1$^{eYFP\text{-}L2}$ allele an ACE-Cre-Neo cassette is flanked by loxP sites leading to its excision after Cre expression in the male germ line and generation of the OCaR1$^{eYFP\text{-}L1}$ allele (illustrated in C). *S, SspI; A, AflII*. Probes and sizes of genomic DNA fragments as expected for Southern blot analysis are indicated. **(B)** Identification of the recombinant OCaR1$^{eYFP\text{-}L2}$ allele in targeted ES cells by Southern blot analysis with 5′ and 3′ probes placed external to the targeted sequence and a neo probe. **(C-E)** Scheme of the OCaR1$^{eYFP\text{-}L1}$ allele following Cre-mediated excision of the ACE-Cre-Neo cassette in the male germline (C) and PCR genotyping (D) of WT mice (+/+) as well as mice heterozygous (+/L1) or homozygous (L1/L1) for the OCaR1$^{eYFP\text{-}L1}$ allele are shown. Localization of corresponding oligonucleotides is indicated in (C), the oligonucleotide combination and expected size of amplicons obtained from corresponding alleles are shown in (E). **(F)** Scheme illustrating OCaR1-eYFP mRNA and localization of primers used for RT-PCR in (G). **(G)** Amplification of OCaR1-specific sequences (left panel) and OCaR1/eYFP transition (right panel) from cDNA derived from RNA isolated from MEFs of WT (+/+), heterozygote OCaR1$^{eYFP\text{-}L1/+}$ (+/L1), and homozygous OCaR1$^{eYFP\text{-}L1/eYFP\text{-}L1}$ (L1/L1) mice; size of amplicons as indicated. **(H)** Sequence analysis of a cDNA fragment amplified with primer pair AB87 and AB90 from homozygous OCaR1$^{eYFP\text{-}L1/eYFP\text{-}L1}$ (L1/L1) mice.

Supplemental Figure 6

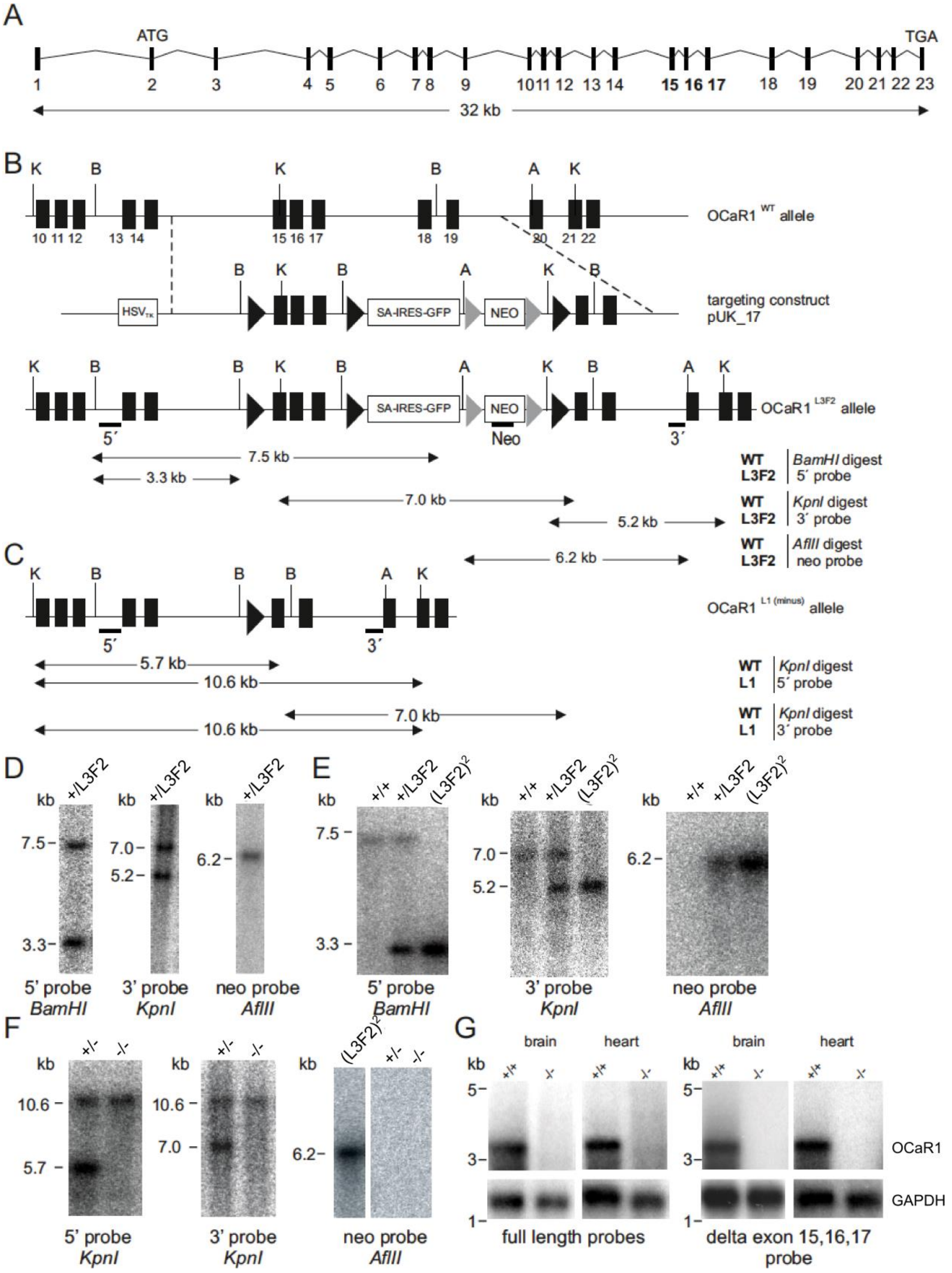

Figure S6 (related to Figure 3-5): Generation of OCaR1-deficient and OCaR1 IRES –GFP reporter mouse lines

(A) Intron–exon distribution of OCaR1 (exons shown as vertical lines) on mouse chromosome 1. The OCaR1 gene extends over 32 kb and exhibits 23 exons, of which 22 exons are coding. The start codon ATG is located in exon 2. The transcript length covers 3,306 bp and the encoded OCaR1 protein comprises 804 residues. **(B-G)** A Cre-loxP based strategy was used to excise exons 15, 16 and 17, thereby inducing a stop codon in exon 18 of the OCaR1 gene. (B) The WT *OCaR1* allele, targeting construct, and recombinant $OCaR1^{L3F2}$ allele. Translated exons (not in scale) are shown as filled boxes. In the $OCaR1^{L3F2}$ allele, exons 15, 16 and 17 are flanked by loxP sites (black triangles); a FRT site (gray triangles)-flanked PGK-neor cassette is located downstream of the third loxP site. A, *AflII*, B, *BamHI*; K, *KpnI*. Probes and sizes of genomic DNA fragments as expected for Southern blot analysis are indicated. HSVtk, herpes simplex virus thymidin kinase. SA-IRES-GFP, splice acceptor- internal ribosomal entry site, followed by the cDNA of the green fluorescent protein. 5', 3' and Neo-probes are indicated as black bars. **(C)** Cre-expression leads to the deletion of exons 15, 16 and 17 and to the conversion of the $OCaR1^{L3F2}$ allele to the $\mathrm{OCaR1}^{\mathrm{L1minus}}$ allele. **(D, E)** Identification of the recombinant $OCaR1^{L3F2}$ allele in recombinant ES cells (**D**) and mice (**E**) by Southern blot analysis using 5' and 3' probes placed externally to the targeted sequence and a neo probe that is directed against the internal PGK-neor cassette. **(F)** Cre expression in mice resulted in the conversion of the 5.7-kb fragment of the $OCaR1^{L3F2}$ allele to a 10.6-kb fragment that is characteristic for the $\mathrm{OCaR1}^{\mathrm{L1minus}}$ allele (5' probe; *KpnI* digest, indicated in **C**). The 3' probe detects the alteration of the 7.0 kb fragment of the $OCaR1^{L3F2}$ allele to a 10.6-kb fragment of the $\mathrm{OCaR1}^{\mathrm{L1minus}}$ allele (*KpnI* digest). In the following, mice carrying the $\mathrm{OCaR1}^{\mathrm{L1minus}}$ allele are labeled as $OCaR1^{+/-}$ and $OCaR1^{-/-}$. Cre activity also leads to the excision of the PGK-neor cassette so that the 6.2-kb neo probe signal detectable for the $OCaR1^{L3F2}$ allele is absent in $OCaR1^{+/-}$ and $OCaR1^{-/-}$ mice (*AflII* digest). **(G)** Northern blot analysis of poly(A) RNA isolated from brain and heart from WT (+/+) and $OCaR1^{-/-}$ (-/-) mice hybridized with three OCaR1-specific probes that cover the full length mRNA (full length probes) and a probe that exhibits the coding sequence of exons 15, 16 and 17 (delta exon 15, 16, 17 probe), respectively. OCaR1 specific 3.3-kb transcripts were identified in WT brain and heart, whereas no signal was detected in brain and heart of *OCaR1-/-* mice. Gapdh was used as a loading control.

Supplemental Figure 7

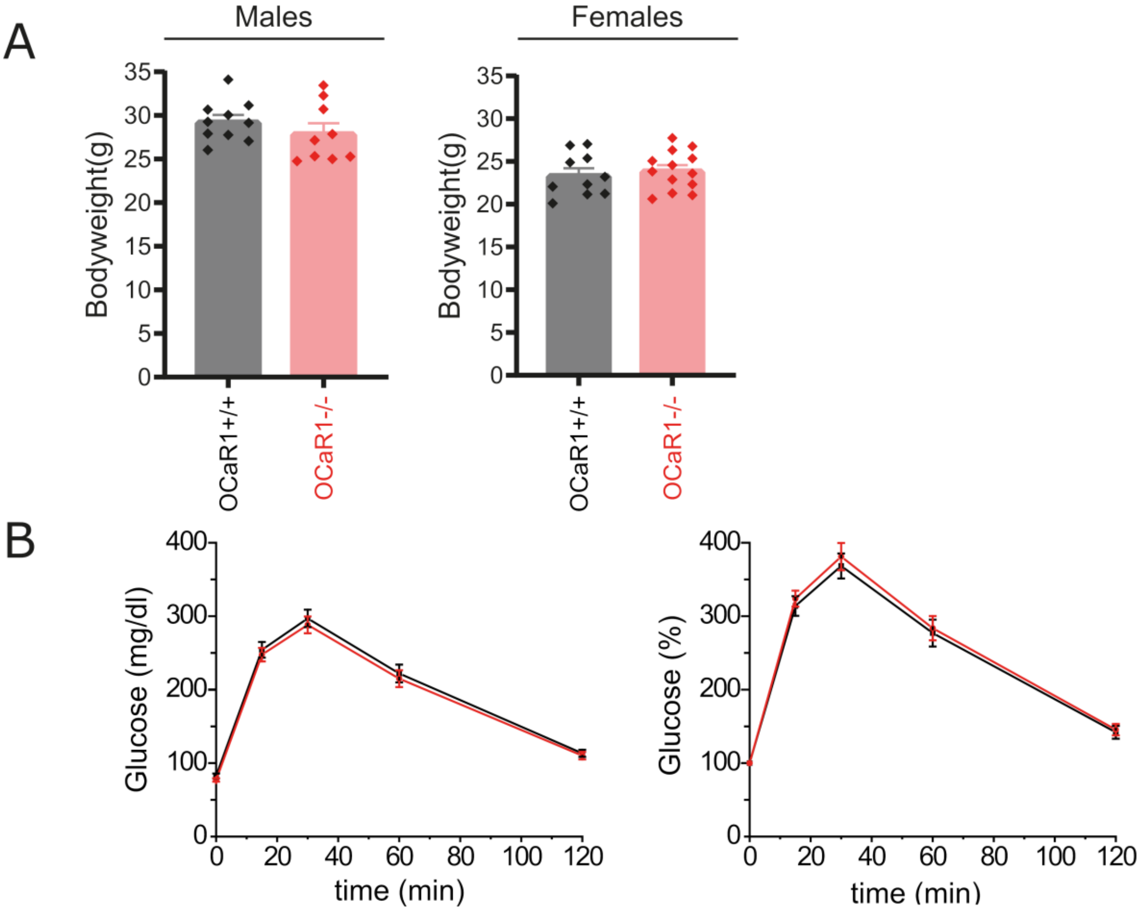

Figure S7 (related to Figure 2): No differences in body weight and glucose levels between *OCaR1*$^{-/-}$ and control mice.

(A) Body weight of 3 to 6 month old male *OCaR1*$^{+/+}$ (n = 10) and *OCaR1*$^{-/-}$ (n = 9) littermates (left panel) and 3 to 5 months old (*OCaR1*$^{+/+}$ n = 10, *OCaR1*$^{-/-}$ n = 13) female littermates (right panel). **(B)** Time course of absolute (left panel) and relative increase (right panel) of blood glucose levels in *OCaR1*$^{+/+}$ (black, n = 22) and *OCaR1*$^{-/-}$ (n = 24, red) age matched (5-6 months in average) male mice upon oral application of glucose (2 g/kg body weight). Analysis by two-tailed Student´s t-test, p>0.05, not significant, not indicated.

Supplemental Figure 8

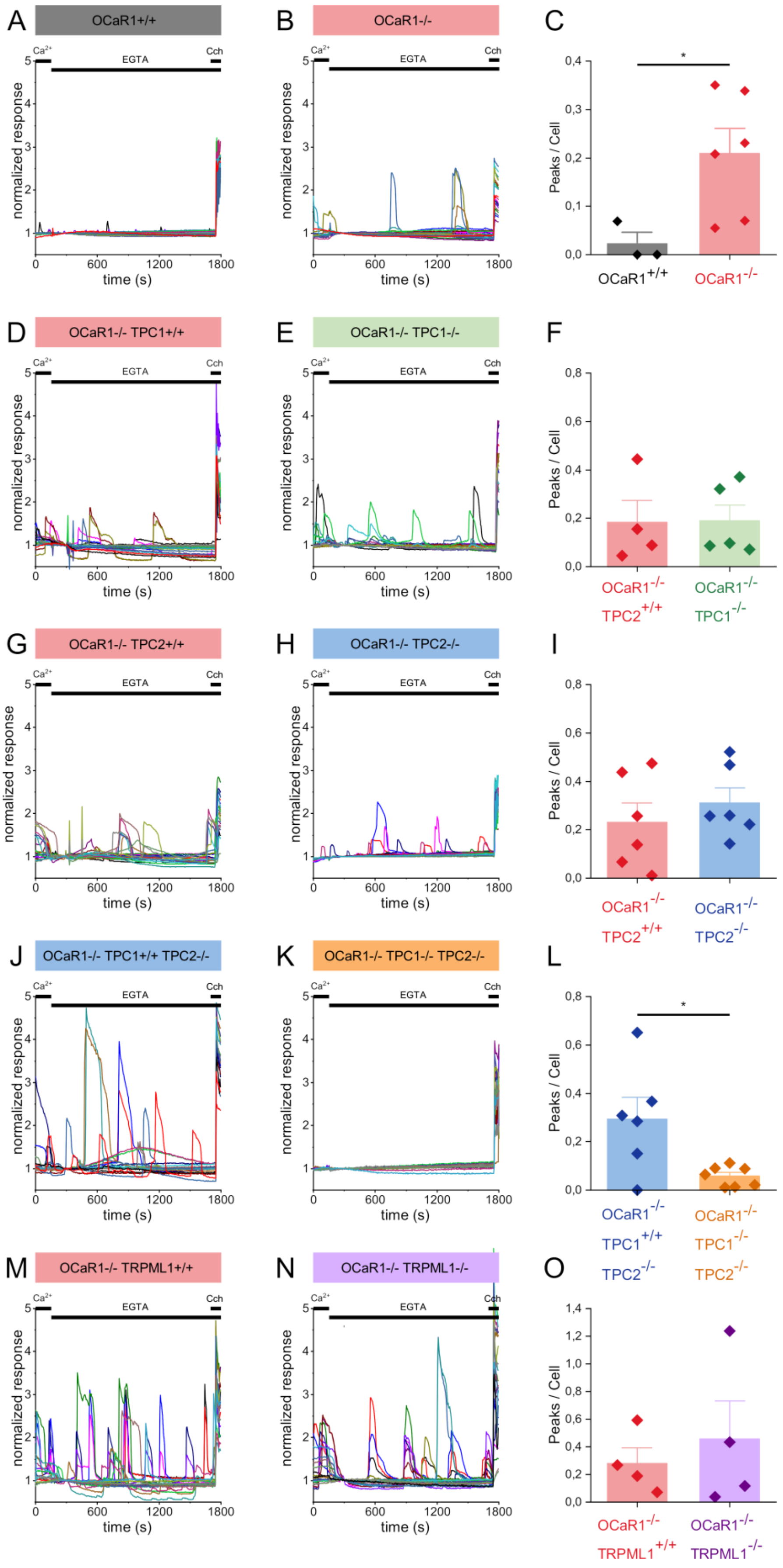

Figure S8 (related to Figure 3): Ca^{2+} release in acinar cells from littermate mice. **(A)** Representative traces (25 cells per genotype) of Fura-2 fluorescence in pancreatic acinar cells of *OCaR1*$^{+/+}$ and **(B)** littermate *OCaR1*$^{-/-}$ mice in the absence of extracellular calcium. **(C)** Peaks per cell of the experiments in (A) and (B) *OCaR1*$^{+/+}$ n = 3 mice and *OCaR1*$^{-/-}$ n = 6 mice. **(D, E)** As (A, B) but with **(D)** *OCaR1*$^{-/-}$ *TPC1*$^{+/+}$ and **(E)** *OCaR1*$^{-/-}$ *TPC1*$^{-/-}$ double knockout littermates. **(F)** Peaks per cell of the experiments in (D) and (E) *OCaR1*$^{-/-}$ *TPC1*$^{+/+}$ n = 4 mice and *OCaR1*$^{-/-}$ *TPC1*$^{-/-}$ n = 5 mice. **(G, H)** As (A, B) but with **(G)** *OCaR1*$^{-/-}$ *TPC2*$^{+/+}$ and **(H)** *OCaR1*$^{-/-}$ *TPC2*$^{-/-}$ littermates. **(I)** Peaks per cell of the experiments in (G) and (H) *OCaR1*$^{-/-}$ *TPC2*$^{+/+}$ n = 6 mice and *OCaR1*$^{-/-}$ *TPC2*$^{-/-}$ n = 6 mice. **(J,K)** As (A, B) but with **(J)** *OCaR1*$^{-/-}$ *TPC1*$^{+/+}$ *TPC2*$^{-/-}$ and **(K)** *OCaR1*$^{-/-}$ *TPC1*$^{-/-}$ *TPC2*$^{-/-}$ triple knockout littermates. **(L)** Peaks per cell of the experiments in (J) and (K) *OCaR1*$^{-/-}$ *TPC1*$^{+/+}$ *TPC2*$^{-/-}$ n = 5 mice and *OCaR1*$^{-/-}$ *TPC1*$^{-/-}$ *TPC2*$^{-/-}$ n = 7 mice. **(M,N)** as (A,B) but with **(M)** *OCaR1*$^{-/-}$ *TRPML1*$^{+/+}$ and **(N)** *OCaR1*$^{-/-}$ *TRPML1*$^{-/-}$ double knockout littermates. **(O)** Peaks per cell of the experiments in (M) and (N) *OCaR1*$^{-/-}$ *TRPML1*$^{+/+}$ n = 4 mice and *OCaR1*$^{-/-}$ *TRPML1*$^{-/-}$ n = 4 mice. Statistical analysis was done by two-tailed Student´s t-test (*$p \leq 0.05$).

Supplemental Figure 9

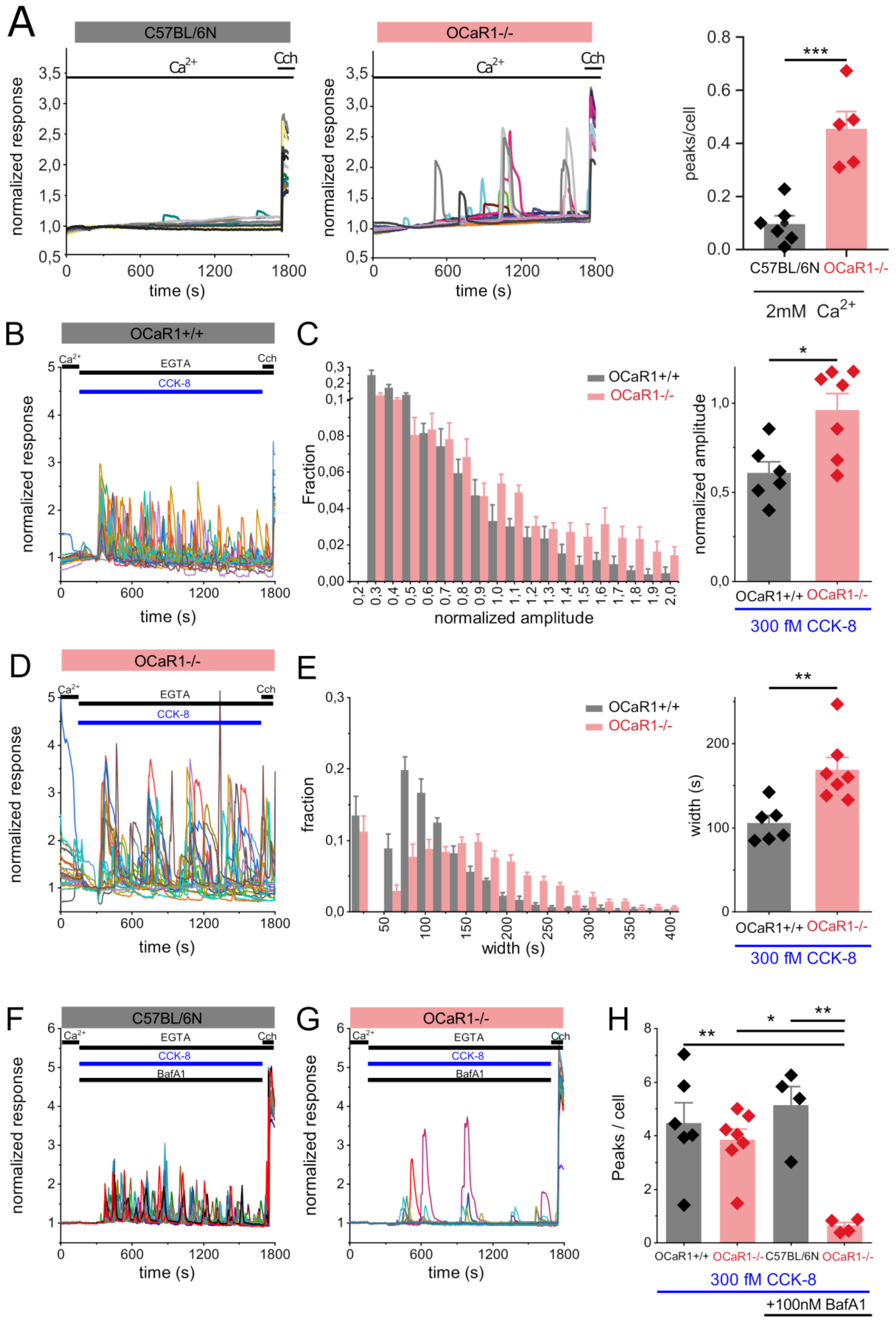

Figure S9 (related to Figures 3,4): Cholecystokinin evoked Ca^{2+} release in *OCaR1$^{-/-}$* and control cells

(A) Representative traces (25 cells per genotype) of Fura-2 fluorescence in pancreatic acinar cells of C57BL/6N and *OCaR1$^{-/-}$* mice under basal conditions with 2 mM extracellular Ca^{2+} and the peaks per cell, C57BL/6N n = 6 mice and *OCaR1$^{-/-}$* n = 5 mice. **(B)** Repesentative traces of Fura-2-fluorescence in cells from *OCaR1$^{+/+}$* mice in response to 300 fM CCK-8 in Ca^{2+}-free physiological solution. F340/380 values were normalized to the last time point prior to agonist application (time point 295 s). **(C)** Characterization of amplitude of the transients. Histogram indicating the fraction of occurrence of oscillations with defined amplitude and average amplitude of all transients are compared between *OCaR1$^{+/+}$* (n = 6 mice) and *OCaR1$^{-/-}$* (n = 7 mice). **(D)** *OCaR1$^{-/-}$* littermates from the experiment in (B). **(E)** Histogram characterizing the duration of the transients in (B) and (D). The fraction of occurrence of oscillations with defined duration are plotted and average width of all transients are compared between *OCaR1$^{+/+}$* (n = 6 mice) and *OCaR1$^{-/-}$* (n = 7 mice). **(F)** Representative traces of Fura-2-fluorescence in pancreatic islets in the presence of 300 fM CCK-8 and 100 nM Bafilomycin A1 (Baf A1) in Ca^{2+}-free physiological solution of C57BL/6N mice and **(G)** *OCaR1$^{-/-}$* mice. **(H)** Comparison of the peak frequency evoked by 300 fM CCK-8 in acini of *OCaR1$^{+/+}$* and *OCaR1$^{-/-}$* littermate mice without (*OCaR1$^{+/+}$* n = 6 mice, *OCaR1$^{-/-}$* n = 7 mice) or with (C57BL/6N n = 4 mice, *OCaR1$^{-/-}$* n = 4 mice) 90 min 100 nM Baf A1 pre-incubation. Statistical analysis was done by two-tailed Student´s t-test (A, C, E) or two-Way ANOVA with Bonferroni Post-Hoc test (H) (*$p \leq 0.05$; ** $p<0.01$, *** $p<0.001$).

Supplemental Figure 10

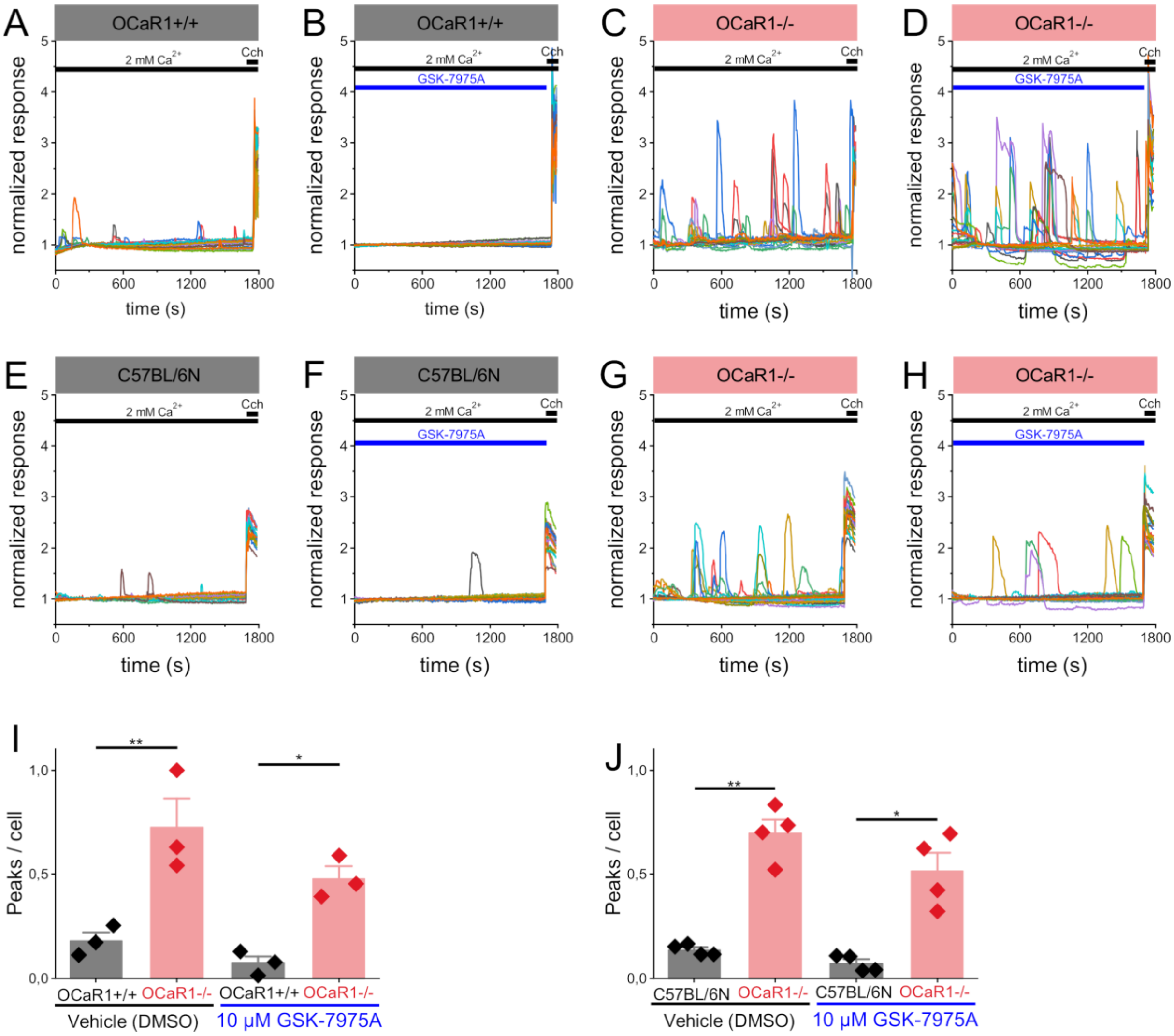

Figure S10: The effects of the Orai blocker GSK-7975A on intracellular calcium dynamics in the presence or absence of OCaR1. (A) Representative traces (25 cells per genotype) of Fura-2 fluorescence in pancreatic acinar cells of *OCaR1*$^{+/+}$ mice without and **(B)** with 10 min preincubation with 10 µM GSK-7975A under basal conditions with 2 mM extracellular Ca^{2+}. **(C)** *OCaR1*$^{-/-}$ littermates from the experiments in (A) and **(D)** with GSK-7975A preincubation. **(E)** and **(F)**, similar as (A) and (B) but with C57BL/6N animals and **(G, H)** *OCaR1*$^{-/-}$ animals that are not littermates. **(I)** Peaks per cell from the experiments in (A-D), n = 3 mice per condition. **(J)** Peaks per cell from the experiments in (E-H), n = 4 mice per condition. Statistical analysis was done by two-tailed Student´s t-test (*p≤0.05; ** p<0.01).

Supplemental Figure 11

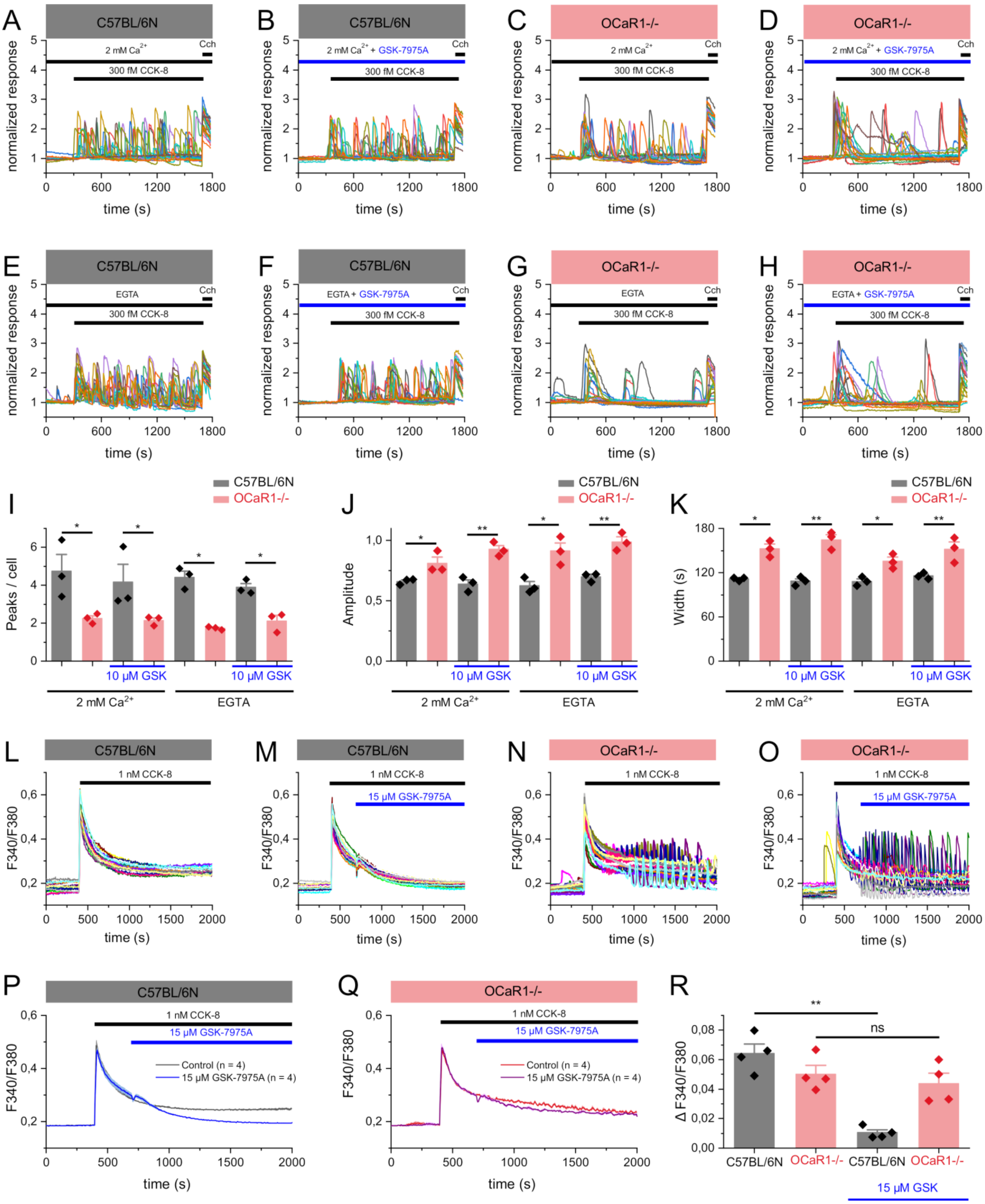

Figure S11: The effects of CRAC blocker GSK-7975A on intracellular calcium dynamics of C57BL/6N and *OCaR1$^{-/-}$* acinar cells.

Representative traces (25 cells per genotype) of Fura-2 fluorescence in pancreatic acinar cells stimulated with 300 fM CCK-8 in 2 mM extracellular calcium from **(A)** C57BL/6N mice in control conditions and **(B)** with 10 µM GSK-7975A. (**C**) and (**D**) as (A) and (B) but on cells isolated from *OCaR1$^{-/-}$* mice. **(E, F, G, H)** similar experiments as in (A-D) without extracellular calcium (0.25 mM EGTA) of C57BL/6N mice in (E) control conditions and (F) with 10 µM GSK-7975A. (G) Cells isolated from *OCaR1$^{-/-}$* mice in control conditions and (H) with 10 µM GSK-7975A. **(I)** The number of calcium peaks per cell **(J)** the amplitude and **(K)** the width of the peaks observed in (A-H), n = 3 mice per group. **(L)** Representative traces (n = 20) from Fura-2 responses on 1 nM CCK-8 in acinar cells from C57BL/6N mice without and **(M)** with application of 15 µM GSK-7975A during the plateau phase. **(N)** Representative traces (n = 20) from Fura-2 responses on 1 nM CCK-8 in acinar cells from *OCaR1$^{-/-}$* mice without and **(O)** with application of 15 µM GSK-7975A during the plateau phase. Note the oscillatory calcium pattern in these *OCaR1$^{-/-}$* cells. **(P)** Fura-2 fluorescence representing the average response of acinar cells isolated from C57BL/6N mice to 1 nM CCK-8 (in 5 mM extracellular calcium) with and without application of 15 µM GSK-7975A after the initial calcium peak, as in (L and M). **(Q)** Responses from acinar cells isolated from *OCaR1$^{-/-}$* mice stimulated 1 nM CCK-8 with and without the application of 15 µM GSK-7975A after the initial calcium peak as in (N and O). **(R)** The average response in the plateau phase at t = 2000 s of the experiments in (P) and (Q), n = 4 mice per group, two sample t-test, ns $p>0.05$, *$p<0.05$, **$p<0,01$.

Supplemental Figure 12

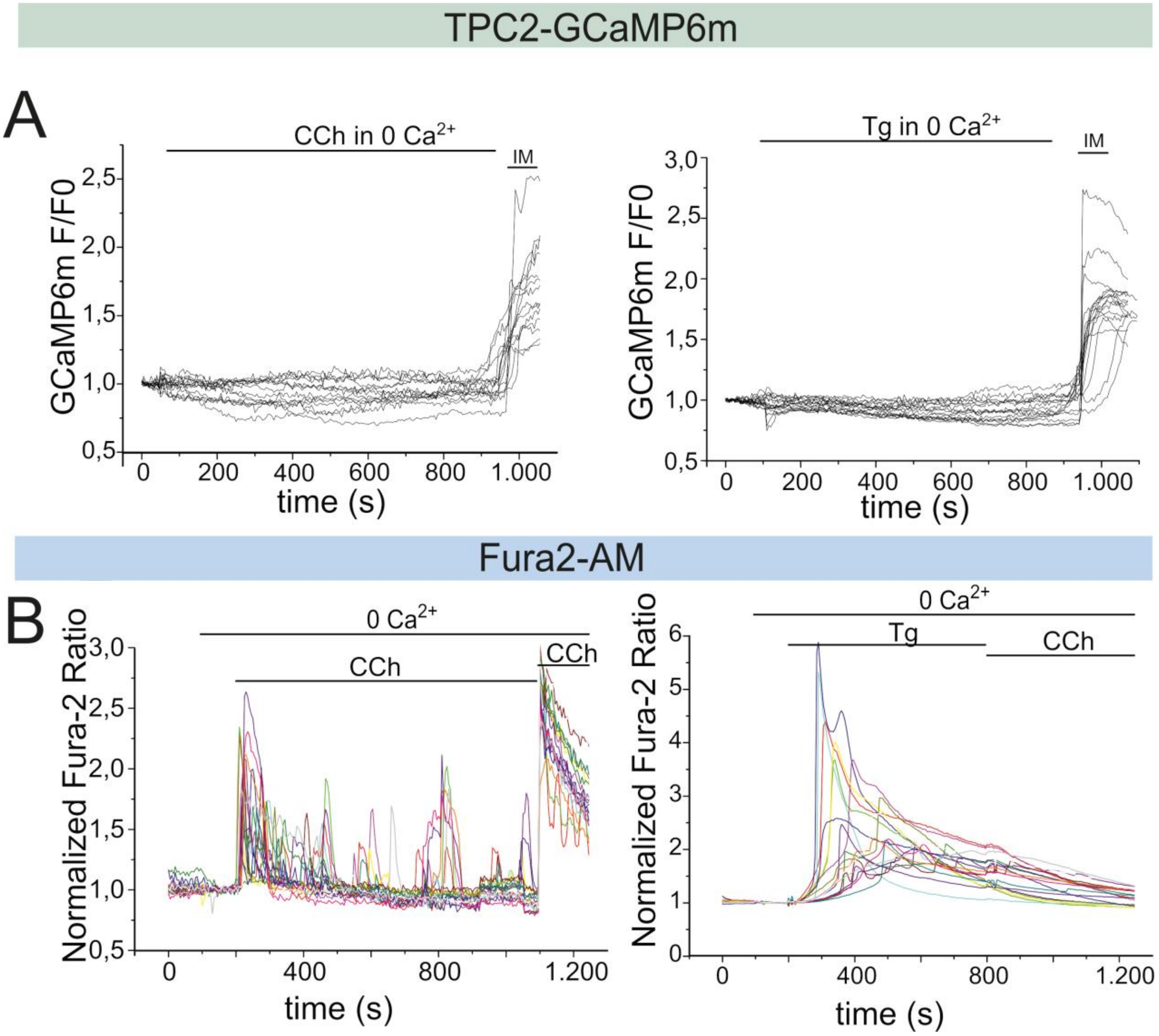

Figure S12 (Related to Figure 4): TPC2-GCaMP6m does not detect Ca^{2+} release from IP3-sensitive Ca^{2+} stores

(A) Local Ca^{2+} release from ER in acinar cells could not be observed by TPC2-GCaMP6m when stimulated with 100 nM Carbachol (n = 29) or 500 nM thapsigargin (n = 27) in Ca^{2+}-free solution. Ionomycin (IM, 10 µM) was applied as a positive control to evoke GCaMP6m fluorescence (measured as change of GCaMP6m fluorescence F over basal fluorescence F_0; F/F_0) under low external Ca^{2+} (nominally free Ca^{2+} + 0.25 mM EGTA) in freshly isolated pancreatic acinar cells from WT mice derived from zygotes transduced with a lentiviral vector expressing TPC2-GCaMP6m under control of PGK promotor (1.5-4 integrants per genome). **(B)** Measurements of global intracellular Ca^{2+} performed using Fura-2 demonstrate Ca^{2+} release evoked by 100 nM Carbachol (left panel) or 500 nM thapsigargin (right panel). 20 representative traces from 65 cells from 3 mice. CCh: Carbachol, Tg: Thapsigargin, IM: ionomycin.

Supplemental Figure 13

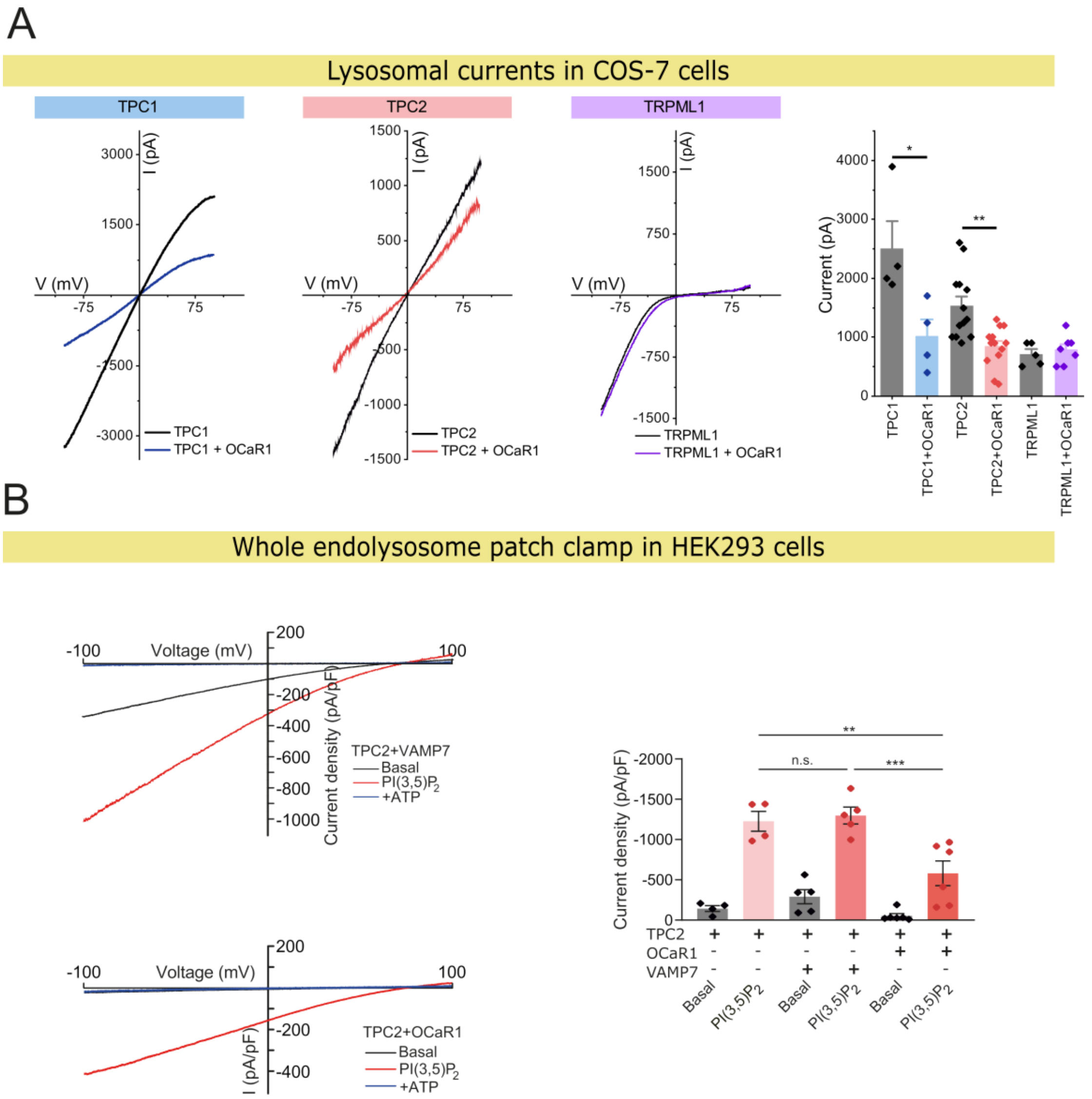

Figure S13 (related to Figure 4): OCaR1 inhibits TPC1 and TPC2-mediated currents, but not TRPML1-mediated currents.
Currents measured in enlarged endo-lysosomes from COS-7 cells overexpressing empty vector or OCaR1 with either TPC1, TPC2 or TRPML1. **(A)** Current-voltage relationship of TPC1, TPC2 or TRPML1 currents activated by PI(3,5)P_2 (1 µM) presented in COS-7 cells. (right) Mean current amplitudes of TPC1, TPC2 and TRPML1-currents measured at -60 mV (TPC1 n = 4, TPC1 + OCaR1 n = 4, TPC2 n = 13, TPC2 + OCaR1 n = 13, TRPML1 n = 5 and TRPML1 + OCaR1 n = 7). **(B)** Representative PI(3,5)P_2 (1 µM)-activated current densities in vacuolin-enlarged lysosomal vesicles isolated from HEK293 cells overexpressing mTPC2-RFP + VAMP7-YFP and mTPC2-RFP + OCaR1-YFP plasmids, respectively. Activated currents were blocked with ATP (1 mM) (n = 4-6). Statistical summary of PI(3,5)P_2 activated current densities. Average basal and PI(3,5)P_2-activated current densities (mean ± SEM) at -100 mV of endo-lysosomes expressing mTPC2 together with OCaR1, VAMP7 or empty vector control are shown. Statistical analysis was done by unpaired two-tailed Student´s t-test (* p≤0.05; ** p<0.01, *** p<0.001).

Supplemental Figure 14

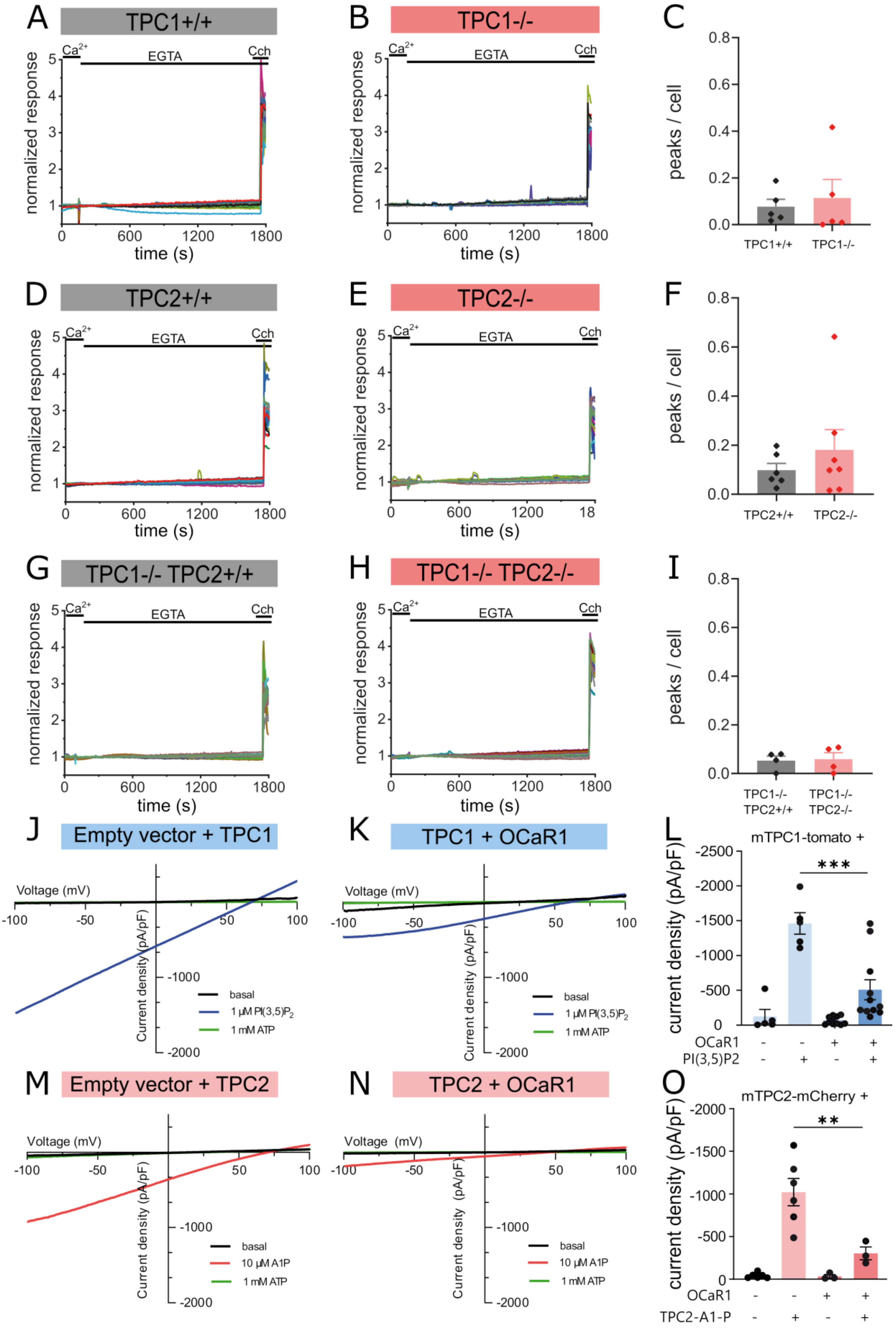

Figure S14 (related to Figure 4): TPC1 and TPC2 channels; their effect on intracellular calcium events and their inhibition with OCaR1

(A) Representative traces (25 cells per genotype) of Fura-2 fluorescence in pancreatic acinar cells of *TPC1*$^{+/+}$ and **(B)** littermate *TPC1*$^{-/-}$ mice in the absence of extracellular calcium. **(C)** Peaks per cell of the experiments in (A) and (B) *TPC1*$^{+/+}$ n = 5 mice and *TPC1*$^{-/-}$ n = 5 mice. **(D, E)** As (A, B) but with **(D)** *TPC2*$^{+/+}$ and **(E)** *TPC2*$^{-/-}$ littermates. **(F)** Peaks per cell of the experiments in (D) and (E) *TPC2*$^{+/+}$ n = 6 mice and *TPC2*$^{-/-}$ n = 7 mice. **(G, H)** As (A, B) but with **(G)** *TPC1*$^{-/-}$ *TPC2*$^{+/+}$ and **(H)** *TPC1*$^{-/-}$ *TPC2*$^{-/-}$ littermates. **(I)** Peaks per cell of the experiments in (G) and (H) *TPC1*$^{-/-}$ *TPC2*$^{+/+}$ n = 4 mice and *TPC1*$^{-/-}$ *TPC2*$^{-/-}$ n = 4 mice. **(J)** Representative current densities elicited by the application of PI(3,5)P_2 (1 µM) in vacuolin-enlarged endo-lysosomal vesicles isolated from HEK293 cells overexpressing *TPC1* and **(K)** *TPC1+OCaR1*. Activated currents were blocked with ATP (1 mM). **(L)** Statistical summary of PI(3,5)P_2 activated current densities. Average basal and activated current densities (mean ± SEM) at -100 mV of endo-lysosomes expressing *TPC1* together with *OCaR1* or empty vector control are shown. *TPC1* n = 5 cells, *TPC1+OCaR1* n = 11 cells. **(M)** Representative current densities elicited by the application of TPC2-A1-N (10 µM) in vacuolin-enlarged endo-lysosomal vesicles isolated from HEK293 cells overexpressing *TPC2* and **(N)** *TPC2+OCaR1*. Activated currents were blocked with ATP (1 mM). **(O)** Statistical summary of TPC2-A1-N activated current densities. Average basal and activated current densities (mean ± SEM) at -100 mV of endo-lysosomes expressing *TPC2* together with *OCaR1* or empty vector control are shown. *TPC2* n = 6 cells, *TPC2+OCaR1* n = 3 cells. Statistical analysis was done by two sample t-test (C, F, I) or one way ANOVA, followed by Tukey's post hoc test (L, O) (** p<0.01, *** p<0.001).

Supplemental Figure 15

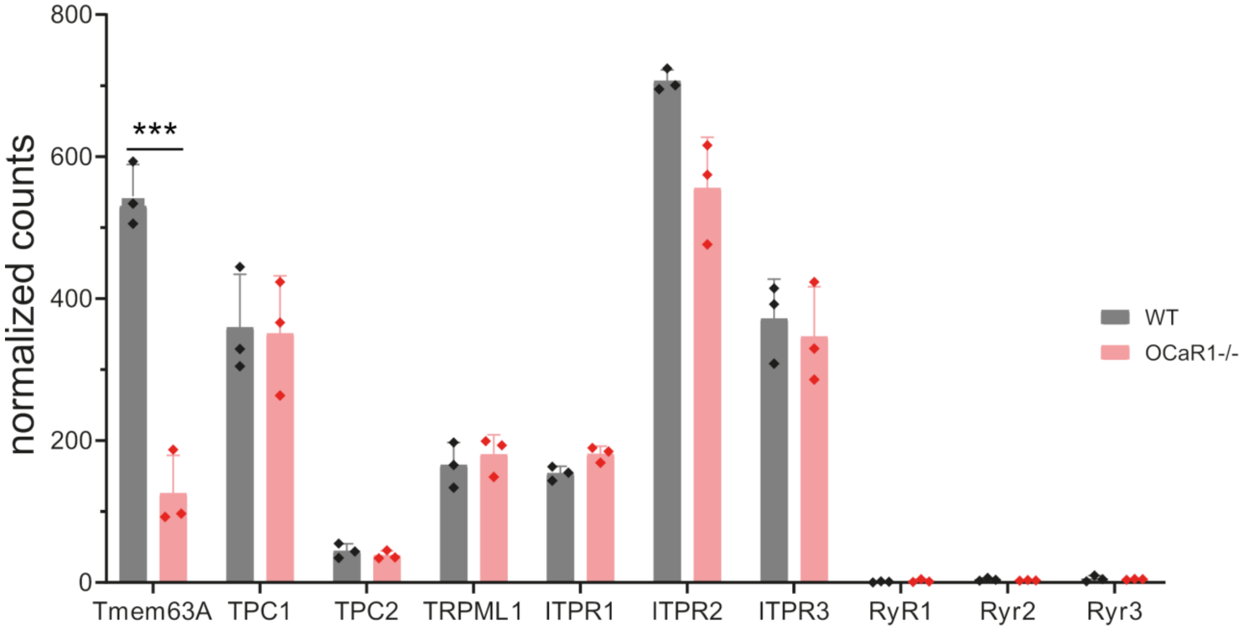

Figure S15 (Related to Figure 4): Unaltered expression of endo-lysosomal ion channels and Ca^{2+} release channels in the pancreas of *OCaR1$^{-/-}$* mice
Normalized gene transcript level abundance estimates in WT (black) and *OCaR1$^{-/-}$* (red) pancreatic tissue derived from transcriptome analysis of poly(A)+ RNA (n = 3 per genotype). Genes encoding endo-lysosomal cation channels and Ca^{2+} release channels are indicated.

Supplemental Figure 16

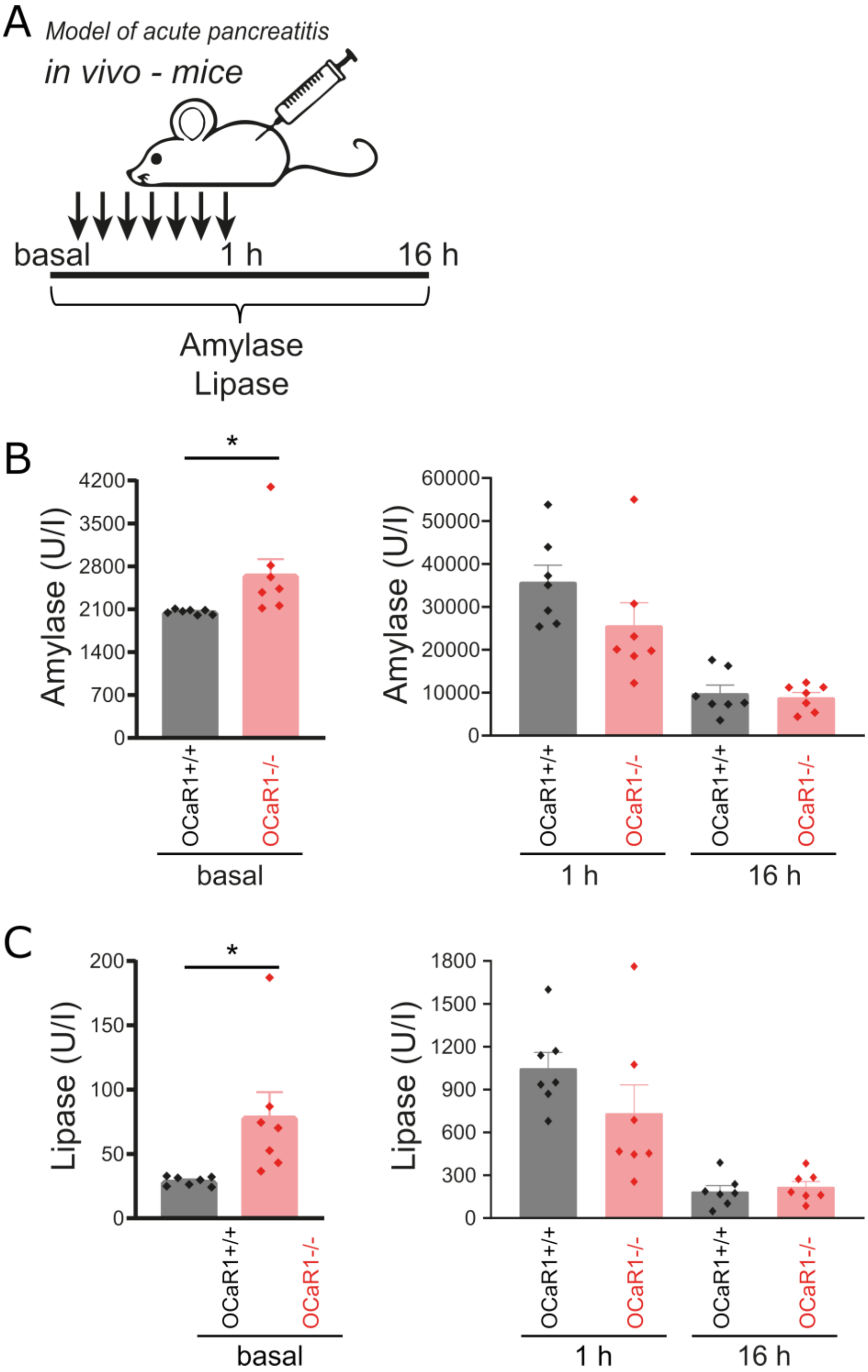

Figure S16 (Related to Figure 5): Induction of acute mild pancreatitis in *OCaR1$^{-/-}$* mice

(A) Schematic overview of mild acute pancreatitis induction by seven hourly injections (0.05 µg/g body weight) of cerulein in male mice. Plasma amylase **(B)** and lipase **(C)** levels under resting conditions (basal, 3 days before first cerulein injection) and 1 h or 16 h after the last of seven cerulein injections in *OCaR1$^{+/+}$* (n = 7) and *OCaR1$^{-/-}$* (n = 7) littermate mice. Statistical analysis was performed by unpaired two-tailed Student´s t-test (* p≤0.05).

Supplemental Figure 17

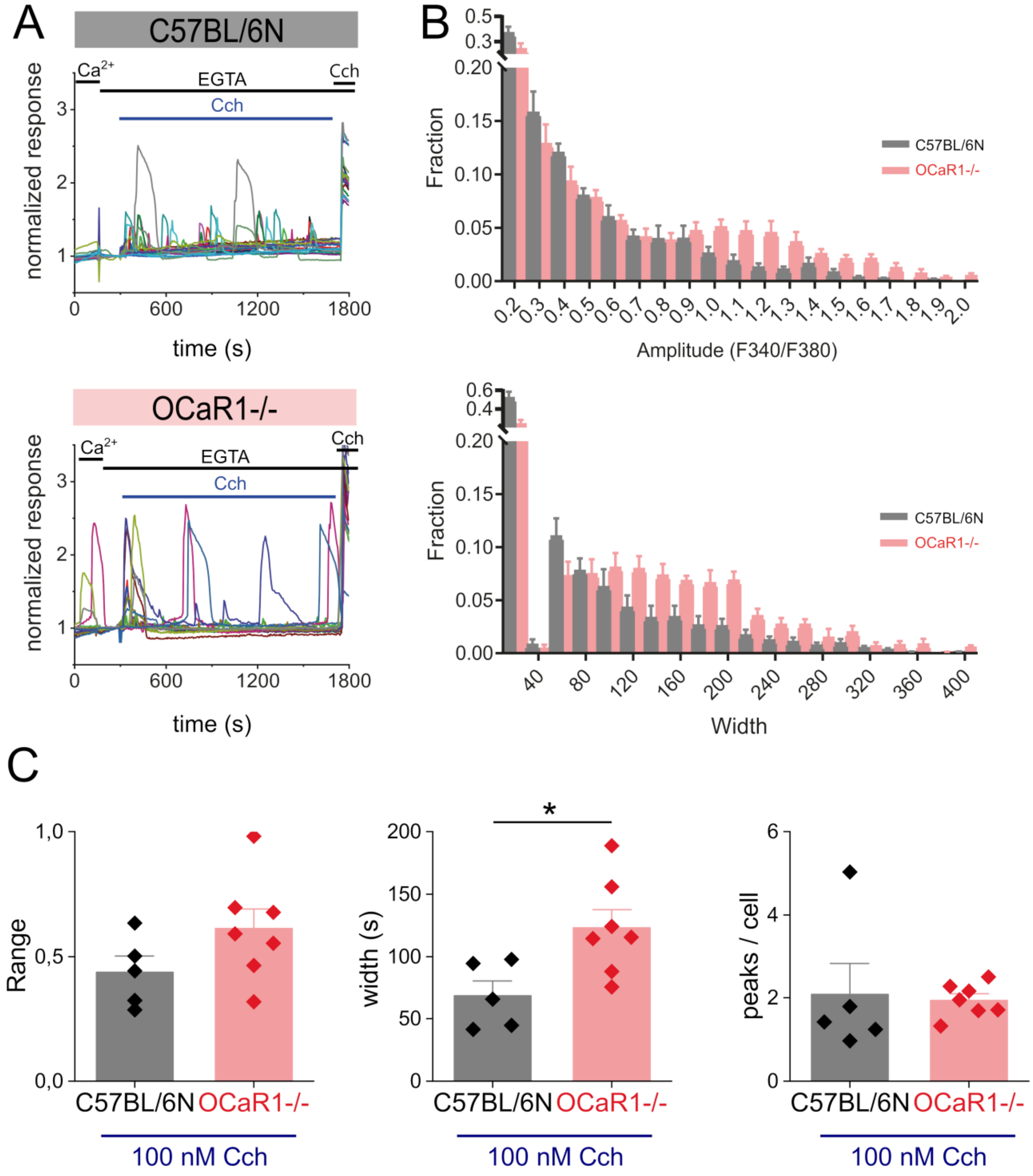

Figure S17 (Related to Figure 4): Carbachol evoked Ca^{2+} release in *OCaR1*$^{-/-}$ and control cells

(A) Representative traces (25 cells per genotype) of Fura2-AM loaded pancreatic acinus cells from C57BL/6N (upper panel) and *OCaR1*$^{-/-}$ (bottom panel) mice in response to 100 nM Cch in a Ca^{2+}-free physiological solution. F340/380 values were normalized to the last time point prior to agonist application (time point 295 s). **(B, C)** Analysis of the amplitude and width of Ca^{2+} transients as well as frequency of oscillations in C67BL/6N (n = 5) and *OCaR1*$^{-/-}$ (n = 7) mice (as in Figure S9). The unpaired two-tailed Student´s t-test was used for statistical analysis (* $p \leq 0.05$).

Supplemental Figure 18

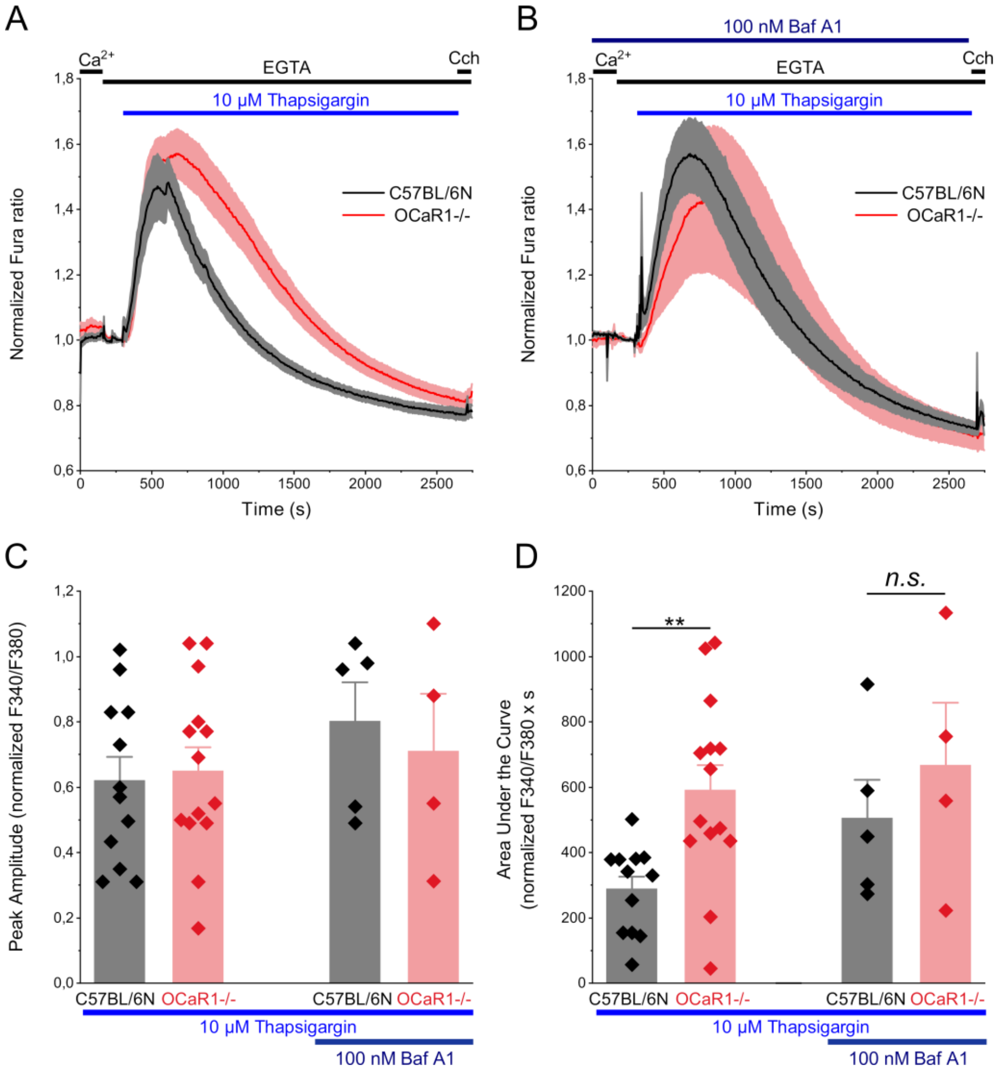

Figure S18: Intracellular calcium dynamics during thapsigargin-induced passive depletion of ER in C57BL/6N and *OCaR1*$^{-/-}$ acinar cells. (A) The average effect of 10 µM thapsigargin (Tg) on pancreatic acinar cells in calcium-free extracellular conditions. The observed slow calcium transient due to ER depletion seems larger in *OCaR1*$^{-/-}$ acinar cells. **(B)** After 90 min preincubation of the cells with 100 nM Baf A1 to disrupt calcium storage in acidic organelles, the acinar cells of C57BL/6N or *OCaR1*$^{-/-}$ mice were exposed to 10 µM thapsigargin to induce passive ER calcium depletion. *OCaR1*$^{-/-}$ acinar cells treated with Baf A1 seem to have similar calcium capacity in their ER compared to C57BL/6N cells. **(C)** The maximum amplitude reached of the experiments in (A) and (B). The traces were baseline corrected by subtracting a linear baseline from t = 295 s (immediately before Tg application) and the lowest point at t = 2700 s immediately before the application of Cch. **(D)** The area under the curve from the traces in (A) and (B). The total calcium released from the ER of *OCaR1*$^{-/-}$ cells is higher than in the C57BL/6N cells. This effect is not present when the cells are treated with Baf A1. (C57BL/6N; Tg: n = 12, Tg + Baf A1: n = 5. *OCaR1*$^{-/-}$; Tg: n = 14, Tg + Baf A1: n = 4). Two-way Anova with Bonferroni corrected pairwise post-hoc comparison, (*n.s.* $p>0.05$, **$p<0.01$).

Supplemental Figure 19

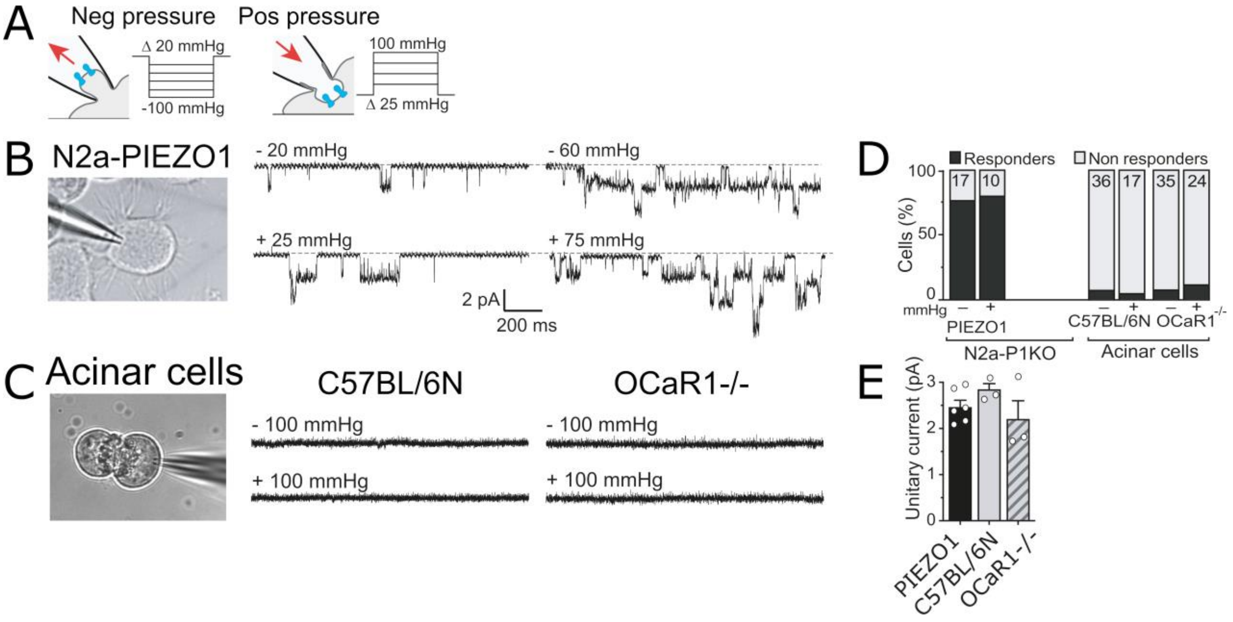

Figure S19 (Related to Figure 4): OCaR1 deletion does not affect mechano-sensitive currents in the plasma membrane of primary acinar cells.
(A) Mechanically-activated currents were evoked by applying a series of brief pulses (1 s duration) of negative (increment 20 mmHg) and positive (increment 25 mmHg) pressure to the cell membrane via the patch pipette in cell-attached patch-clamp recordings. **(B)** Representative example traces of single channel openings of PIEZO1 expressed in N2a-PIEZO1-KO cells evoked by negative (top traces) and positive (bottom traces) pressure. **(C)** Neither negative nor positive pressure elicits single channel openings in pancreatic acinar cells. **(D)** Comparison of the proportions of N2a-PIEZO1-KO cells transfected with PIEZO1, acinar cells from C57BL/6N mice, and acinar cells from *OCaR1*$^{-/-}$ mice that exhibited inward currents in response to stimulation with negative (-) and positive (+) pressure. The number of cells is indicated in the figure. **(E)** Comparison of the unitary current amplitudes of PIEZO1 (black bar) and the endogenous pressure-activated current in acinar cells that was observed in 3/36 tested acinar cells from C57BL/6N and in 3/35 tested acinar cells from *OCaR1*$^{-/-}$ mice.

Supplemental Figure 20

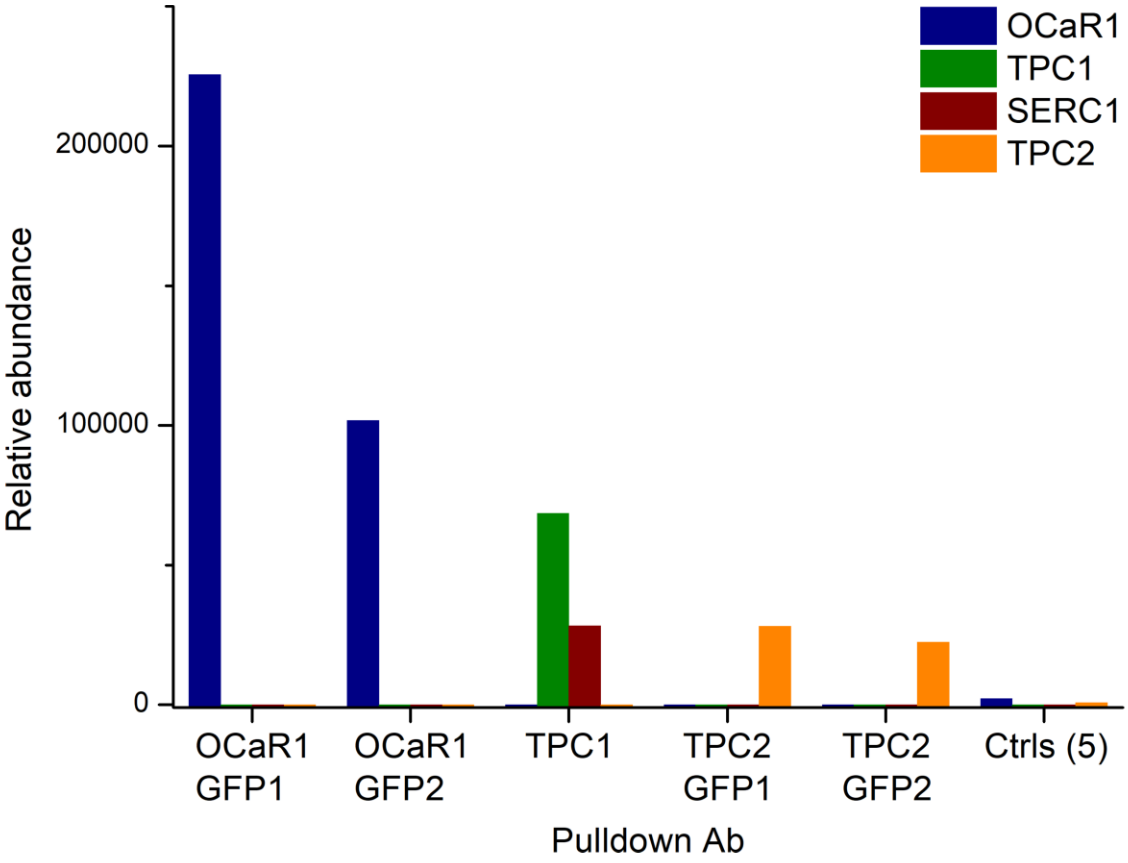

Figure S20: Mass spectrometry analysis of affinity purification of OCaR1, TPC1 and TPC2. AP-MS analysis of mouse pancreatic acinar cells. *OCaR1*$^{eYFP/eYFP}$ mice and *TPC2-GCaMP* and C57BL/6N mice were used. Two different GFP antibodies were used to pull down OCaR1-eYFP and associated proteins, similarly anti-GFP antibodies were used in *TPC2-GCaMP* samples. For the pull down of TPC1, a combination of two TPC1 antibodies were used in samples of C57BL/6N mice. SERC1 was previously identified as an interaction partner of TPC1, and is included as a control. The relative presence of the different proteins detected in the pulled down fractions with the used antibody is indicated.

Table S1 (related to Figures 2-5): Segregation analysis of the OCaR1-null allele from intercrosses of *OCaR1*$^{+/-}$ mice

Analysis of the inheritance of the *OCaR1*$^{-}$ (null) allele

offspring	Σ +/+	Σ +/-	Σ -/-
**982**	**242**	**501**	**239**
**expected**	**25%**	**50%**	**25%**
**obtained**	**24.64%**	**51.02%**	**24.34%**
**Chi-square test**		**0.426**	
**p-value**		**0.8083**	

Table S2 (related to Figure S5): Sequence information of primers for cDNA amplification

List of oligonucleotides used for cDNA amplification

name	sequence (5´→3´)
AB_79	CGTCCAGCTCGACCAGGATGG
AB_80	GACCAGGATGTGAGATGTCAGG
AB_81	GCTGCCCGACAACCACTACC
AB_82	GTTGGCTACCCGTGATATTGC
AB_83	GGGTCCATGGTGATACAAGGG
AB_84	CTGCCAGACCAGCTTGTCC
AB_87	GGT TGT TCT GCT CAC CAT CC
AB_90	GTC CTC CTT GAA GTC GAT GC
UK79	CAG CTT TGG GAG GAC AAC G
UK81	GCA GAA CTG GCC ACA AGG

Table S3: Details of the used antibodies, cell lines and mouse lines.

Antibodies					
Antibody	Source	Catalog #	Clone #	Description	Figure
Rab27B	Synaptic Systems	168 103	Polyclonal		2, 4
anti-PMP70	abcam	ab3421	Polyclonal		S3
HRP secondary	Sigma	A5278-1ML	Polyclonal		S3
Anti-GFP	Roche	11814460001	7.1/13.1		S3
Anti-GFP	Invitrogen	A11122	Polyclonal		S20
Anti-GFP	R&D Systems	AF4240	Polyclonal		S20
Anti-TPC1	Custom		836	Castonguay et al. *Sci Rep.* 2017 (23)	S20
Anti-TPC1	Custom		838	Castonguay et al. *Sci Rep.* 2017 (23)	S20
Control IgG	Upstate Biotechnology - Millipore	12-370	Polyclonal		S20
**Cell lines**					
Cell line	Source				
HEK293	ATCC				
COS-7	ATCC				
**Used Animals**					
Transgenic mice		Source	Custom Description		
OCaR1 knock out		Custom	Methods section		
OCaR1-IRES GFP reporter		Custom	Methods section		
OCaR1$^{eYFP/eYFP}$ Knock-Add-On		Custom	Methods section		
Lamp1-RFP		Custom	Methods section		
TPC2-GCaMP6m		Custom	Methods/ Suppl. Methods		
TRPML1 Knock-Out		Custom	Suppl. Methods		
TPC1 Knock-Out		Custom	Arndt et al. *Mol Biol Cell.* 2014 (5)		
TPC2 Knock-Out		Custom	Grimm et al. *Nat Commun.* 2014 (7)		
C57BL/6N		Charles River			
