# Supplementary material for: OCaR1 endows exocytic vesicles with autoregulatory competence by preventing uncontrolled Ca2+ release, exocytosis, and pancreatic tissue damage
Source: J Clin Invest. 2024 Apr 1;134(7):e169428. doi: 10.1172/JCI169428 (PMC10977991; doi:10.1172/JCI169428)
Supplement: Unedited blot and gel images [file jci-134-169428-s007.pdf]

Original gels and blots for:

OCaR1 endows exocytic vesicles autoregulatory competence preventing uncontrolled Ca^{2+} release, exocytosis, and pancreatic tissue damage

Volodymyr Tsvilovskyy*, Roger Ottenheijm*, Ulrich Kriebs*, Aline Schütz*, Kalliope Nina Diakopoulos*, Archana Jha, Wolfgang Bildl, Angela Wirth, Julia Böck, Dawid Jaślan, Irene Ferro, Francisco J. Taberner, Olga Kalinina, Staffan Hildebrand, Ulrich Wissenbach, Petra Weissgerber, Dominik Vogt, Carola Eberhage, Stefanie Mannebach, Michael Berlin, Vladimir Kuryshev, Dagmar Schumacher, Koenraad Philippaert, Juan E. Camacho-Londoño, Ilka Mathar, Christoph Dieterich, Norbert Klugbauer, Martin Biel, Christian Wahl-Schott, Peter Lipp, Veit Flockerzi, Hans Zischka, Hana Algül, Stefan G. Lechner, Marina Lesina, Christian Grimm, Bernd Fakler, Uwe Schulte, Shmuel Muallem, Marc Freichel

This document contains the original uncropped and unedited versions of all gel and blot images that appear in the manuscript and its supplemental data. This data was collected by different scientist at different universities with a range of different tools to their disposal. The earliest experiments in this projects are many years older than the most recent experiments. All images were recorded and stored due to the standards that were applicable at the time and the place that the experiments happened. Inconsistencies in image quality can easily be explained by the vast improvement that has been made in imaging equipment in the time span that experimental data for this manuscript was gathered.
These gels and blots contain additional experimental data that is not described in the manuscript, including, but not limited to, bands that appear in other lanes from the same gel and hand written notes and experimental details written on the blot images. This additional experimental data is only present to illustrate to the reviewing team that no purposeful misleading image manipulation occurred in the images represented in the manuscript and the supplemental figures. Omitting this data would render such evaluation problematic. The information not contained in the figures or the supplemental figures of the manuscript cannot be interpreted in the context of the current manuscript. Neither should the dissemination of the full gel images be considered publication of these results. The right to use fractions of the images below that are not included in the main manuscript or the supplemental information remains maintained at the sole discretion of the responsible authors.

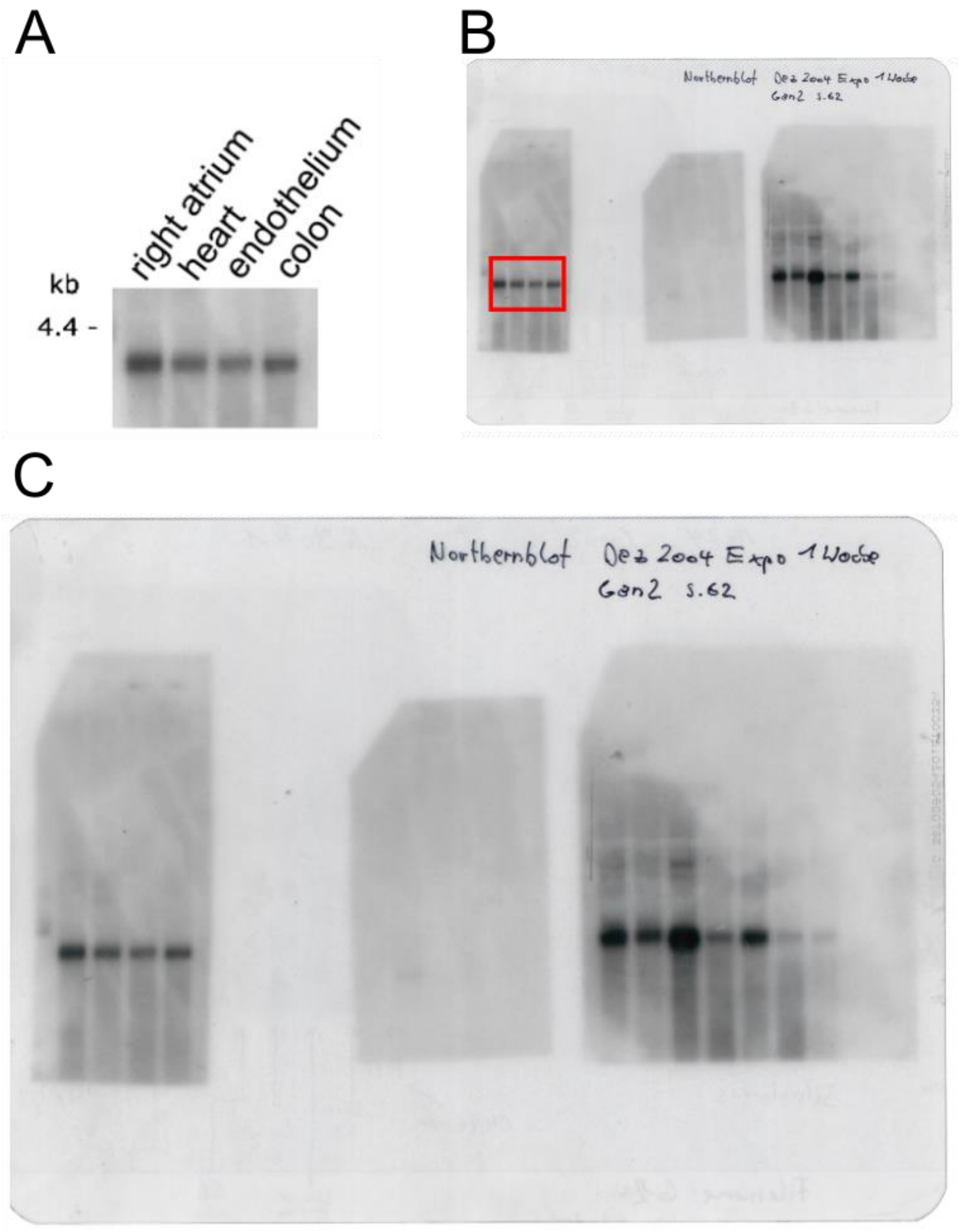

Figure G1: Full unedited gel for Supplemental figure S2A. (**A**) Copy of supplemental figure S2A. (**B**) Indication where the original gel was cropped. (**C**) The original gel. The writing on the gel “Northernblot Dez 2004 Expo 1 Woche Gen2 S.62” indicates that this was a northern blot ran in December 2004 with an exposure time of 1 week, probing for Gen2. S.62 is an experiment identifier. Gen2 was the name used at that time for what is now known as OCaR1.

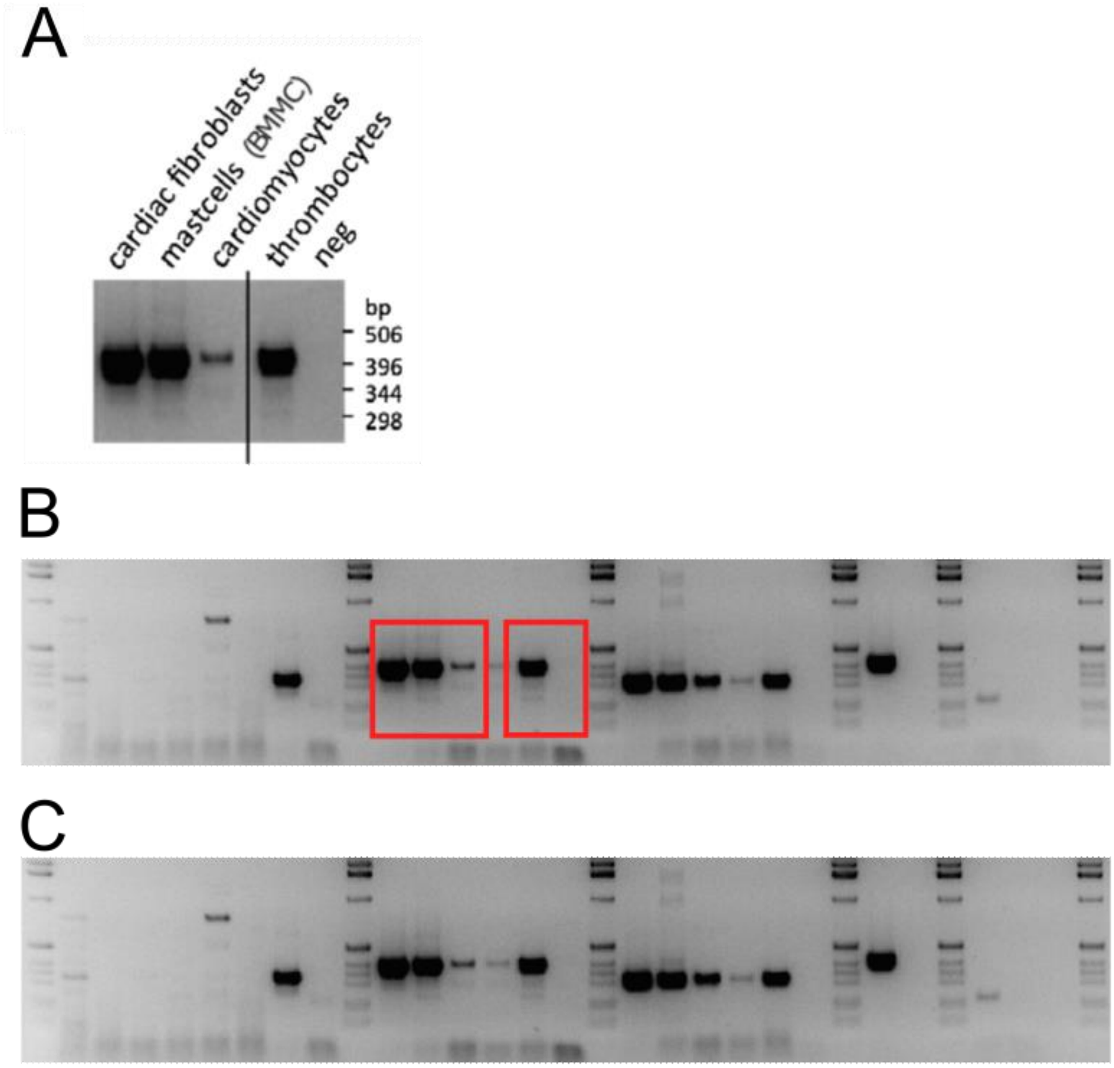

Figure G2: Full unedited gel for Supplemental figure S2B, left. (**A**) Copy of supplemental figure S2B, left. (**B**) Indication where the original gel was cropped. (**C**) The original gel. Note that the left part of figure S2B is a composite image of different lanes on the same gel. Both parts are displayed next to each other with a solid black line in between.

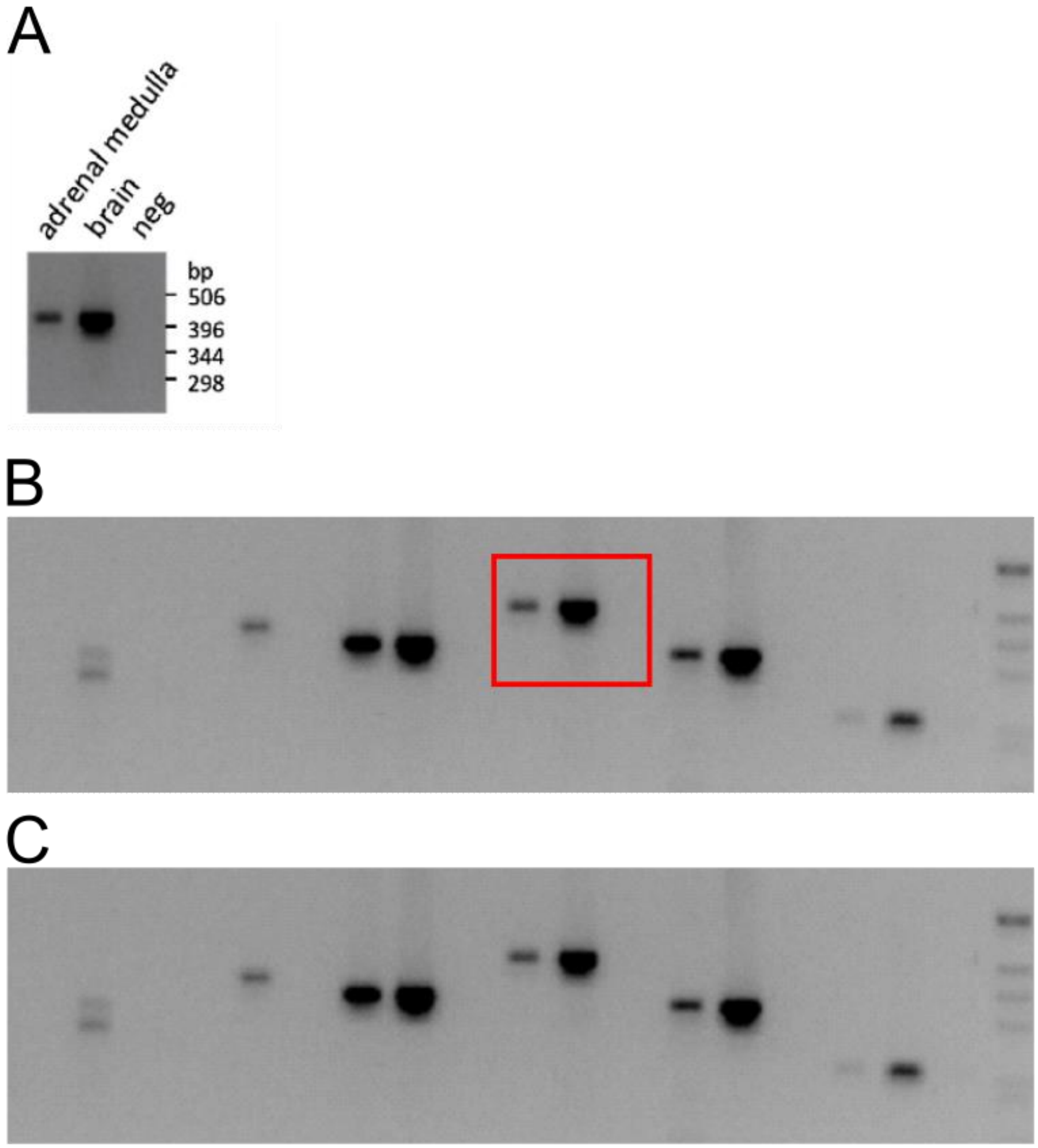

Figure G3: Full unedited gel for Supplemental figure S2B, right. (**A**) Copy of the right part of supplemental figure S2B. (**B**) Indication where the original gel was cropped. (**C**) The original gel.

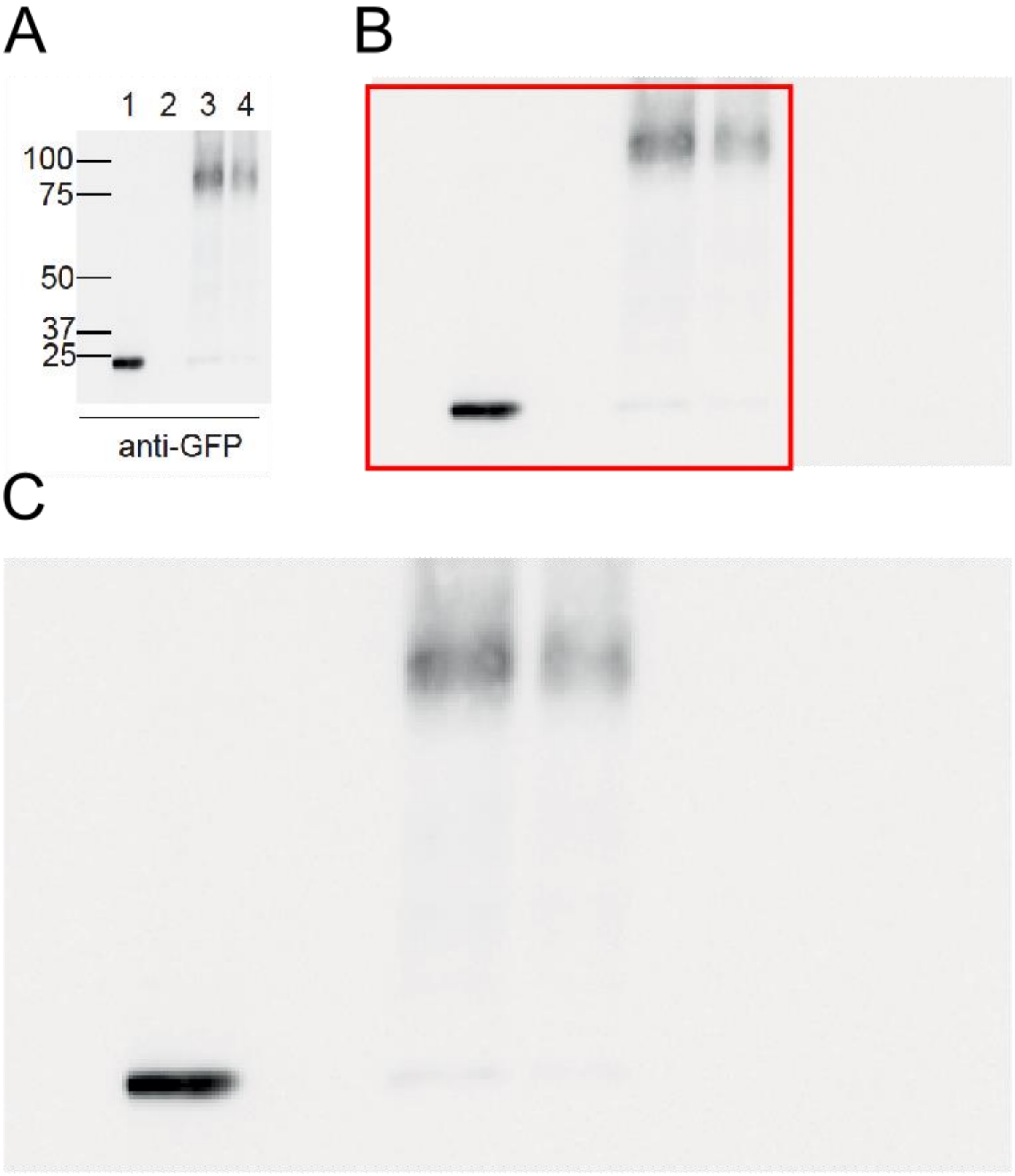

Figure G4: Full unedited gel for Supplemental figure S3A. (**A**) Copy of supplemental figure S3A. (**B**) Indication where the original gel was cropped. (**C**) The original gel.

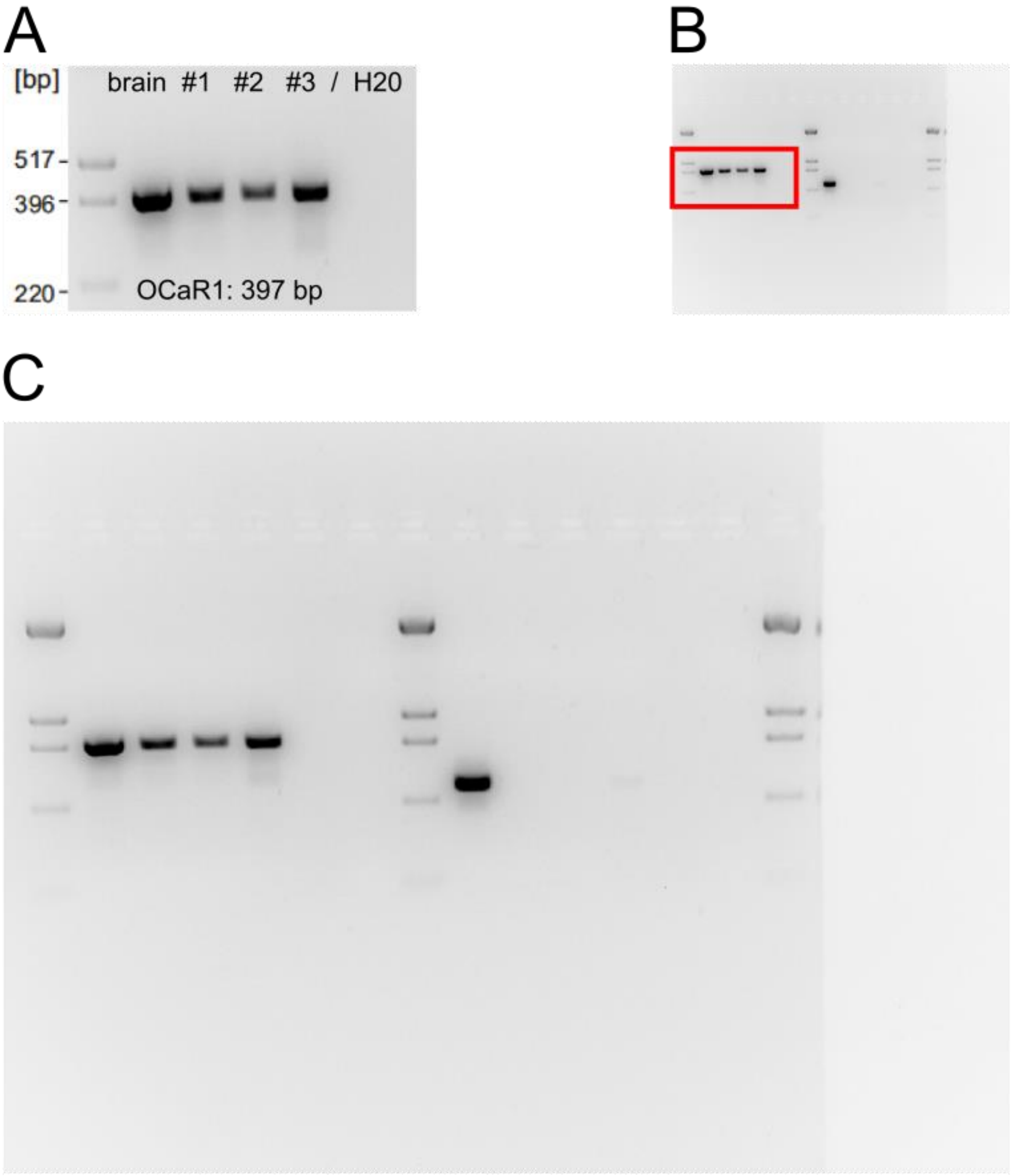

Figure G5: Full unedited gel for Supplemental figure S4F. (**A**) Copy of supplemental figure S4F. (**B**) Indication where the original gel was cropped. (**C**) The original gel.

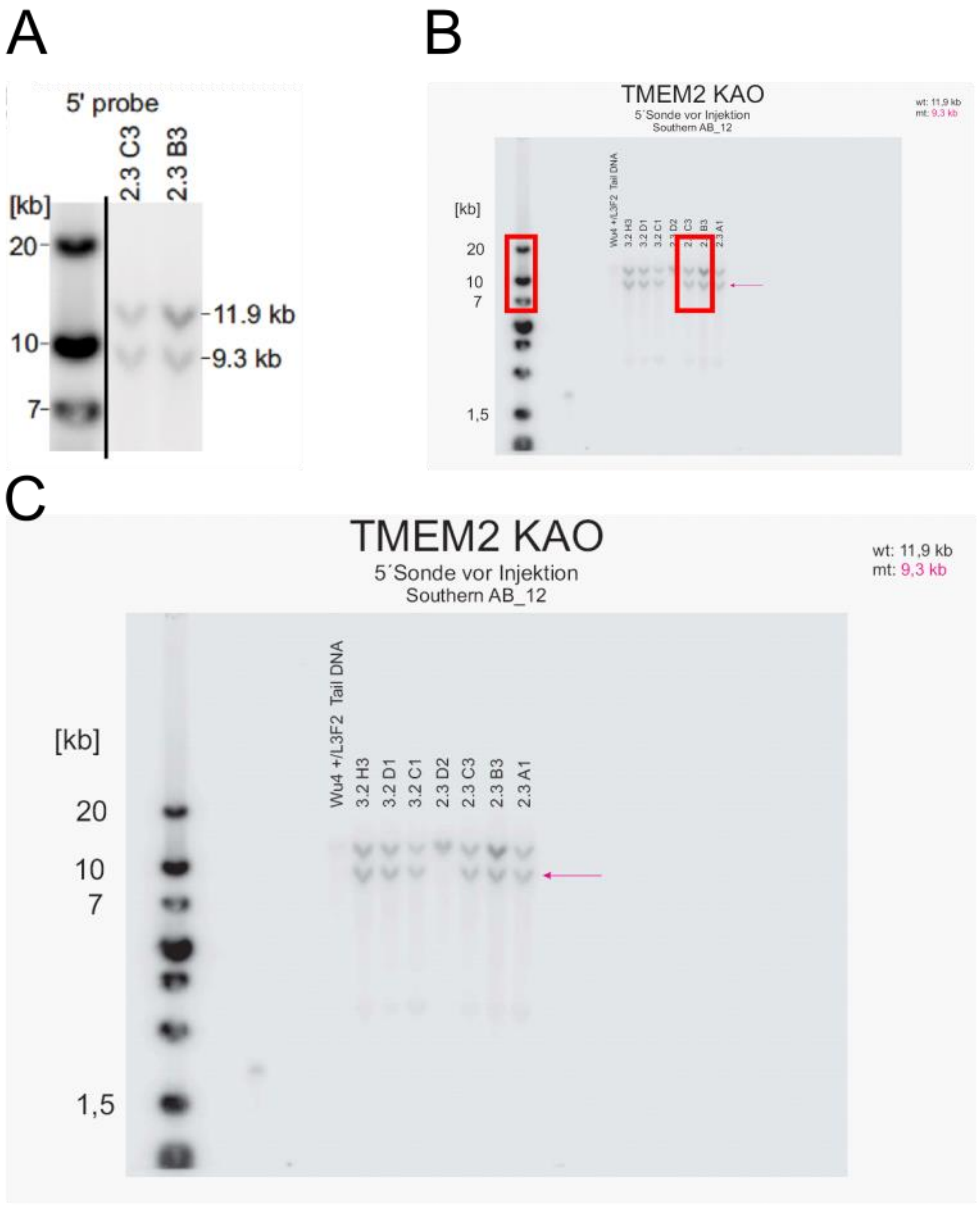

Figure G6: Full unedited gel for Supplemental figure S5B left. (**A**) Copy of supplemental figure S5B left. (**B**) Indication where the original gel was cropped. (**C**) The original gel. Note that the left part of figure S5B is a composite image of different lanes on the same gel. Both parts are displayed next to each other with a solid black line in between. The digital writing on the original gel indicates TMEM2 KAO (working name for OCaR1-eYFP), the identification of the southern probe: 5' Sonde vor Injektion and the identification of the Southern blot: Southern AB_12 in which AB identifies the experimental scientist, who is shared first author in the manuscript (Aline Schütz) and 12 is an experimental identifier. Wild-type (wt) and Mutant (mt) fragment lengths are indicated in the upper right, and indicated with an arrow on the gel. The writing on the individual lanes identifies the sample that was loaded there.

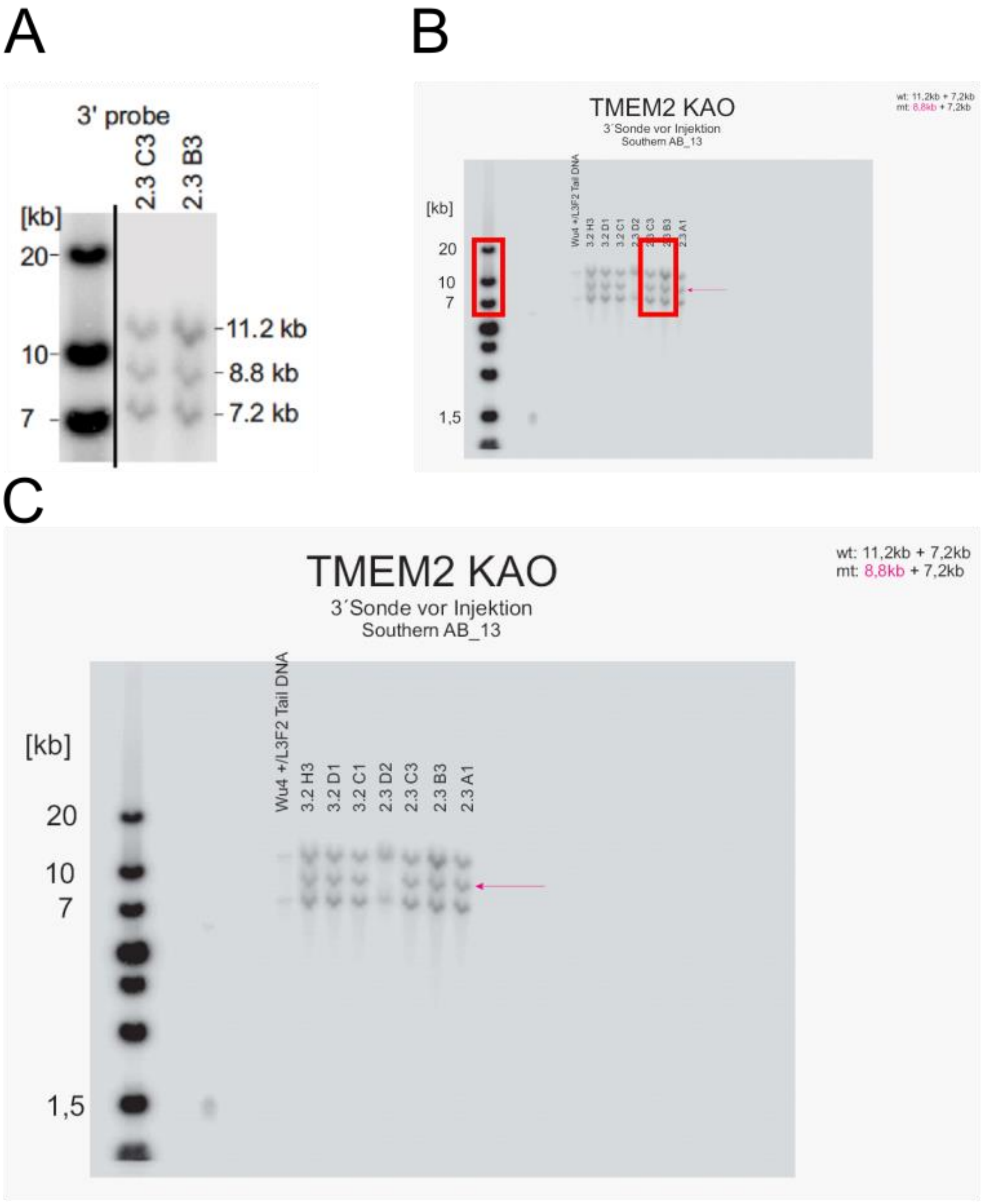

Figure G7: Full unedited gel for Supplemental figure S5B middle. (**A**) Copy of supplemental figure S5B middle. (**B**) Indication where the original gel was cropped. (**C**) The original gel. Note that the middle part of figure S5B is a composite image of different lanes on the same gel. Both parts are displayed next to each other with a solid black line in between. The digital writing on the original gel indicates TMEM2 KAO (working name for OCaR1-eYFP), the identification of the southern probe: 3' Sonde vor Injektion and the identification of the Southern blot: Southern AB_13 in which AB identifies the experimental scientist, who is shared first author in the manuscript (Aline Schütz) and 13 is an experimental identifier. Wild-type (wt) and Mutant (mt) fragment lengths are indicated in the upper right, and indicated with an arrow on the gel. The writing on the individual lanes identifies the sample that was loaded there.

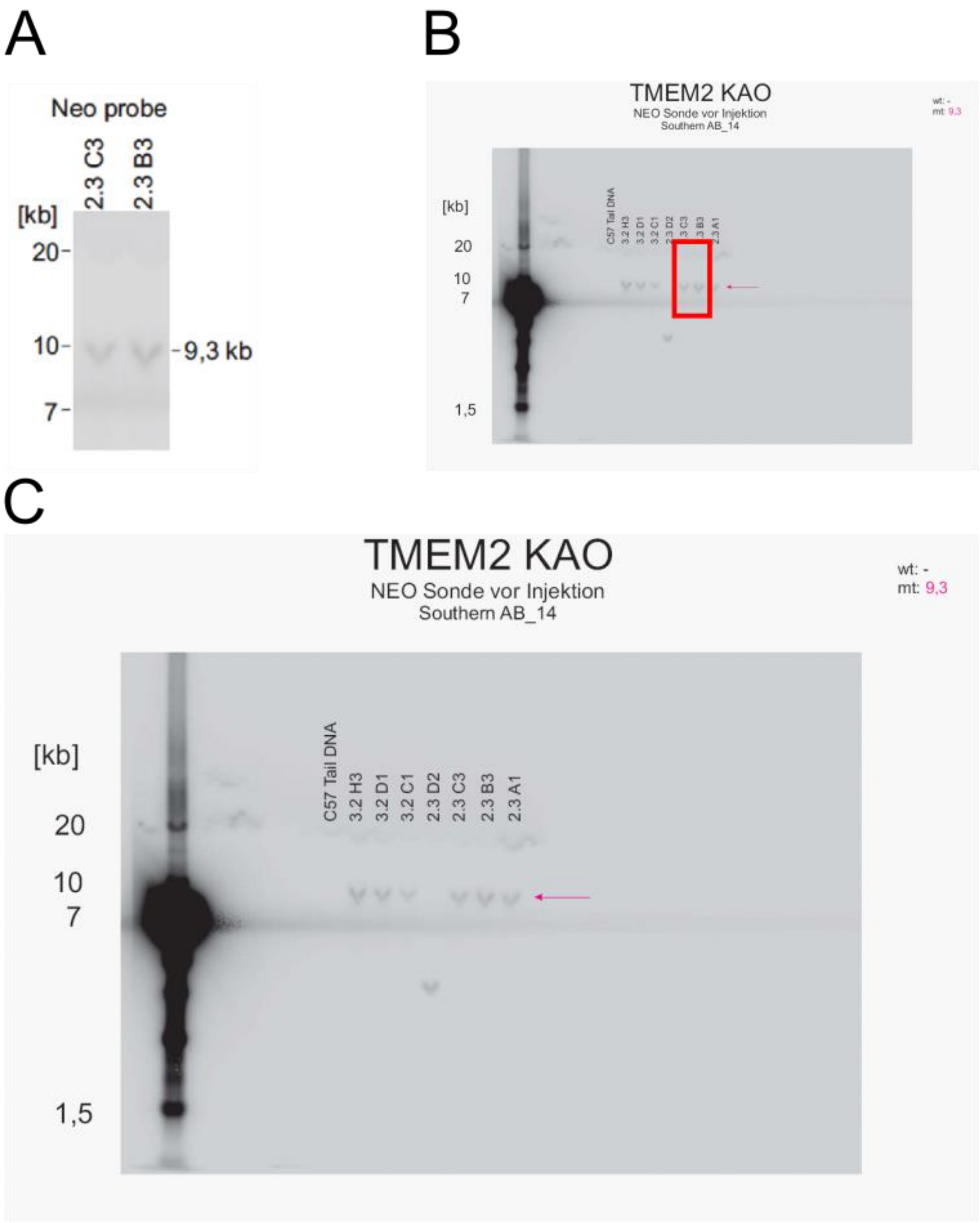

Figure G8: Full unedited gel for Supplemental figure S5B right. (**A**) Copy of supplemental figure S5B right. (**B**) Indication where the original gel was cropped. (**C**) The original gel. The digital writing on the original gel indicates TMEM2 KAO (working name for OCaR1-eYFP), the identification of the southern probe: NEO Sonde vor Injektion and the identification of the Southern blot: Southern AB_14 in which AB identifies the experimental scientist, who is shared first author in the manuscript (Aline Schütz) and 14 is an experimental identifier. Wild-type (wt) and Mutant (mt) fragment lengths are indicated in the upper right, and indicated with an arrow on the gel. The writing on the individual lanes identifies the sample that was loaded there.

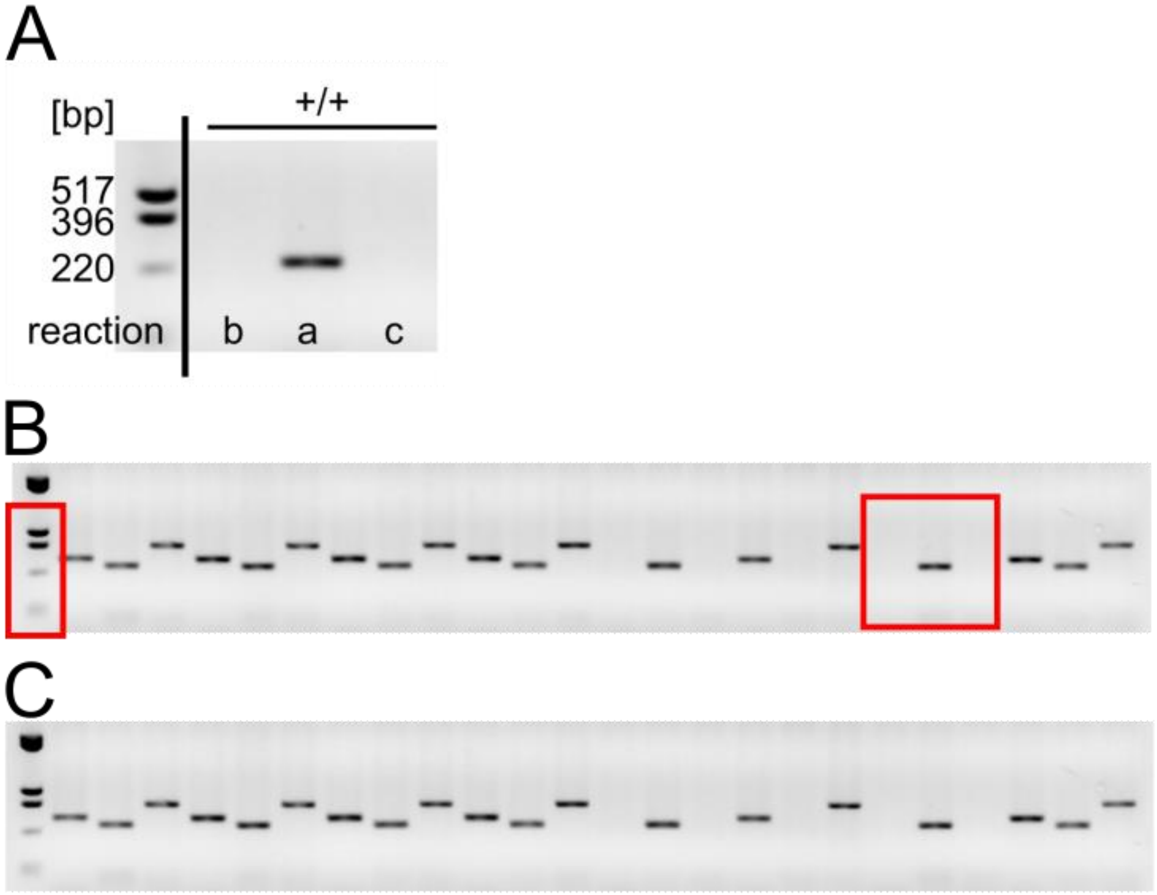

Figure G9: Full unedited gel for Supplemental figure S5D, left. (**A**) Copy of the left side of Supplemental figure S5D. (**B**) Indication where the original gel was cropped. (**C**) The original gel. Note that the left side of Supplemental figure S5D is a composite figure, where the size marker and the 3 experimental conditions from the same gel were displayed next to each other with a solid black line in between.

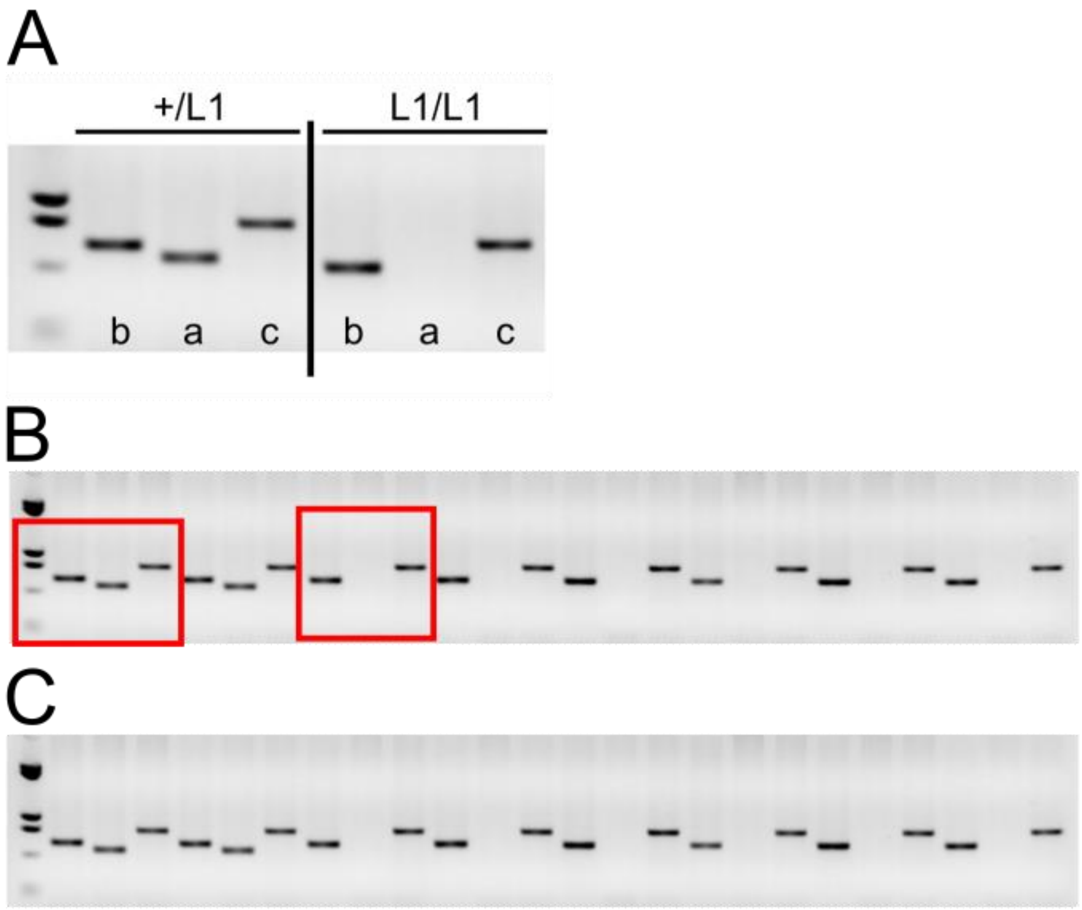

Figure G10: Full unedited gel for Supplemental figure S5D, middle. (**A**) Copy of the middle part of Supplemental figure S5D. (**B**) Indication of the two locations where the original gel was cropped. (**C**) The original gel. Note that the middle part of Supplemental figure S5D is a composite figure with noncontiguous lanes of the same gel, where the size marker and the first 3 experimental lanes are one crop and the right 3 lanes are a second crop. Both parts are displayed next to each other with a solid black line in between.

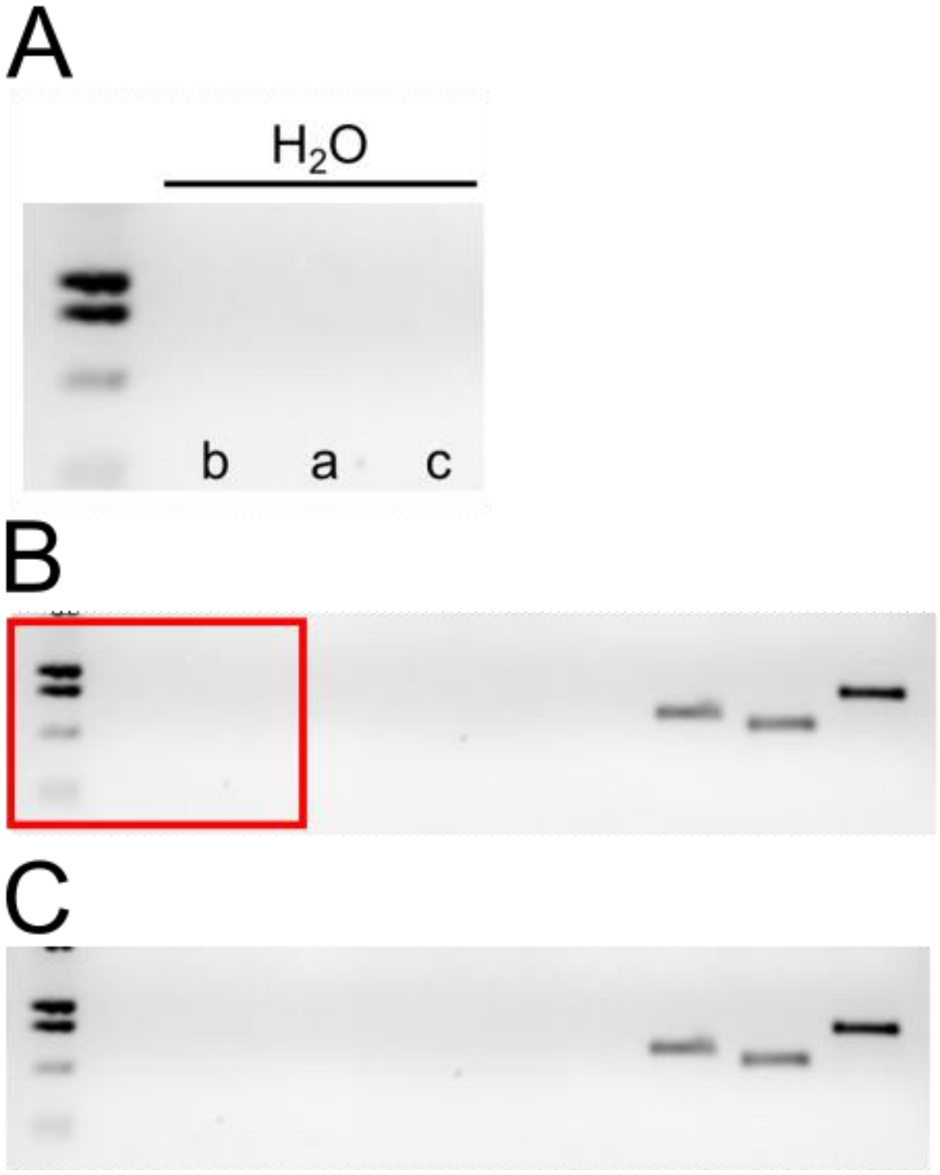

Figure G11: Full unedited gel for Supplemental figure S5D, right. (**A**) Copy of the right part of Supplemental figure S5D. (**B**) Indication where the original gel was cropped. (**C**) The original gel.

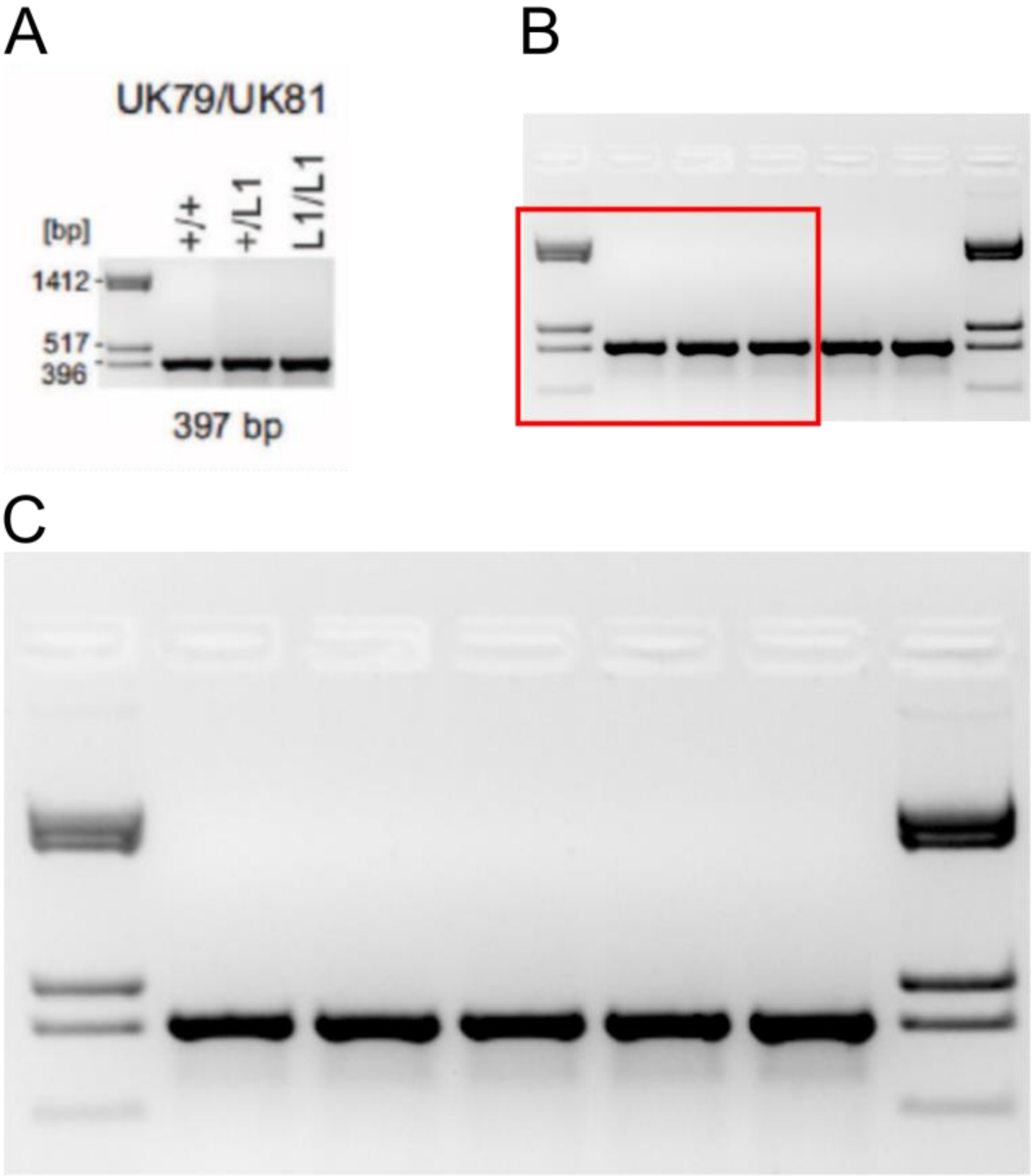

Figure G12: Full unedited gel for Supplemental figure S5G, left. (**A**) Copy of the left part of Supplemental figure S5G. (**B**) Indication where the original gel was cropped. (**C**) The original gel.

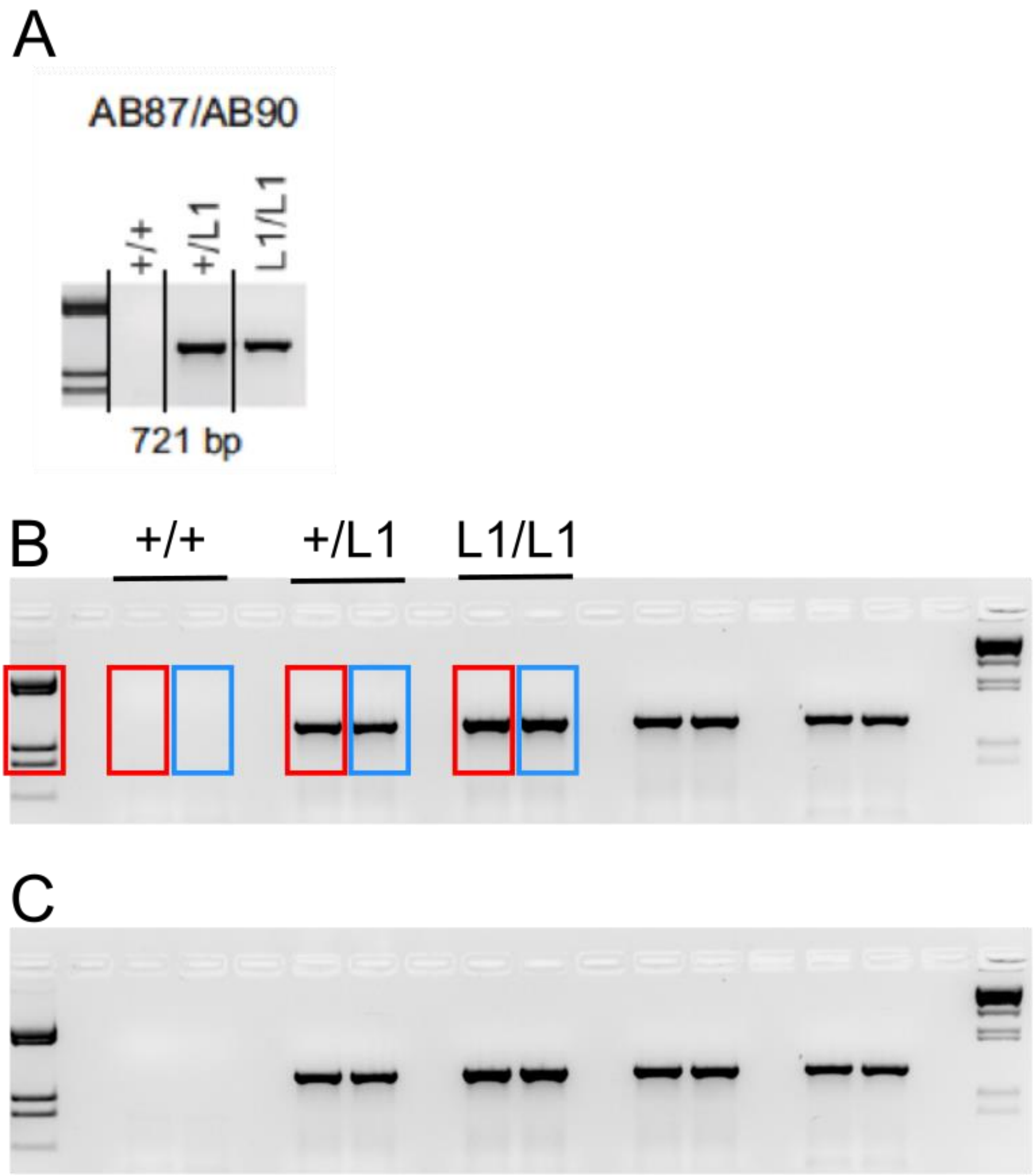

Figure G13: Full unedited gel for Supplemental figure S5G, right. (**A**) Copy of the right part of Supplemental figure S5G. (**B**) Indication where the original gel was cropped. Note that the adjacent red and blue indications are duplicate samples, they contain information on the same sample and one of them is illustrated in figure S5G. (**C**) The original gel. Note that the right part of Supplemental figure S5G is a composite figure, where each lane is an individual crop from the same gel. The parts are displayed next to each other with a solid black line in between.

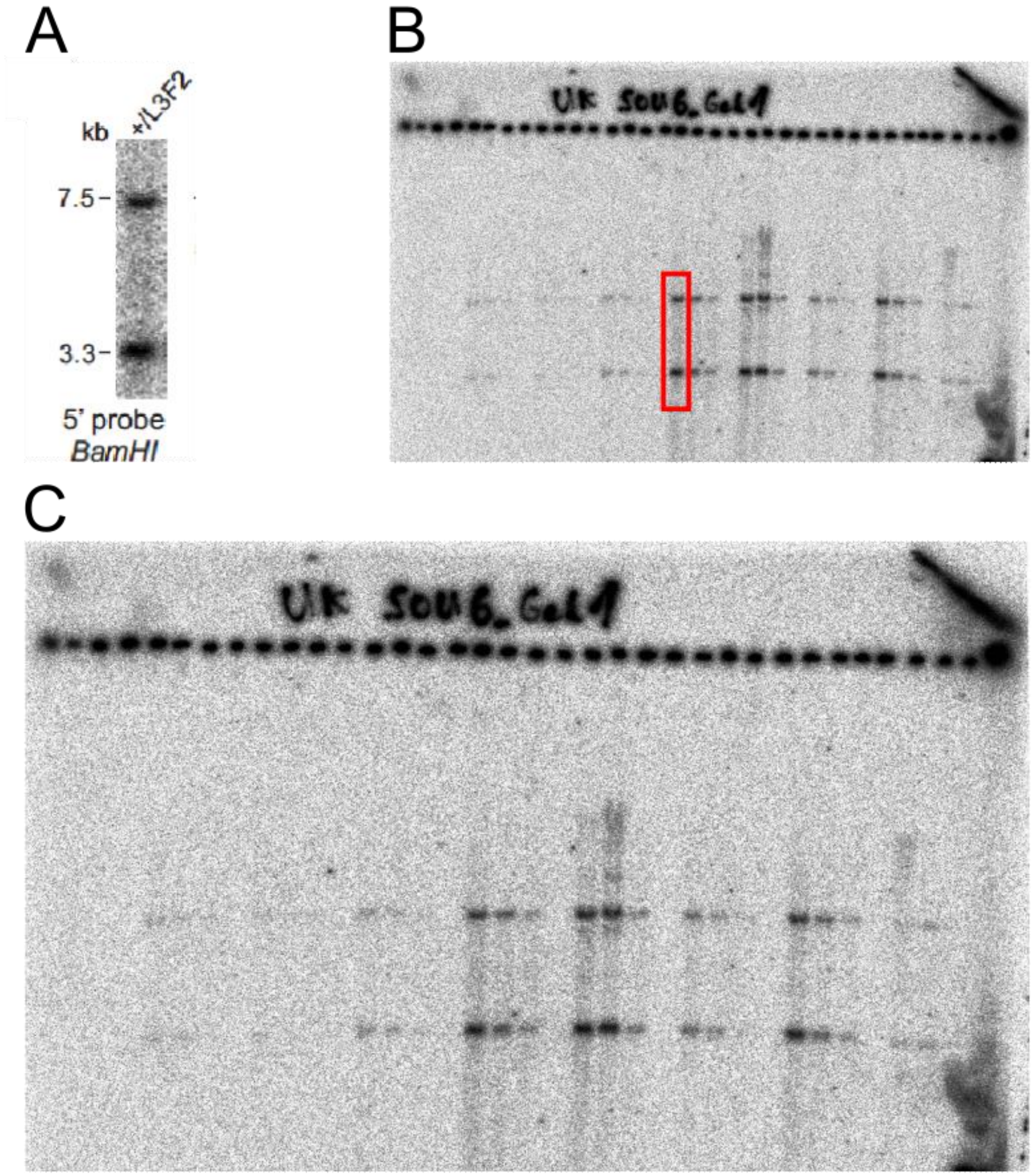

Figure G14: Full unedited gel for Supplemental figure S6D, left. (**A**) Copy of the left part of Supplemental figure S6D. (**B**) Indication where the original gel was cropped. (**C**) The original gel. Note the writing on the image: "UK Sou6_Gel1" has the initials of the experimental scientist, who is shared first author in the manuscript, Sou indicates the methodology, Southern blot, and 6_Gel1 are experimental identifiers.

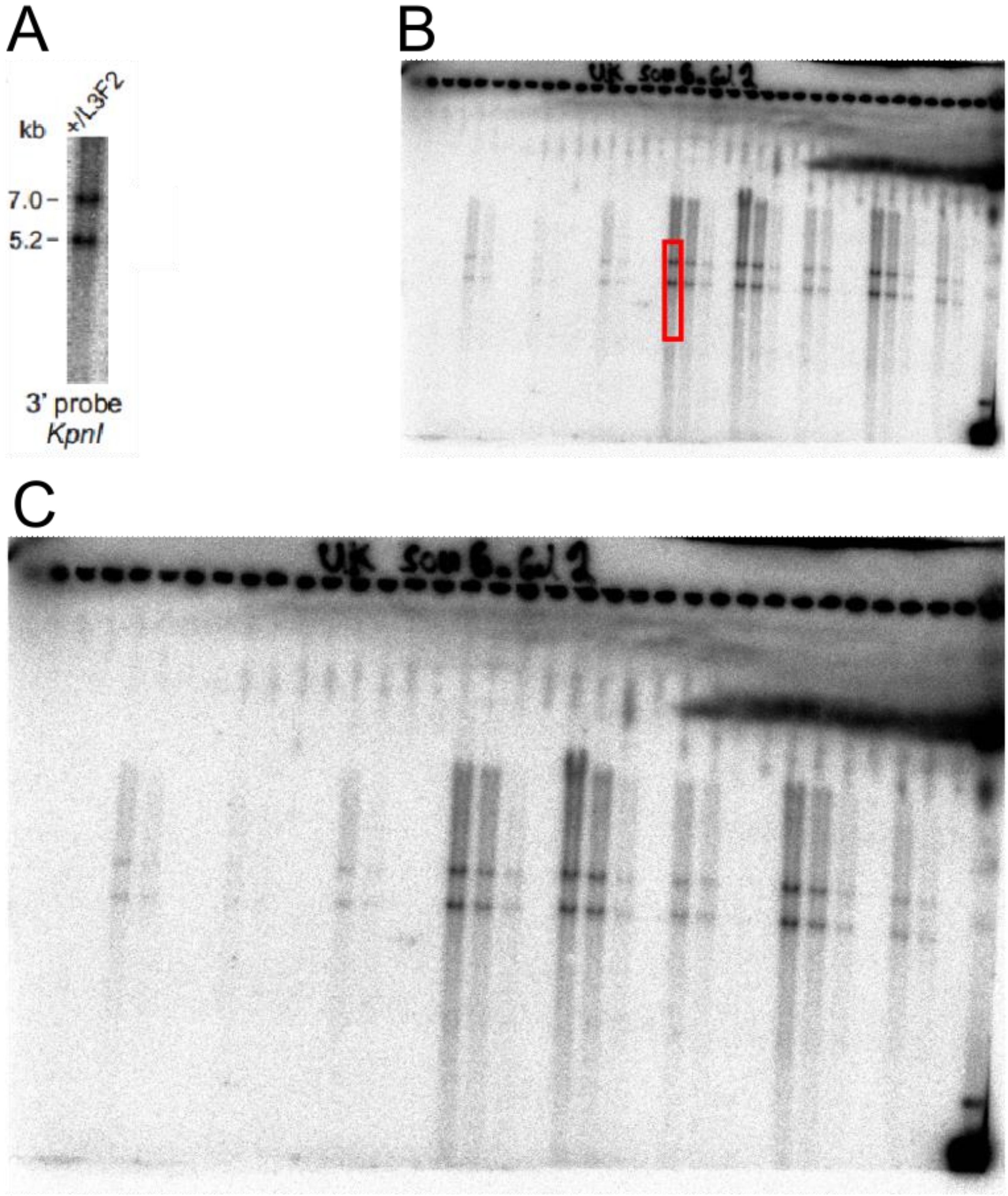

Figure G15: Full unedited gel for Supplemental figure S6D, middle. (**A**) Copy of the middle part of Supplemental figure S6D. (**B**) Indication where the original gel was cropped. (**C**) The original gel. Note the writing on the image: “UK Sou6_Gel2” has the initials of the experimental scientist, who is shared first author in the manuscript, Sou indicates the methodology, Southern blot, and 6_Gel2 are experimental identifiers.

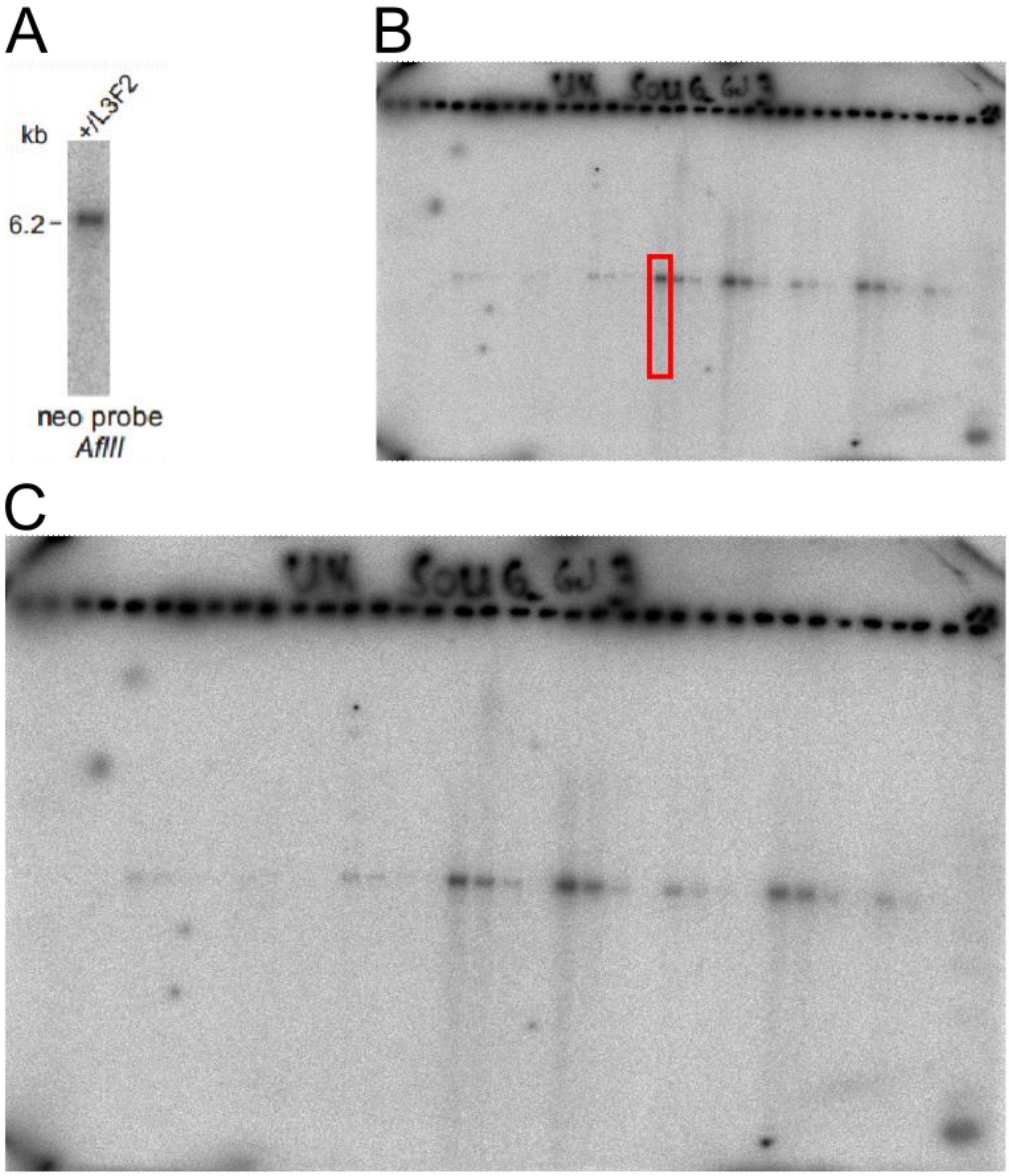

Figure G16: Full unedited gel for Supplemental figure S6D, right. (**A**) Copy of the right part of Supplemental figure S6D. (**B**) Indication where the original gel was cropped. (**C**) The original gel. Note the writing on the image: “UK Sou6_Gel3” has the initials of the experimental scientist, who is shared first author in the manuscript, Sou indicates the methodology, Southern blot, and 6_Gel3 are experimental identifiers.

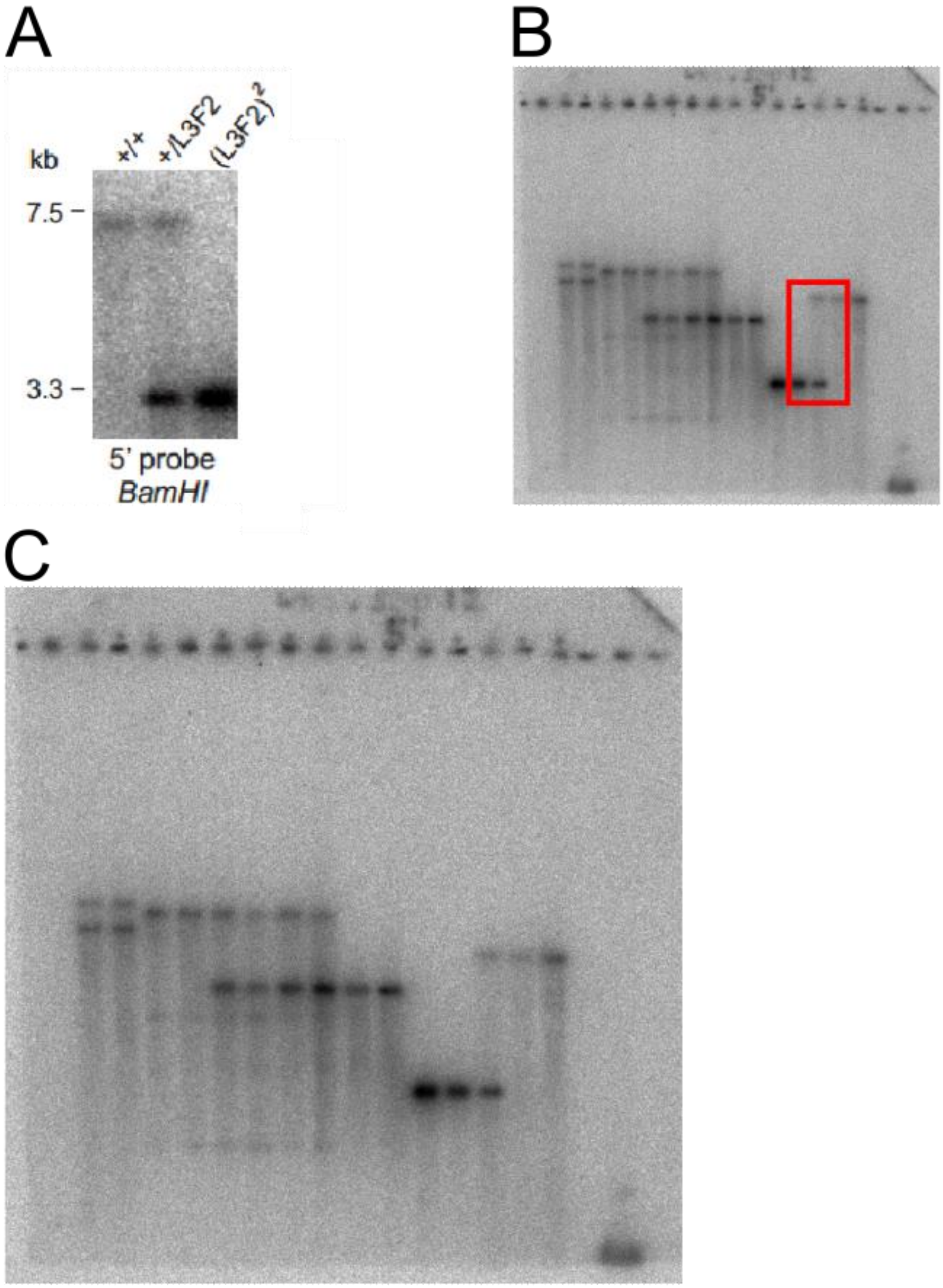

Figure G17: Full unedited gel for Supplemental figure S6E, left. (**A**) Copy of the left part of Supplemental figure S6E. (**B**) Indication where the original gel was cropped. Note that the image in A is horizontally flipped. (**C**) The original gel. Note the writing on the image: “UK Sou12 5’ ” has the initials of the experimental scientist, who is shared first author in the manuscript, Sou indicates the methodology, Southern blot, and 12 is an experimental identifier. 5’ indicates the use of the 5’ BamHI probe used in this experiment.

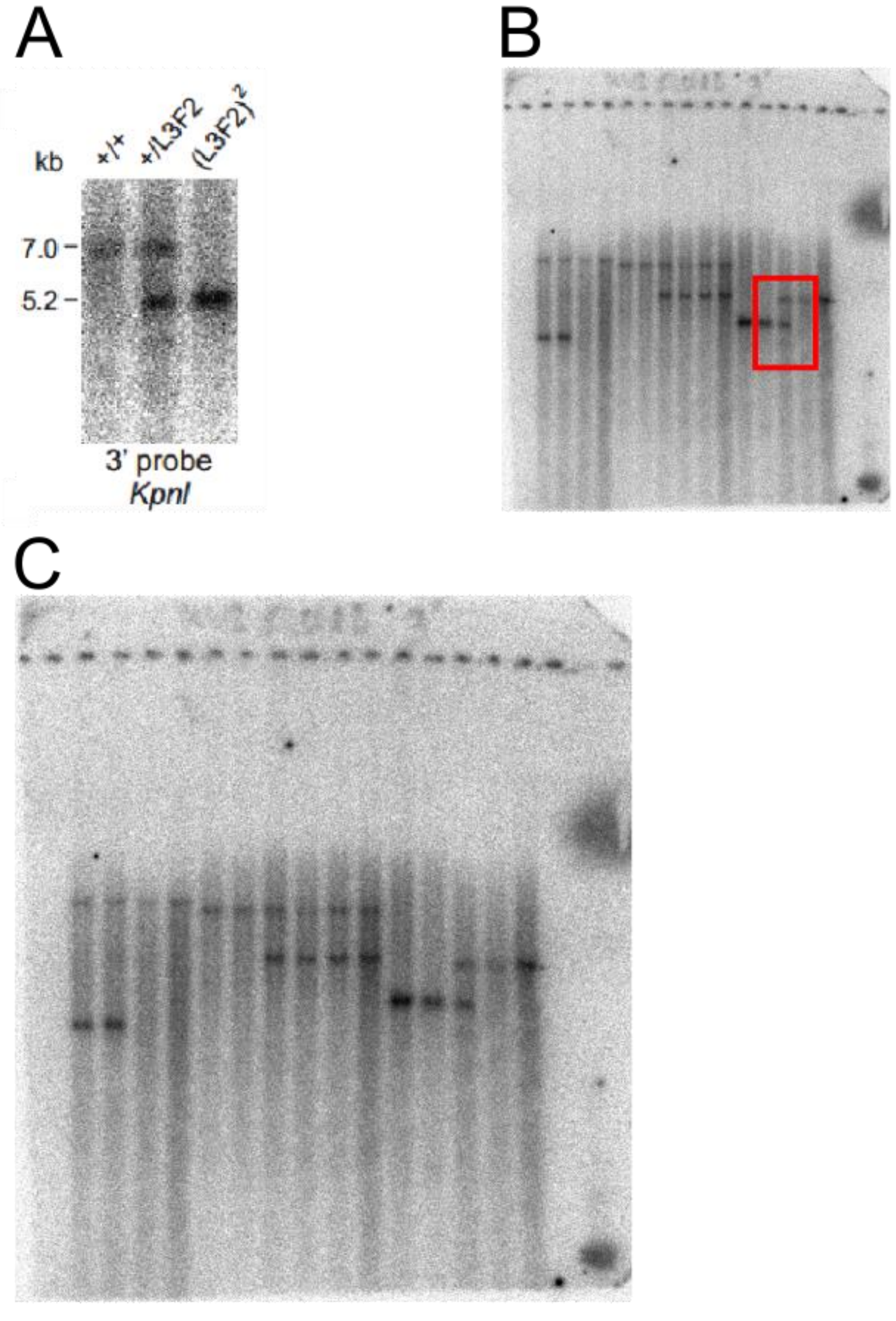

Figure G18: Full unedited gel for Supplemental figure S6E, middle. (**A**) Copy of the middle part of Supplemental figure S6E. (**B**) Indication where the original gel was cropped. Note that the image in A is horizontally flipped. (**C**) The original gel. Note the barely visible writing on the image: “UK Sou12 3’ ” has the initials of the experimental scientist, who is shared first author in the manuscript, Sou indicates the methodology, Southern blot, and 12 is an experimental identifier. 3’ indicates the use of the 3’ KpnI probe used in this experiment.

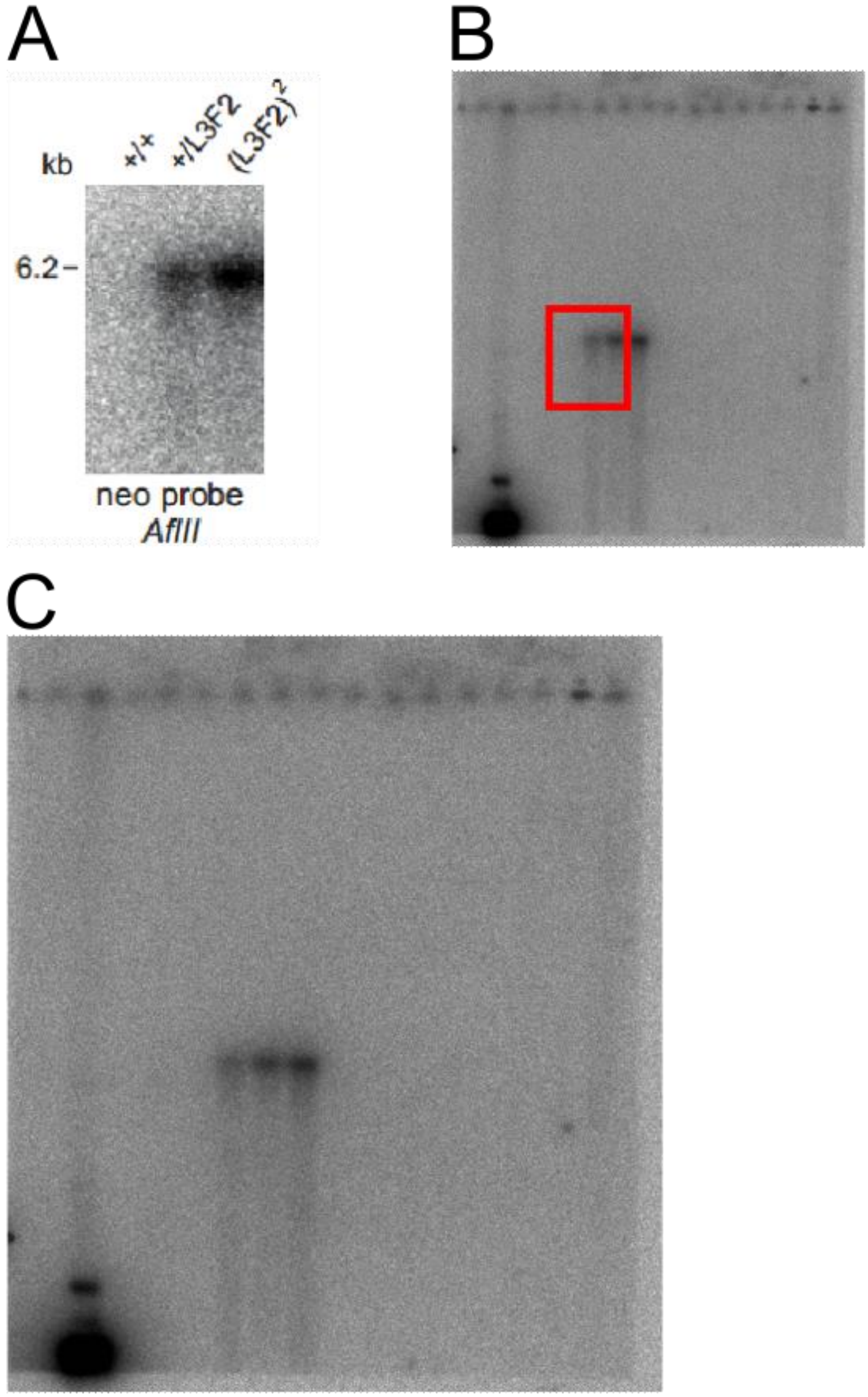

Figure G19: Full unedited gel for Supplemental figure S6E, right. (**A**) Copy of the right part of Supplemental figure S6E. (**B**) Indication where the original gel was cropped. (**C**) The original gel.

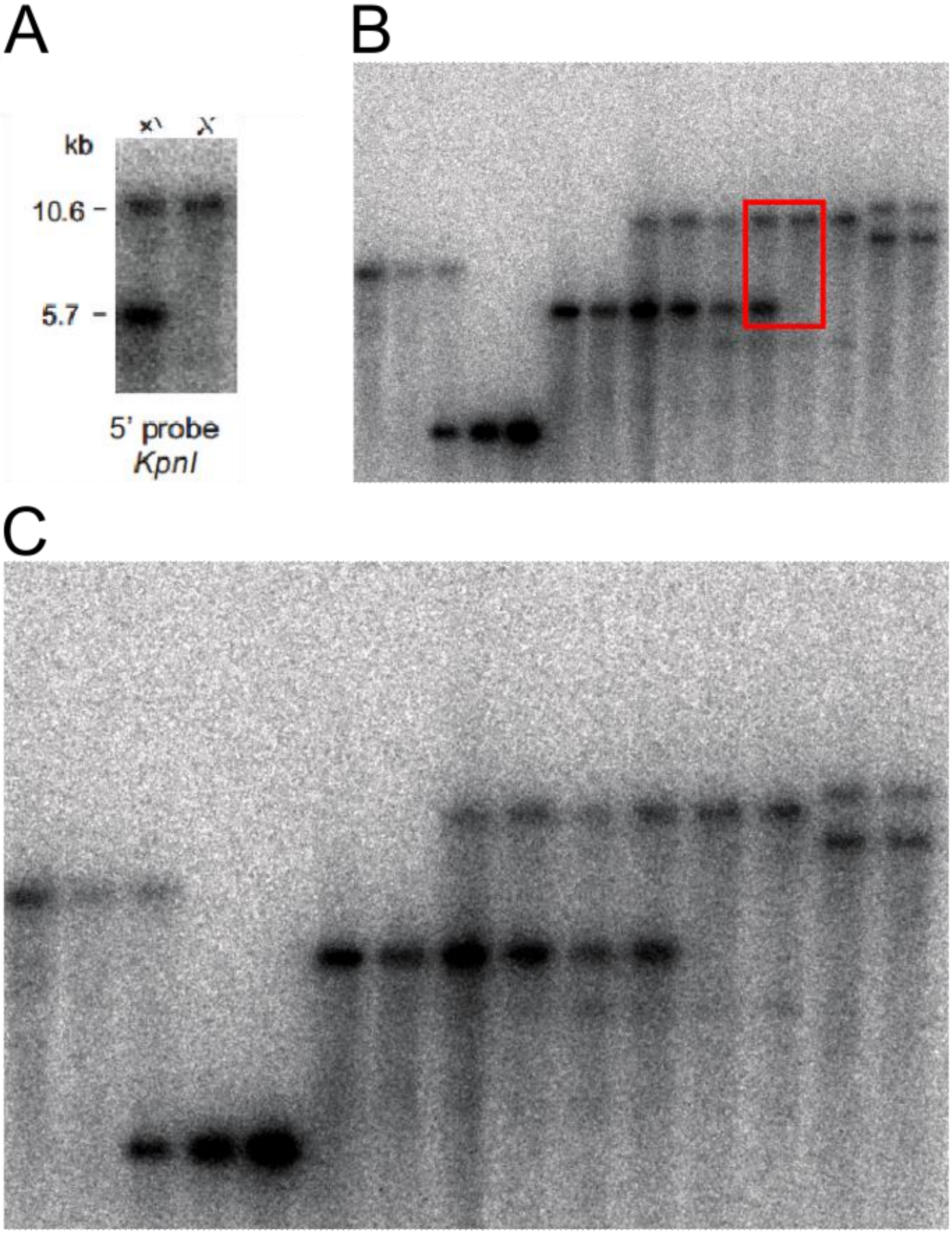

Figure G20: Full unedited gel for Supplemental figure S6F, left. (**A**) Copy of the left part of Supplemental figure S6F. (**B**) Indication where the original gel was cropped. (**C**) The original gel.

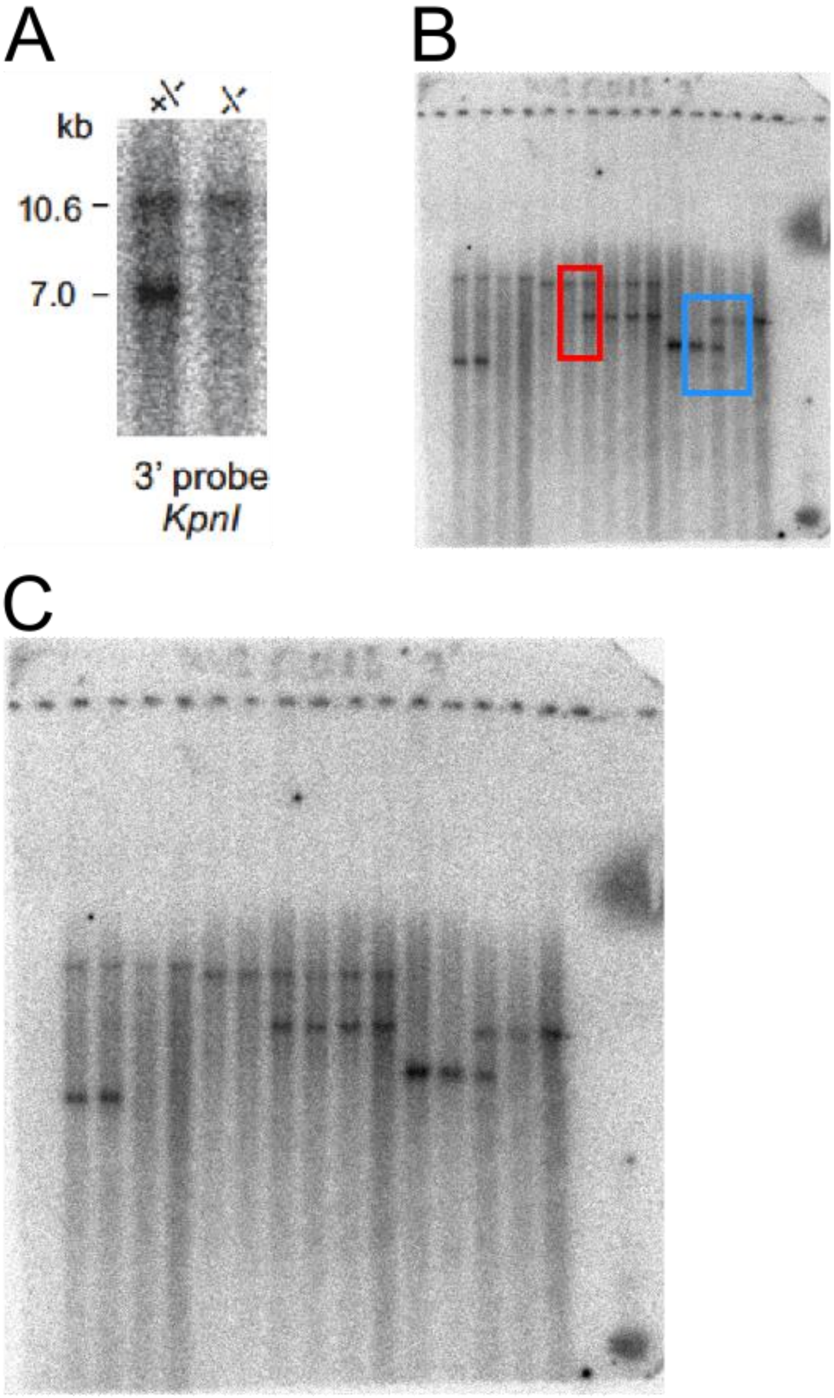

Figure G21: Full unedited gel for Supplemental figure S6F, middle. (**A**) Copy of the middle part of Supplemental figure S6F. (**B**) Indication, in red, where the original gel was cropped. The blue indicated region is used in a different panel, as indicated in figure G18. Note that the image in A is horizontally flipped. (**C**) The original gel. Note that the original gel is identical as the one in figure G18C. The barely visible writing on the image: “UK Sou12 3’ ” has the initials of the experimental scientist, who is shared first author in the manuscript, Sou indicates the methodology, Southern blot, and 12 is an experimental identifier. 3’ indicates the use of the 3’ KpnI probe used in this experiment.

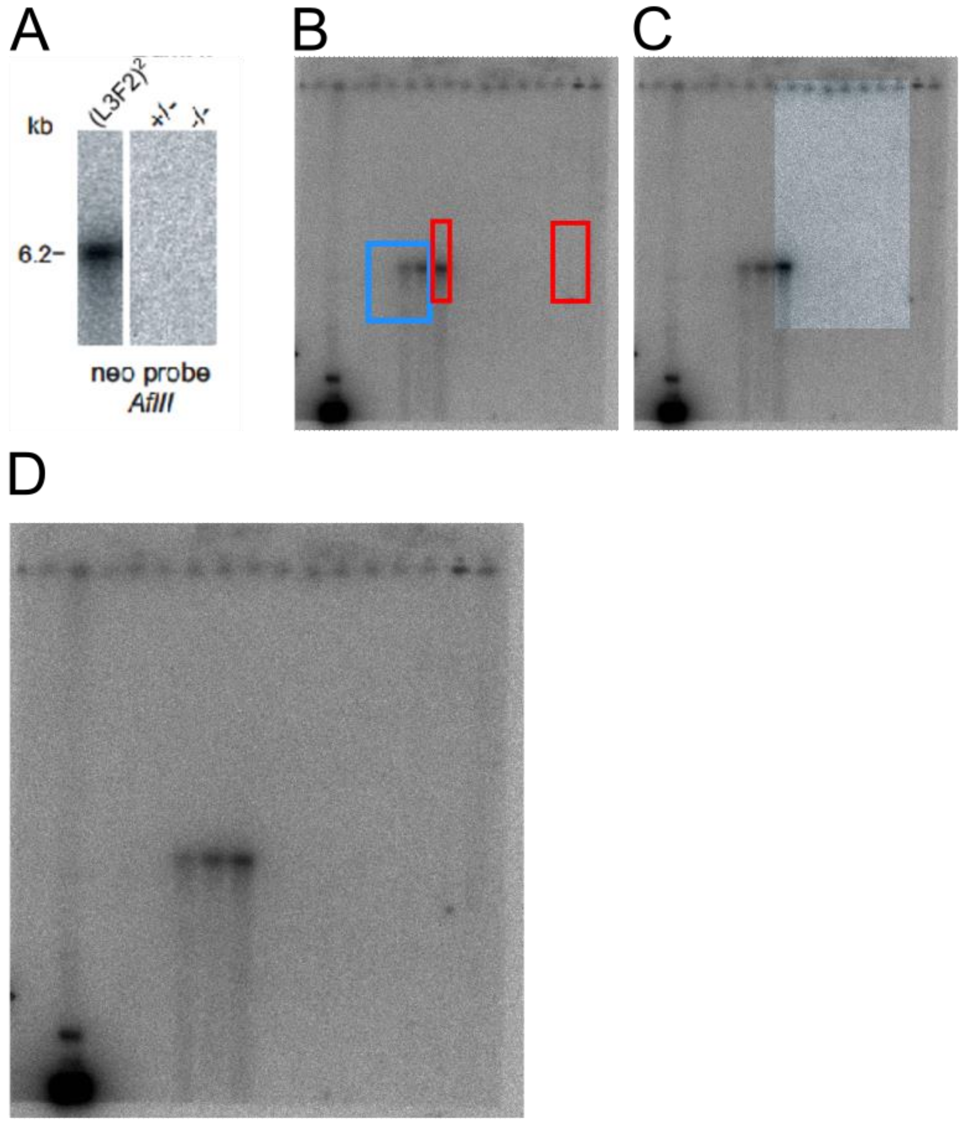

Figure G22: Full unedited gel for Supplemental figure S6F, right. (**A**) Copy of the right part of Supplemental figure S6F. (**B**) Indication in red where the original gel was cropped. Note the indication in blue is the region used in a different panel, described in figure G19. (**C**) An indication of the changes that were made in brightness and contrast on the original figure to optimally visualize eventual faint bands in the indicated +/- and -/- lanes (these bands are not present). (**D**) The original gel. Note that the right part of supplemental figure S6F is a composite image where the $(L3F2)^2$ band is a separate crop from the same gel as the +/- and the -/- bands. Both crops are separated and represented as separate images on the white figure background. Note that the full unedited gel in D is identical to the one in figure G19C.

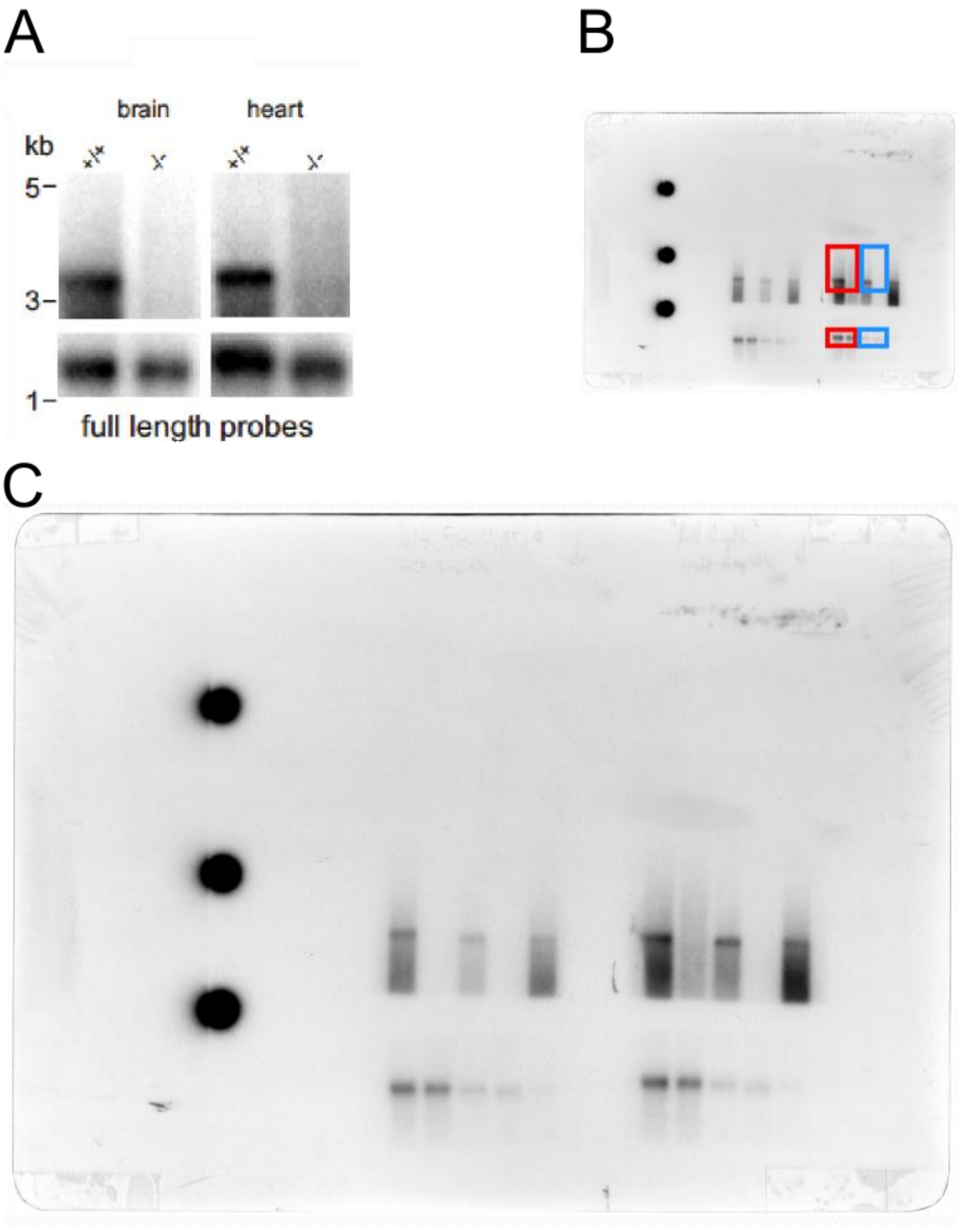

Figure G23: Full unedited gel for Supplemental figure S6G, left. (**A**) Copy of the left part of Supplemental figure S6G. (**B**) Indication where the original gel was cropped, the top bands correspond to the presence of OCaR1 and the bottom bands to GAPDH. The red regions indicate the lanes with samples from the brain, while the blue regions indicate samples from the heart. (**C**) The original gel.

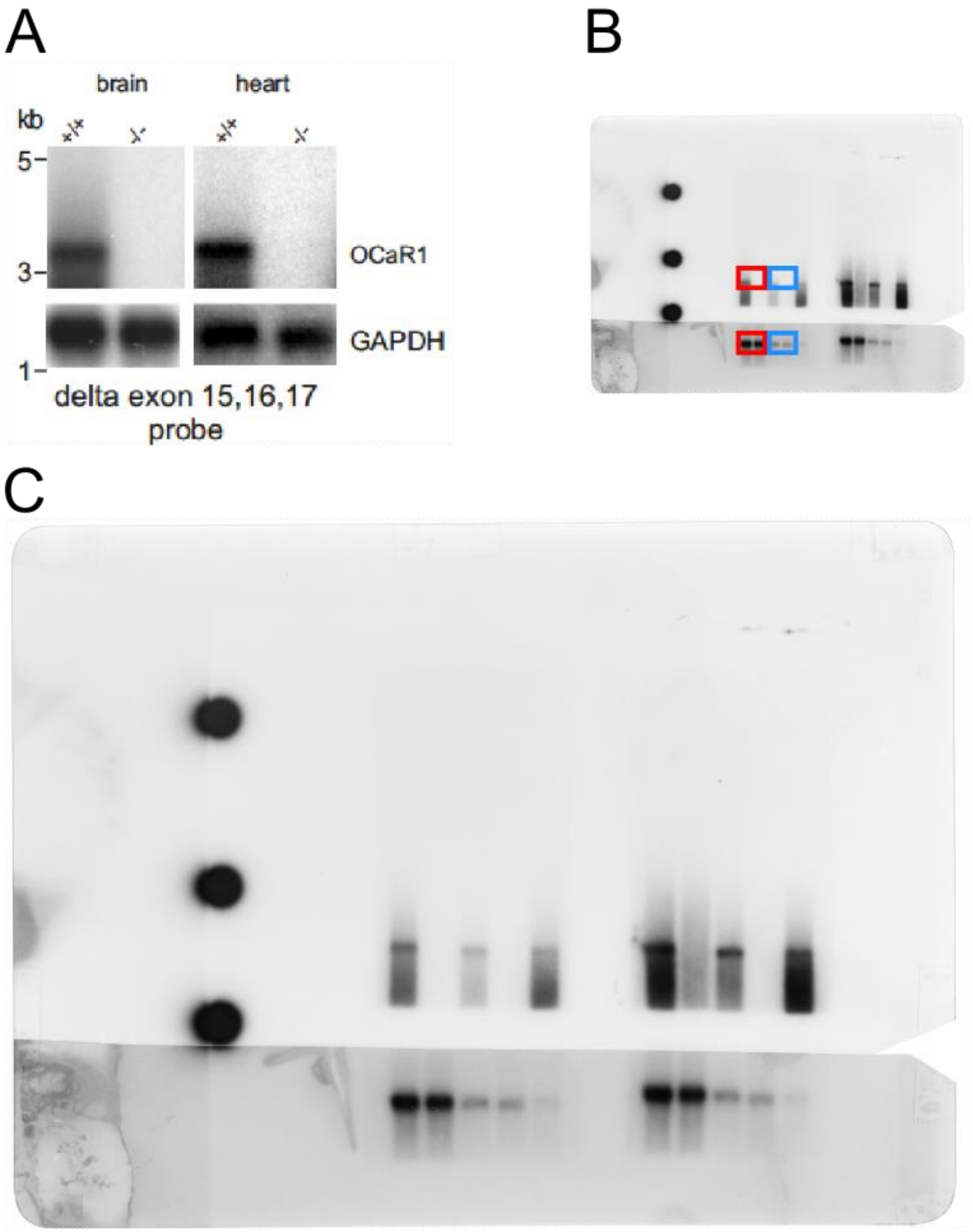

Figure G24: Full unedited gel for Supplemental figure S6G, right. (**A**) Copy of the right part of Supplemental figure S6G. (**B**) Indication where the original gel was cropped, the top bands correspond to the presence of OCaR1 and the bottom bands to GAPDH. The red regions indicate the lanes with samples from the brain, while the blue regions indicate samples from the heart. (**C**) The original gel. Note that the original gel in C and the original gel in figure G23C are different hybridizations of the same blot.
